# Supplementary material for: Beyond Alzheimer’s story: How an engineered molecule gained an endogenous essence
Source: PNAS Nexus. 2026 Oct 20;5(10):pgag320. doi: 10.1093/pnasnexus/pgag320 (PMC13681328; doi:10.1093/pnasnexus/pgag320)
Supplement: Supplementary file 1 [file pgag320_supplementary_data.pdf]

SUPPLEMENTARY INFORMATION

Beyond Alzheimer's story: how an engineered molecule gained an endogenous essence

Arthur T. Kopylov, Ruslan Yu. Yakovlev, Aleksei V. Trukhin,

Yuri M. Poluektov, Anastasia A. Anashkina, Sergey A. Kozin

The research was organized and funded by
LIFEMISSION, LLC (Moscow, Russia)

Table of Content

Section 1. Structural Characterization of a Signal: Basic Spectrum of The Precursor Ion and Its Fragmentation Pattern 2

Section 2. Mass Spectrometric Conditions for Peptide Registration 7

2.1. Chromatogram and Spectrum Properties 10

2.2. Signal Significance Threshold 13

Section 3. Calibration Curve 15

Section 4. Determination of Limits 19

Section 5. Extraction of HAEE 21

Section 6. Recovery of HAEE After Extraction and Matrix Influence Factor 25

Section 7. Study of Stability of the HAEE Substance 27

7.1. Continuous Storage: Stability of HAEE In the Environment of a Pure Solution 27

7.2. Continuous Storage: Stability of HAEE In the Biological Matrix Environment 31

7.3. Interval (cyclic) Stability of HAEE In Working Solution and Biological Matrix at Low Temperatures 34

Section 8. Carryover 36

Section 9. Accuracy and Reproducibility of Measurement 42

Section 10. Linearity of Dilution 45

Section 11. Parallelism 47

Section 12. Cross-platform Validation. Measurement of Endogenous HAEE In Human Blood Plasma Samples Using UPLC-QQQ-SRM. 49

Section 13. Analytical research 53

13.1. Description of the population 53

13.2. Results of instrumental measurements 54

13.3. Determination of sensitivity, specificity and predictive value 56

13.4. Comments and conclusions on the diagnostic characteristics of the test 59

Section 14. ^{1}H NMR and LC-UV-MS analysis data for Ac-HAEE-NH_2 and Ac($^{13}C_2$)-HAEE-NH_2. 60

References 66

List of Abbreviations (in alphabetic order) 66

Section 1. Structural Characterization of a Signal: Basic Spectrum of The Precursor Ion and Its Fragmentation Pattern

To register a signal of a peptide, it is necessary to obtain information on structural characterization of the peptide and its fragment ions pattern. The full fragmentation spectrum was obtained and recorded under the conditions of chromatographic separation on a C18 column, but without preliminary optimization of the chromatographic separation conditions (Figure 1). Under non-optimized conditions, the retention time on the column for the non-labeled standard was RT = 0.57 (±4.7%). The standard was introduced onto the C18- column heated at 50°C at a concentration of 4 ng/ml and separated at a flow rate of 0.3 ml/min and in a gradient of mobile phase A (0.1% formic acid, aqueous solution, pH ~ 2.6) and mobile phase B (0.1% formic acid in acetonitrile) for 5 minutes, including the post-analytical equilibration time.

A typical fragmentation spectrum for the standard displayed fragment ion peaks, the distance between which corresponds to the monoisotopic mass of adjacent amino acid residues or their dehydrogenated/deaminated derivatives. Since the substance under study is a peptide, it is possible to calculate theoretical fragment ions with a correction for the activation energy type. The decomposition of peptide fragmentation occurs along the peptide bond between the nitrogen and carbonyl carbon atoms (in the CID activation mode) with the formation of sequential N-terminal ions (*b*-type ions) and C-terminal ions (*y*-type ions) [a].

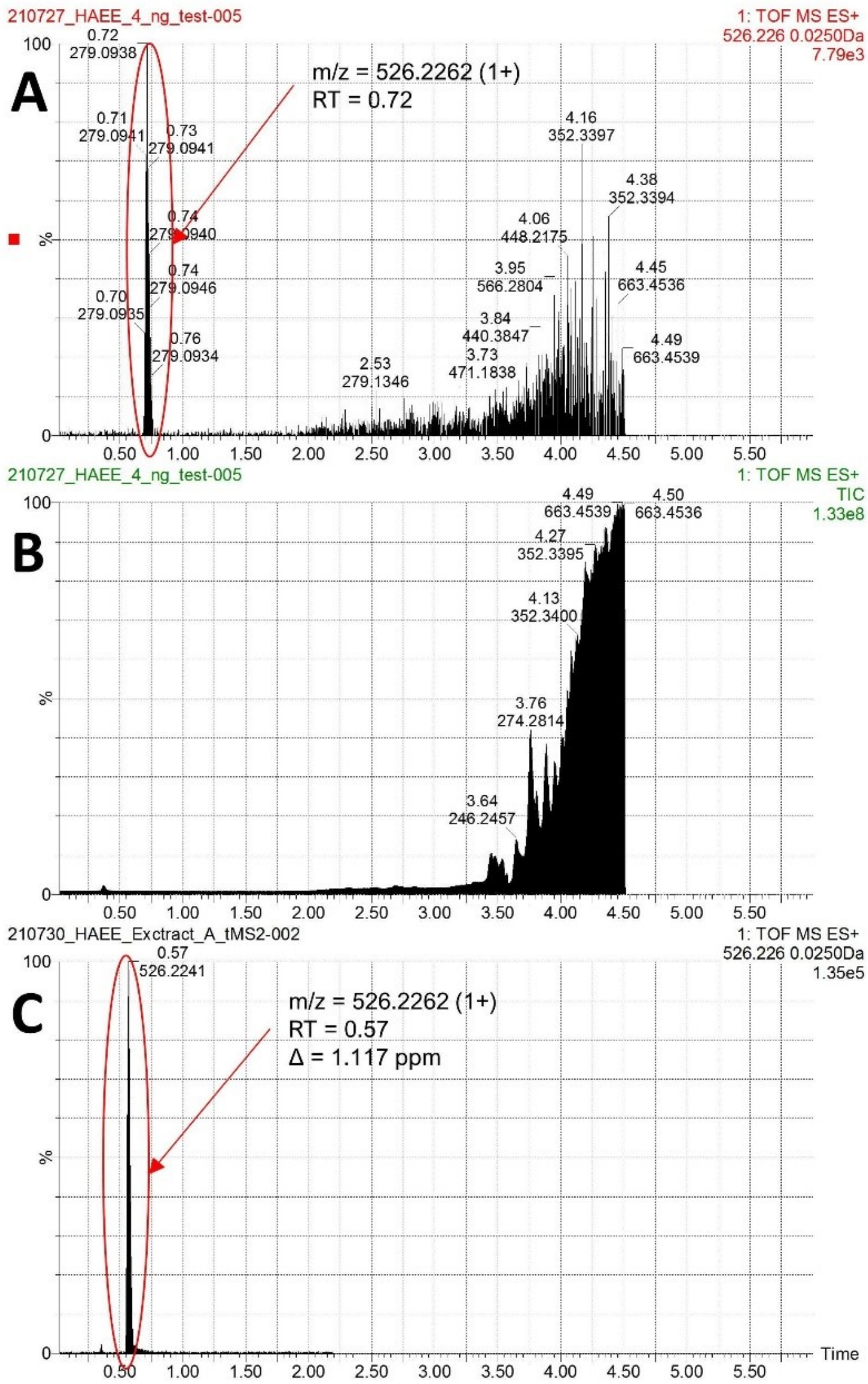

Figure 1. Chromatographic separation of the peptide standard (concentration 4 ng/mL, loading volume on the column was 5 µL, loading amount was 20 pg) on the stationary phase C-18 (Zorbax™ Eclipse Plus C18 (Rapid Resolution HD) manufacturer Agilent, Inc, column geometry 2.1 × 50 mm, particle size 1.8 µm, pore size 120 A; column serial number: SN USDAY20100, PN 959757–902, LN B14245) in a simplified gradient. **(A)** Isolated ion current chromatogram in full frontal scan mode for m/z = 526.2262 (1+) with retention time RT = 0.72 minutes; **(B)** Total ion current chromatogram in full-MS scan mode; **(C)** Isolated ion current chromatogram of m/z = 526.2262 (1+) with retention time RT = 0.57 min in tandem scan mode. The average mass measurement error was +1.117 ppm.

With the CID activation, it is also possible to produce an *a*-type N-terminal ions as a result of breaking the bond between the C - Cα atoms, but their yield is scarce, since it requires specific conditions in the ionization source chamber. It should be noted that *b*-type ions can be dehydrated, whereas y-type ions can be represented by ammonium salts. However, in contrast to the HCD type of activation, in the CID fragmentation mode the yield of such derivatives is barely used. Thus, the fragmentation spectra obtained in the CID activation mode are explicit and are more suitable for determining the structure.

[a]The formation of *c/z*-type ions is possible when using ETD / ECD activation type, whereas the formation of *a/x*-type ions is possible when using hard ultraviolet photoionization and high temperatures.

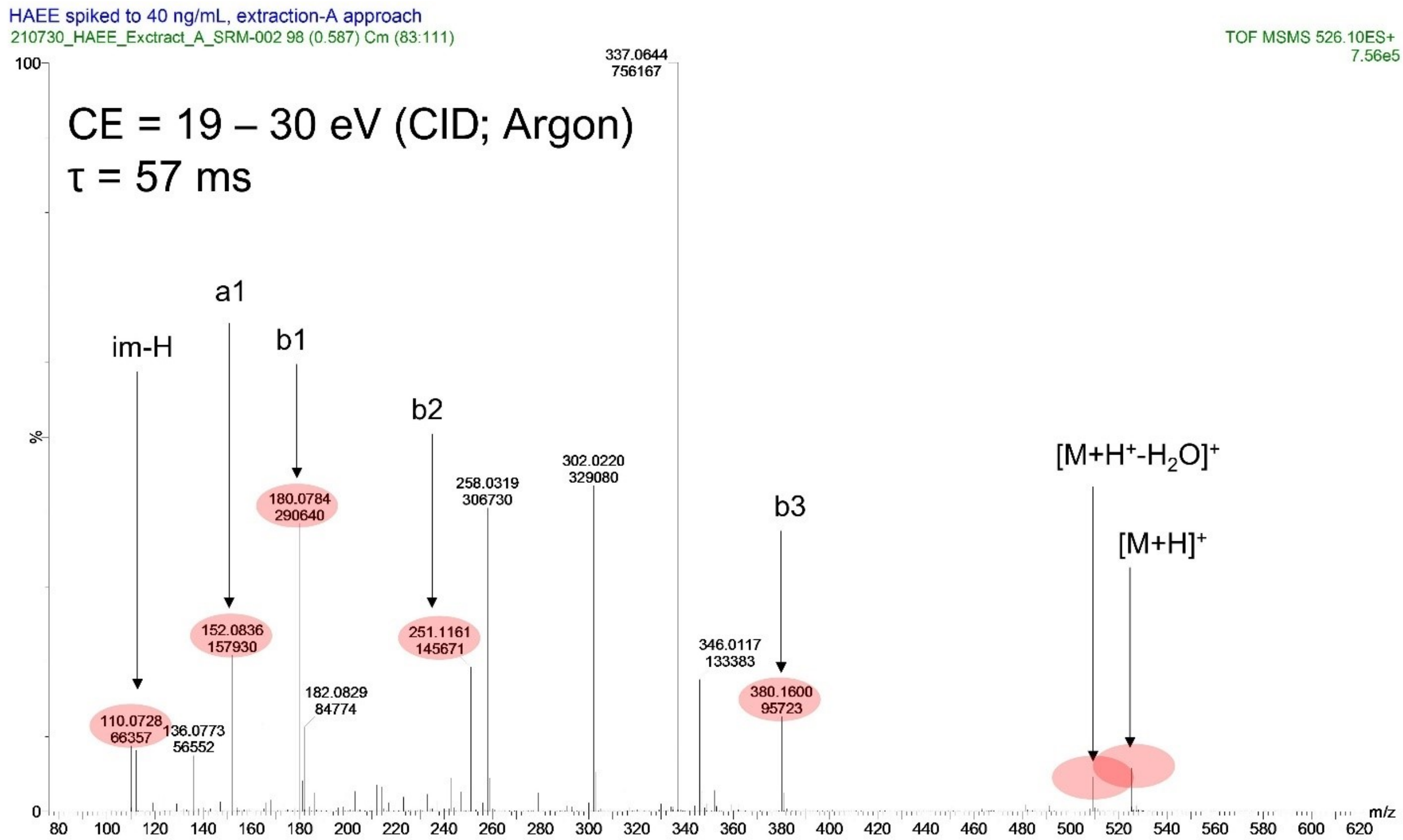

Figure 2. Typical fragmentation spectrum of the HAEE peptide standard under non-optimized conditions in CID mode with activation energy ranging from 19 to 30 eV. The fragmentation spectrum was obtained from the parent ion with m/z = 526.2262 (z= 1+). Spectrum scan is averaged over 29 scans, each scan with a duration of acquisition time for 75 ms.

Figure 2illustrates fragment ions with m/z = 251.1139, m/z = 152.0818, and m/z = 380.1565. The y-type fragments are barely generated because the C-terminus of the peptide carries an amide group, which is characterized by extremely low proton affinity. Yet, one can note the presence of an immonium salt (inner salt) of histidine (m/z = 110.0728) as a result of decomposition of the N-terminal acetyl group. Under these conditions, the most effective activation energy range was found to be between 19 and 30 eV, while the focusing voltage in the ionization source was used in a narrow range from 11 to 21 V. We also note that during the fragmentation and ionization, 2-oxoimidazole derivatives of the amino acid residue of histidine are not generated, which indicates the stability of tetrapeptide under the initial conditions casted for the ionization source. It should be taken into account that the spectrum presented in Figure 2 was obtained under the baseline scenario before the final optimization of conditions. Under these conditions, the baseline fragments were characterized by un stable amplitude in height (amount of charges per second), and, consequently, instability of ranking/ordering by fragmentation priority. Subsequently, the development of fragmentation conditions led to a stable portrait of the fragmentation spectrum, which was also transferred to the registration conditions of the heavy-labeled [$^{13}C_2$]-standard of HAEE.

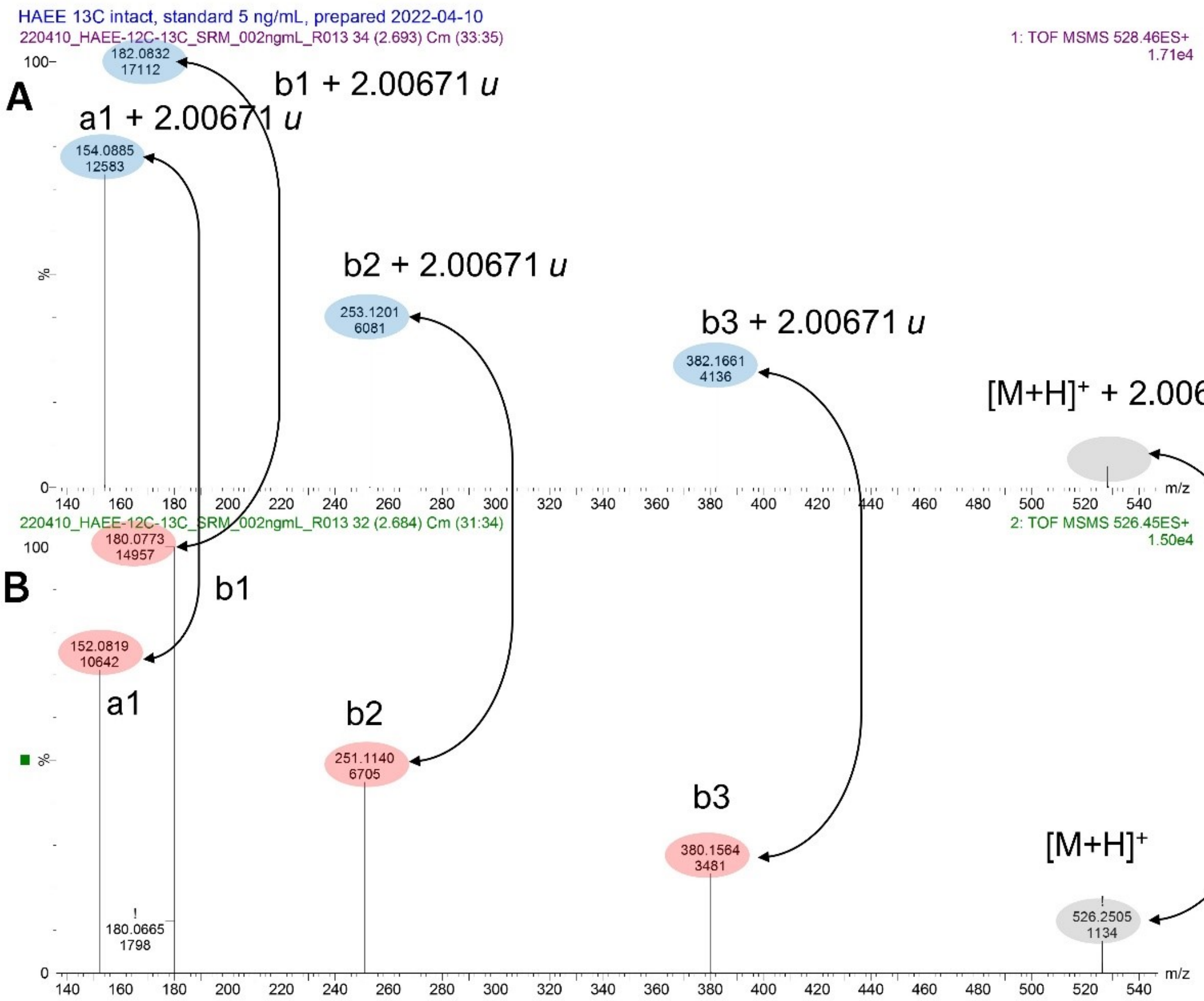

Figure 3. Fragmentation spectrum of the heavy-labeled standard (A) and non-labeled standard (B) of HAEE under optimized registration conditions. Both standards are in an equimolar mixture (2.5 ng/ml of each standard in an aqueous solution of 0.5% formic acid).

Under optimal recording conditions in the MRM mode (Figure 3), a stable spectrum of standards (labeled and non-labeled) with a base peak m/z = 180.07 and m/z = 182.08 (hereinafter, the nominal value is indicated) is observed. One and the same time, (Figure 3), a shift in m/z-values of fragment ions by 2.00671 *u* (atomic mass units), corresponding to the substitution of the isotope [^{13}C] of two atoms, is observed for the labeled standard. It is also obvious that due to the good isotopic resolution and the small mass difference (only 2 atomic mass units) there is a risk of isotopic interference between labeled and non-labeled molecules in the region of the third and first isotopes, respectively.

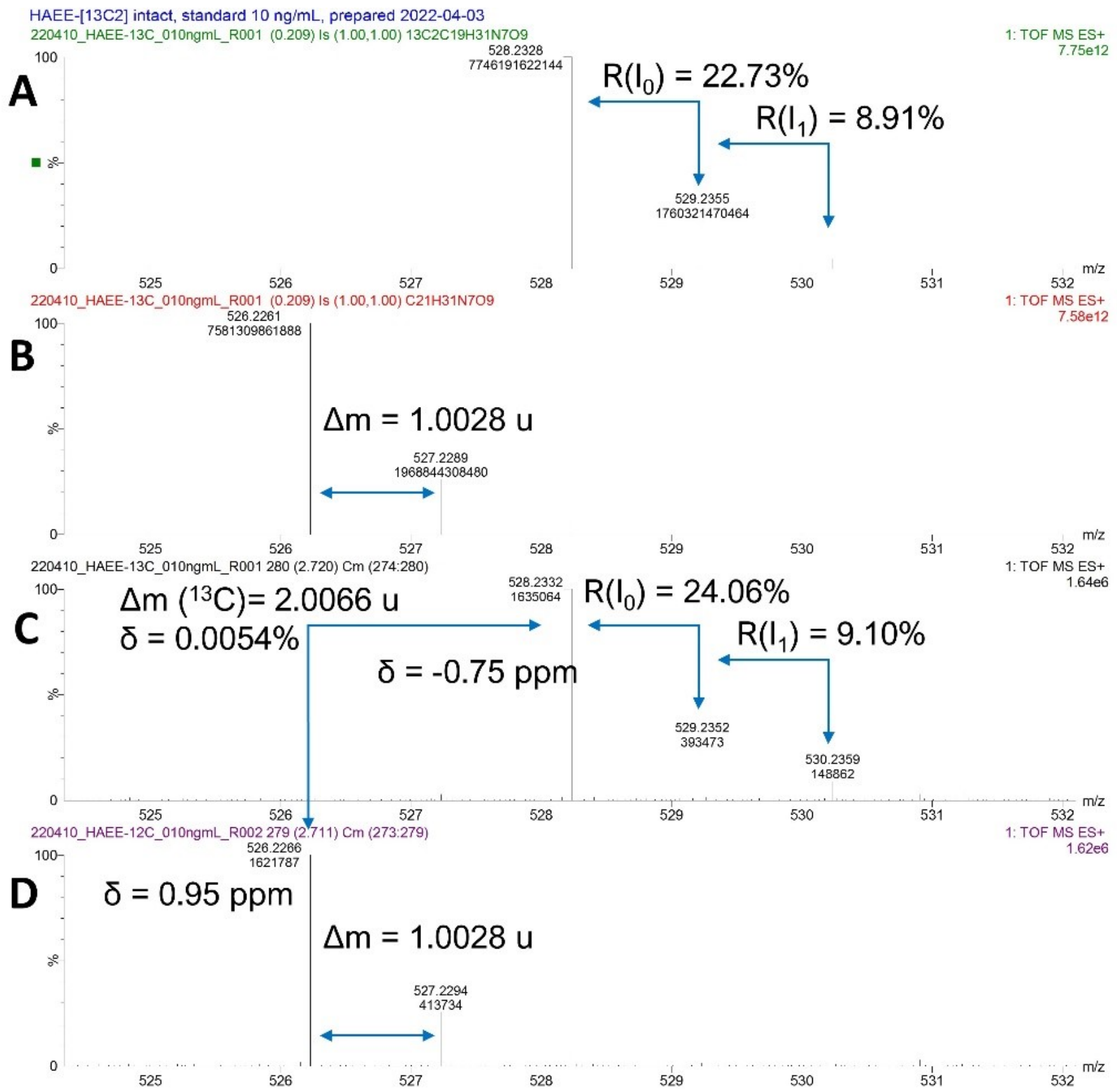

Figure 4. Isotopic distribution of labeled and non-labeled standards. (A) Simulated spectrum of isotopic distribution m/z = 528.2328 (1+) for the standard with the inclusion of two [^{13}C] isotopes of the carbon atom; (B) Simulated spectrum of isotopic distribution m/z = 526.2261 (1+) corresponding to the intact peptide; (C) Empirical spectrum of isotopic distribution of the non-labeled standard and (D) heavy-labeled standard in equilibrium (10 ng/ml) ratios in an aqueous solution of 0.5% formic acid. The accuracy of mass measurement for the labeled standard is -0.75 ppm; the accuracy of mass measurement for the non-labeled standard is +0.95 ppm (the accuracy of mass measurement of the third isotope is +1.62 ppm). The theoretical contribution of the third isotope of non-labeled standard to the height (counts) of the first isotope of the labeled standard A2 is δ=4.9%; the empirical contribution is δ=5.9%.

The recorded m/z-values of the standards fully correspond to the simulated (model) distribution of isotopes, which indicates that the empirical isotopic composition corresponds to the theoretical one in each case under study (Figure 4). However, for the first (basic) isotope of the heavy-labeled standard m/z = 528.2328 (Figure 4A), whereas for the third isotope of the non-labeled standard m/z = 528.2312 (Figure 4B), i.e. the difference is only Δ m = 1.6 mDa (or 3.028 ppm). According to the isotope distribution model, these isotopes is related as 0.04985, i.e. the contribution of the third isotope of the non-labeled standard is up to 4.9% to the peak height of the first isotope of the labeled standard. Empirical data have shown that the contribution can be up to 5.9%, which is due to the ionization effect and the concentration effect (measurements on standards at a concentration of 10 ng/ml). Thus, the total final contribution of isotope interference at a concentration of 10 ng/ml can be up to 0.59 ppb (or up to 590 ppt) in terms of the amount of substance, which is an insignificant value and can be neglected.

To increase sensitivity and reduce potential isotope interference at a high concentration, ion transmission was optimized by maneuvering and adjusting the settings of the Step Wave ™ion funnel unit. Changes to settings are made manually with direct sample injection at flow rate of 10μl/min.

Step Technology Wave ™ is structurally two coaxial ion funnels made of a series of orthogonal electrodes with variable geometry. The potential difference makes it possible to accelerate the ion conductivity in the electromagnetic field and regulate the ion velocity depending on the mass of their quasi-molecular ion. As a result of multi-stage optimization, the following values were obtained: on the first ion funnel, the velocity is 400 m/s, the amplitude is 9.5 V; on the second funnel, the velocity is 435 m/s, the amplitude is 16.5 V; forward voltage bias is 18 V; potential difference on the first lens is 4.2 V, potential difference on the second series lens is 0.9 V; the velocity on the ion conductor is 367 m/s, the amplitude is 1.2 V.

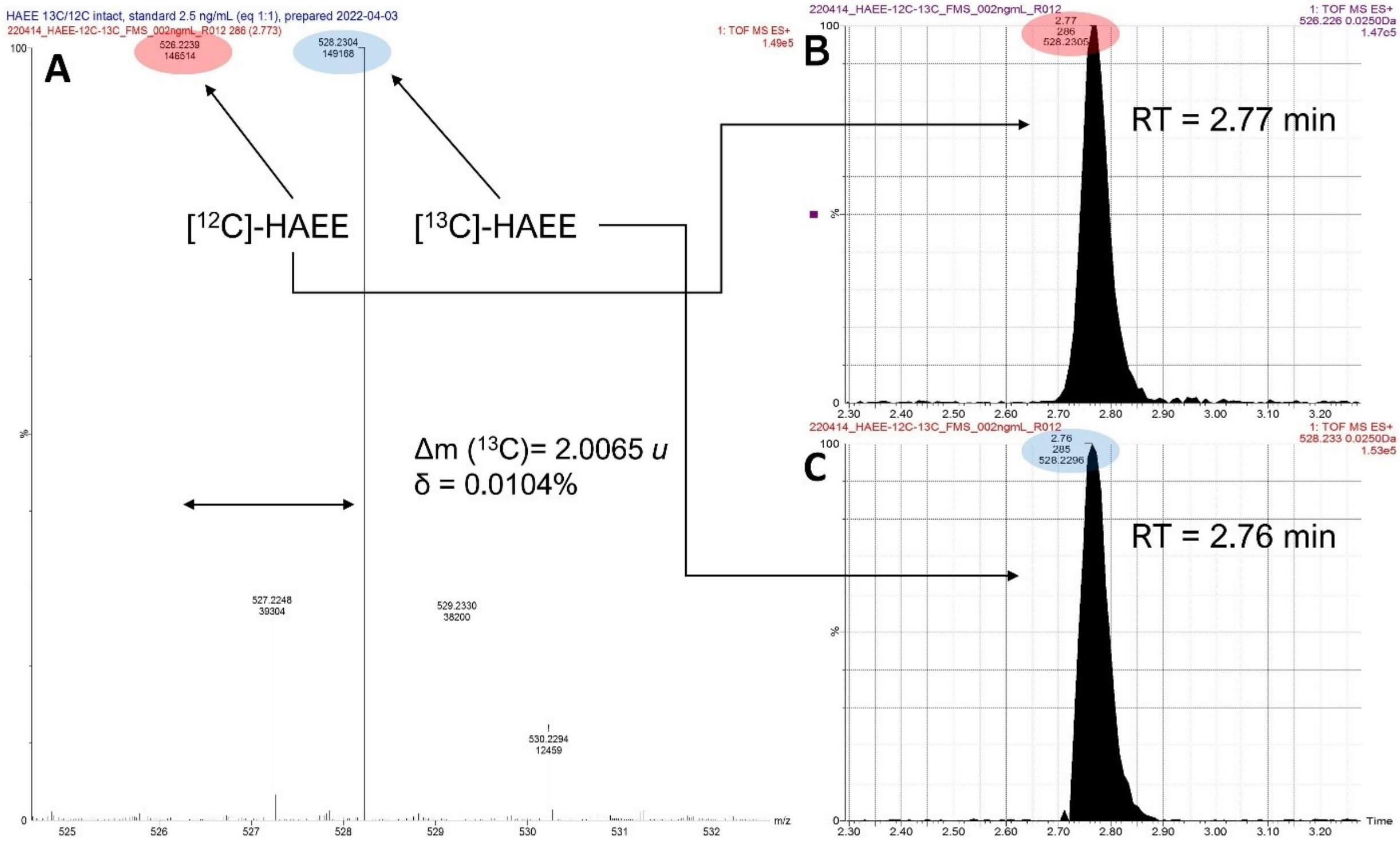

Figure 5. Spectrum of parent ions and separation chromatograms under optimized ion transmission conditions. (A) Full MS spectrum of isotopic resolution of labeled and non-labeled standards in an equilibrium mixture (2 ng/ml of each standard). Optimized ion transmission conditions made possible to level out the contribution of the isotopic distribution of non-labeled standard to the height or area of the first isotope of labeled standard. The total contribution of isotopic interference does not exceed 0.7% under optimized ion transmission conditions. (B) Isolated ion current chromatogram of non-labeled standard and (C) labeled standard.

As a result of optimization, the intensities of the parent ion peaks were almost completely aligned even at low concentration s (Figure 5). In particular, test of the equilibrium mixture of standards (2 ng/ml of each standard in the mixture) in a 0.5% formic acid solution showed that the intensities of the first isotopes of each standard differ within 2.3%, while the contribution of the third isotope of the non-labeled standard to the first isotope of the labeled standard diminished to less than 0.7% (Figure 5). At the next stages, the ion optics were optimized to register the peptide with maximum sensitivity in the MRM mode.

Further instrumental optimization based on the tuning of RF amplitude, the combined effect of the focusing voltage and its offset, the time of a complete scan cycle, the voltage on the electron multiplier, and the discrete ion accumulation time in the high-resolution MRM mode. Tuning of these parameters was carried out under conditions of the optimal elution gradient, the conditions for which will be specified in a special section below. Optimization of RF was performed at 270 V (default value), at 400 V and at 500 V. The assessment was performed based on the peak area of the parent ion at a concentration of 4 ng/mL in an aqueous solution of 0.5% formic acid in five technical replicates (Figure 6).

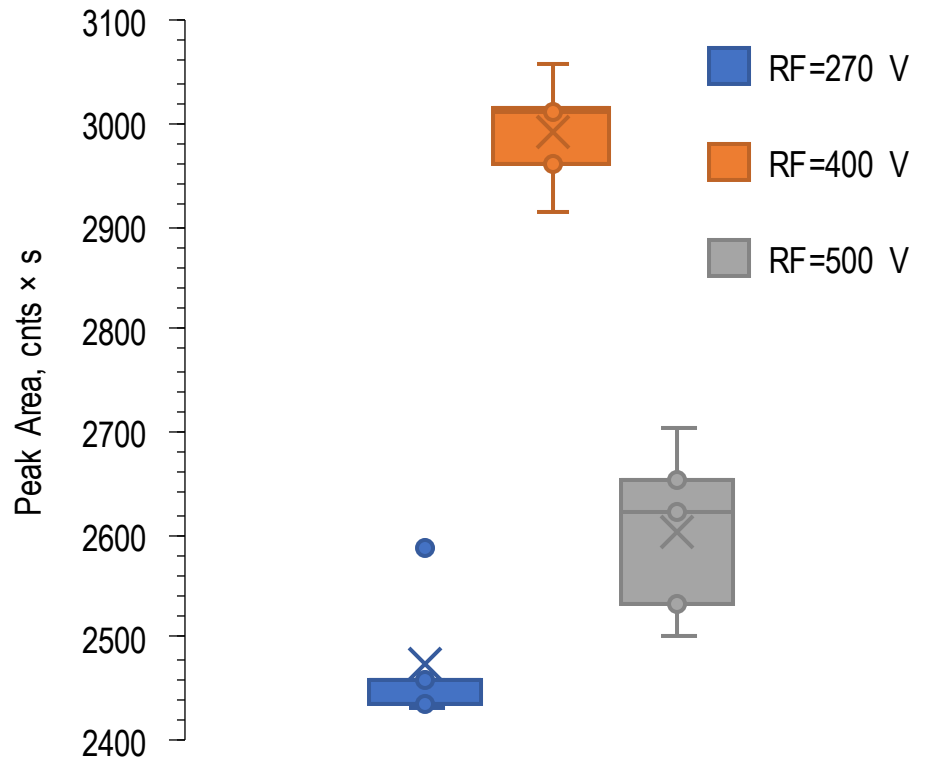

Figure 6. Peak area distribution of the parent ion m/z = 526.2262 (1+) of HAEE standard at a concentration of 4 ng/ml depending on the RF voltage. Full scan mode.

The maximum peak area is observed at 400 V on RF (A = 2992 cnt × s ±1.84%; inclusive median in quartile calculation). As the potential difference increases to 500 V, the signal (peak area) decreases by 13% (relative to the area at 400 V) and approaches the area value observed at the default voltage (270 V). Also, the signal at 500 V is characterized by the maximum instability (covariance is 3.27%). Thus, the potential difference parameter on the quadrupole was fixed at 400 V, as optimal and stable (Figure 6).

Next, the optimization of focusing voltage and its offset were performed. These parameters are always selected in pairs, and the offset by module should always be greater than the nominal voltage value. The most important of the two parameters is the intrinsic focusing voltage. The focusing voltage was varied from 16 V to 47 V (the most typical range for low-molecular-weight compounds), and the focusing voltage offset was varied from 28 V to 79 V, which corresponds to a 75% offset from the minimum to the maximum focusing voltage modulo (Figure 7). The obtained data showed that the maximum peak area is achieved at 21 V focusing voltage paired with 37 V bias voltage (the ratio is 1.75). In this case, the peak area of the parent ion was 2905 ± 84 cnt × s. The minimum peak area was achieved at the boundary values of the studied range and were 2601 at 16 V and 2531 at 47 V (Figure 7). However, the signal CV barely differed from the optimum of the focusing voltage and was 3.5% and 3.9%, respectively. Nevertheless, it was decided to use a narrow range of focusing voltage within 19–24 V.

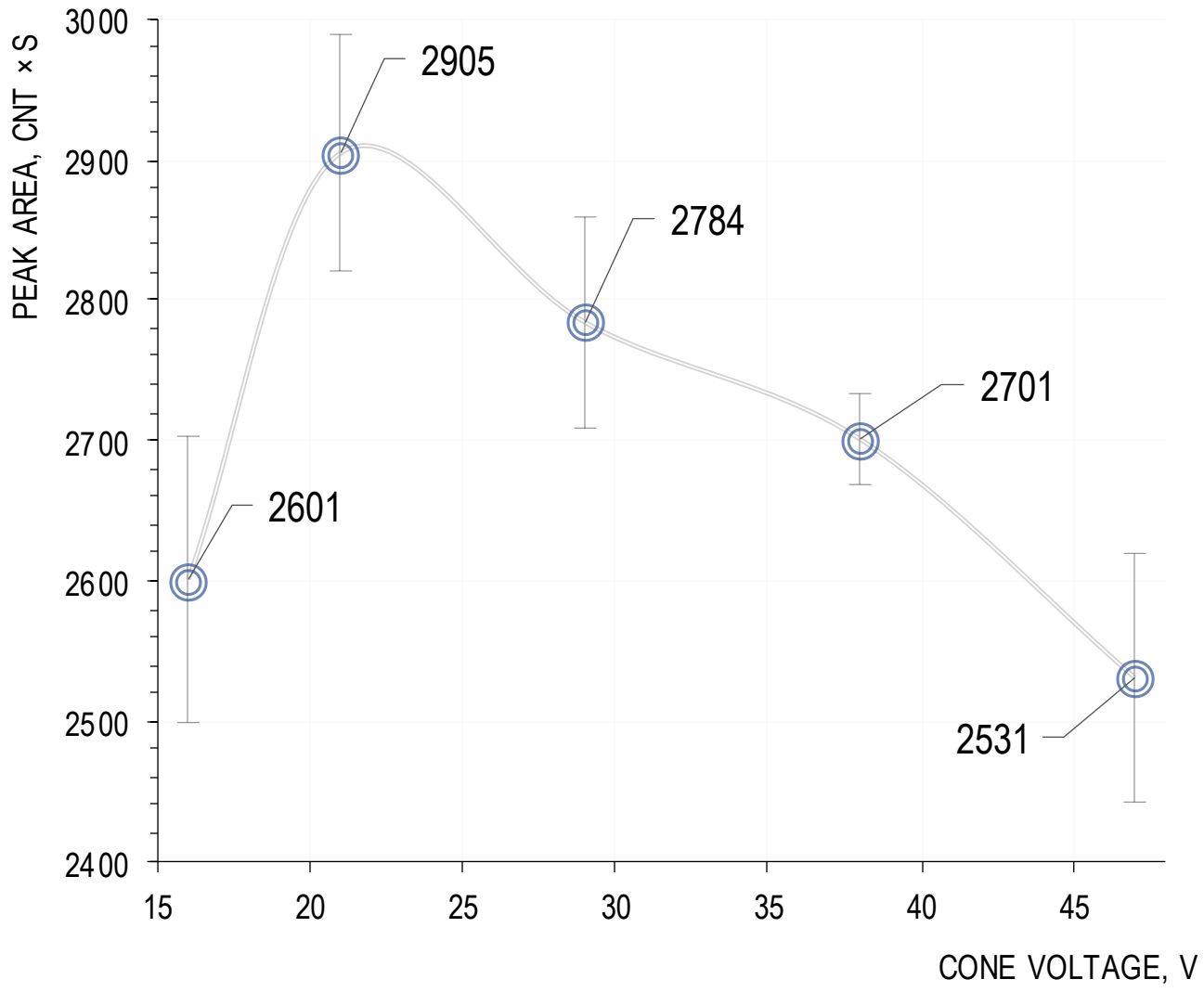

Figure 7. Dependence of the peak area of the parent ion m/z = 526.2262 (1+) of HAEE at a concentration of 4 ng/ml on the focusing voltage. The voltage shift by modulus is 75% for each set focusing voltage.

Next, we optimized the duty cycle time of a single complete scan, including the quadrupole switching delay time, the pulsar pulse time and the time spent on changing the alternating and direct current in the ion optics, is critical for the sensitivity[b]. Obviously, this time is spent mostly on the transmission of ions. Thus, the longer the time of one complete scanning cycle, the more ions can be passed through the optics, the greater the number of ions that overcome the distance of the time-of-flight tube and reach the detector in the form of an ion beam, which is reflected in the number of charges (ions) per unit of sampling time (cnt × s). However, at very large values of the time of one cycle, there is a risk of saturation of the detector and electron multiplier capacitance, which leads to a loss of sensitivity. First of all, this risk should be taken into account when analyzing high analyte concentrations or overloading the stationary phase of the chromatographic column with complex biological samples. On the other hand, short full scan cycle time allow for a large number of discrete ion accumulation cycles with low transmission ion, which can be convenient at high concentrations of the analyzed substance. However, this will affect the overall sensitivity to the analyzed substance, which will decrease. In the context of using chromatographic separation together with mass spectrometry, varying the full scan cycle time primarily affects the quality of the chromatographic peak: the shorter the cycle time, the more points can be placed on the envelope curve of the chromatographic peak, the larger the sampling step can be used to smooth it. However, the height and, accordingly, the area of such a peak will be small. Vice versa, the longer the scan time, the fewer points can be placed on the envelope curve, but the higher the height and area of such a chromatographic peak. Thus, when choosing the optimal values of the time of one complete scanning cycle, it is necessary to take into account the properties of the chromatographic stationary phase, the flow rate, and the chromatographic mobility of the substance being studied. In most cases, the time of one complete scanning cycle is selected empirically and is not a static value. To some extent, the Van Deemter equation can be used to determine the expected range of optimal values, which allows one to assume the theoretical full width of the chromatographic peak at the base at a given linear flow rate of the mobile phase:

$$H = 2\mu d_p + \frac{2\gamma D_G t_m}{L} + \frac{8k\, d_L^2 L}{D_L \pi^2 (1+k) t_m} \quad \text{(Formula 1)}$$

[b]The time spent by the ion optics and quadrupole ("dead time") typically varies within the range of up to 4.2 ms.

Where μ and d_p are the characteristics of the column packing unevenness and particle size, γ is the tortuosity of the flow channels of the system (typically taken as 1), D_G is the diffusion coefficient of the analyte in the mobile phase and depends on the physical properties of the mobile phase (in the case of gradient elution, not isocratic, it is necessary to take into account the viscosity of the resulting phase when mixing two or more mobile phases); these coefficients are reference values. L is the column length (in mm), and t_m is the retention time of the substance not bound to the stationary phase (under typical conditions, it can be calculated from the “dead” volume of the column, knowing its geometry and packing density (manufacturer's specification)); k is the retention factor, which can be calculated using Formula (1). DL and dL are the diffusion coefficient in the stationary phase and the average film thickness of the stationary phase, but under the conditions of using UPLC these values can be neglected.

Most of the terms can be extracted from the specifications of the chromatographic columns tested by the manufacturer, and some of the terms can be indirectly calculated from the column characteristics. In this case, at a flow rate of 0.3 ml/min (used in chromatographic separation), and taking into account the low longitudinal diffusion coefficient and mass transfer of HAEE standards on the stationary phase Acquity™ Premier HSS T3 (manufactured by Waters, Inc., column geometry 2.1 × 150 mm, particle size 1.8 μm, pore size 60 A, column serial number: SN 02443030115742, PN 186009469, LN 0244303011) [c], the average peak width at the base will be 0.12 min (about 7.2 seconds). For effective mathematical processing, it is necessary that the envelope of the chromatographic peak consist of at least six points, i.e. the maximum time of one full scanning cycle should not exceed 1200 ms. The lower limit is determined either arbitrarily or by a twofold excess of the threshold value of the background noise of the spectrum. However, the value of 1200 ms is very large, therefore, to improve the quality of the chromatographic peak, the density of the envelope curve points, we believe that the minimum number of points should be 15, then the upper limit of one full scanning cycle will be 480 ms.

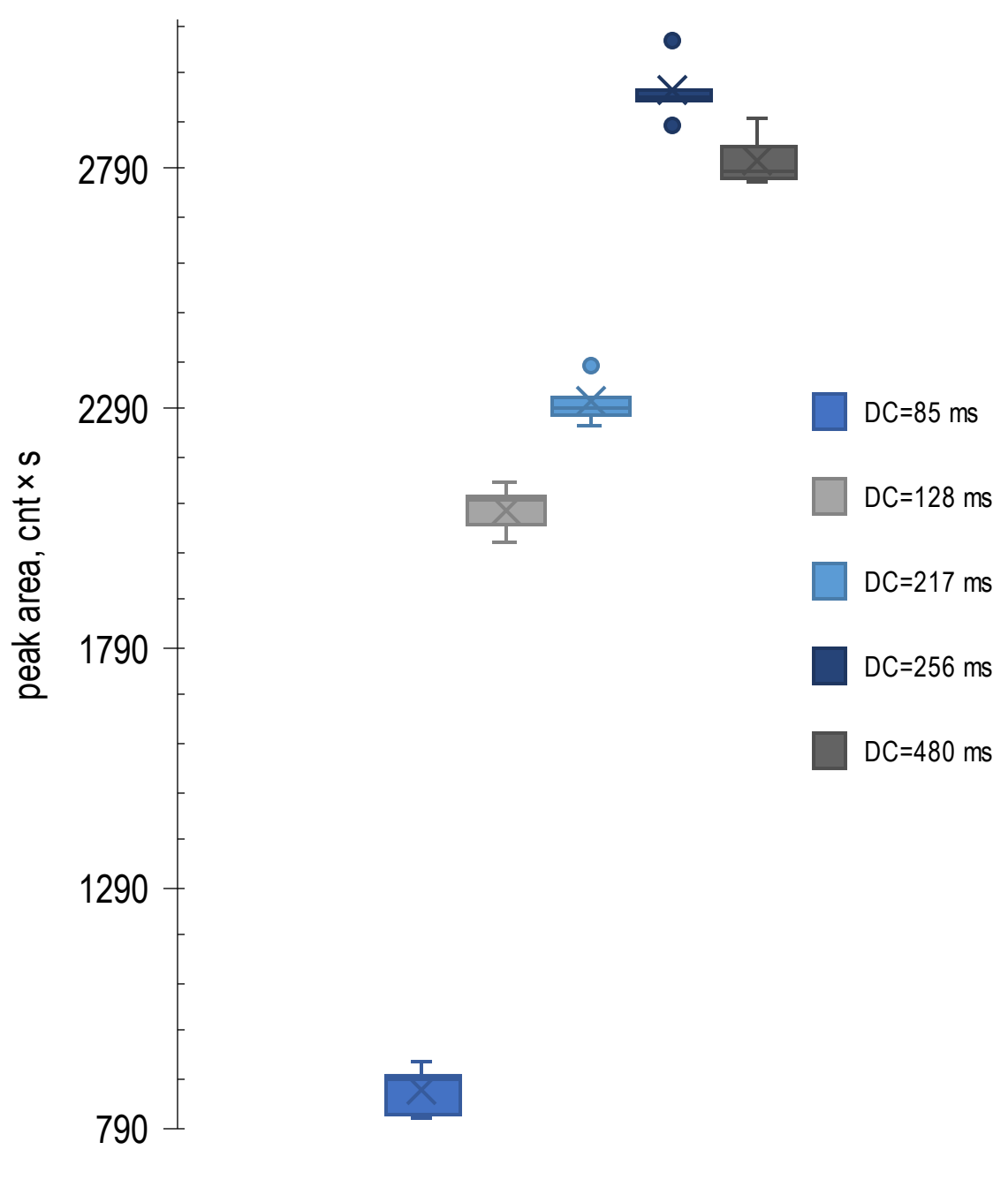

As a result, the following values of the full scanning cycle time were selected: 85 ms, 128 ms, 217 ms, 256 ms and 480 ms. The obtained data (**Figure 8**) confirm the expectations of the dependence of the chromatographic peak area (signal response) on the value of the full scanning cycle time. It can be noted that 256 ms is the optimal value of the full scanning cycle time, after which (480 ms) a smooth exit to a plateau (detector saturation) is observed.

Figure 8. Change in the area of the chromatographic peak of standard A1 (m/z = 526.2262 (1+)) depending on the time of one complete scanning cycle in the frontal mode. DC – duty cycle, ms

Well-characterized correlation is observed within the range of 85–256 ms: the minimum value of the scanning cycle time is characterized by the minimum average peak area (900±6.1% cnt × s), while the optimal value (256 ms) is three times greater than the minimum, and the peak area (response) also increases threefold (2871±1.8% cnt × s). The maximum time (480 ms) differs only by 1.2% from the optimal value (256 ms), but the absence of further dynamics in this range indicates a limitation of the detector capacity at a given upper concentration level (4 ng/ml). Considering that the average width of the chromatographic peak (empirical value) for standards of HAEE was $\omega_{(0.1)}$ = 6.4±0.18 sec, with a given optimal scanning cycle (256 ms), we can obtain up to 26 envelope points from the left to the right front of the chromatographic peak. In this case, the distribution of the point density can be expected to be homogeneous, since the peak asymmetry coefficient is As = 0.97±0.08, which also allows us to take into account the quantitative characteristics of the peak both at 10% of the height and at the base of the peak equally.

Based on the preliminary data on the HAEE structure (**Figure 3**), several fragment ions were selected for further analysis in the MRM (pseudo - SRM) mode and its optimization. Since the C-terminus of the peptide is represented by an amide group with low proton affinity, the registered fragment ions are represented by a sequence of N-terminal ions of the b- and a-type, completely covering the amino acid sequence of HAEE standards. **Table 1** presents the main characteristics of the transitions for the MRM method; the sequence of fragment ions is represented by the amino acid sequence of the *Ac* - HAE peptide [d], which, together with the mass of the parent ion, permits for the complete determination of the HAEE amino acid sequence with no doubts of its identity.

Table 1. Main characteristics of transitions for MRM (on a quadrupole time-of-flight mass spectrometer) recording of the non-labeled standard (m/z = 526.2256 (1+)) and heavy-labeled standard (m/z = 528.2329 (1+)).

Precursor ion, m/z	Fragment ion, m/z	Activation energy, eV	Ion type	Composition	Ion class
526.2256	180.0768	19	b1	*Ac* -H	quantifier
	152.0818	21	a1	*Ac* -H – [CO]	qualifier
	251.1139	29	b2	*Ac* -HA	

[c]Under formic acid mobile phase conditions, standards A1 and A2 will have a slight positive charge and will be more neutral for Acquity™ Premier HSS T3 chromatographic stationary phase.
[d]The b4 fragment ion represents the total mass of the precursor ion (*Ac* - HAEE - *Amide*) and corresponds to the mass of the precursor ion. Therefore, the b4 fragment is nominal and is absolutely identical to the precursor ion used as a source of transitions in the MRM mode.

Precursor ion, m/z	Fragment ion, m/z	Activation energy, eV	Ion type	Composition	Ion class
	380.1565	36	b3	*Ac* -HAE	
528.2329	182.0841	19	b1	[$^{13}C_2$] *Ac* -H	quantifier
	154.0893	21	a1	[$^{13}C_2$] *Ac* -H – [CO]	qualifier
	253.0855	29	b2	[$^{13}C_2$] *Ac* -HA	
	382.1641	36	b3	[$^{13}C_2$] *Ac* -HAE	

The registered a-type ion is also a diagnostic ion, since it forms a complementary pair with the b1-ion and is a fragment of the peptide at the C-Cα bond with neutral loss after induced decarboxylation. Traditionally, for quantitative analysis, the most intense ion (the base peak ion in the fragmentation spectrum) is selected as the quantifier ion, in relation to which the quantitative assessment of the content of the substance of interest is carried out. Other ions are represented by a collection of qualifier ions, the relative intensities and combination of which are necessary to confirm the identification of the substance (identity).

The maximum stability and yield of fragment ions were achieved with the activation energy ranging from 19 eV to 36 eV. In this case, as can be seen from the data in Table 1, the activation energy gradually increases with the size [e] (mass) of the ion. The activation energy for a particular type of ion are absolutely identical between labeled standard and its counterpart, since their chemical properties, structure and size are identical, and the difference in the isotopic composition of $^{12}C/^{13}C$ atoms in the terminal acetyl group does not affect the decomposition and the proton affinity (negative enthalpy) of the molecule. In addition, we note that the fragmentation spectrum also records an ion with m/z = 110.0718, which corresponds to the immonium salt of N -terminal histidine (*im* - H). However, this fragment ion is of little value for MRM analysis, since its m/z value is identical for both standards due to its formation after the decomposition of the N-terminal acetyl group containing the [^{13}C] isotopes. Thus, this ion is not included in the MRM registration, but it is a witness ion of the belonging of the registered molecule to the HAEE, regardless of the isotopic composition.

The discrete time of the complete scanning cycle was 256 ms per parent ion, per one complete scanning cycle. For the standards in equimolar concentration (5 ng/ml in an aqueous solution of formic acid), the reproducibility of the quantifier ion in ten technical replicates was by height H = 2.33 e 4±0.19 e 4 cnt (8.46%), by the area of the quantifier peak A = 7049±580 cnt × s (8.23%), the measurement accuracy was ε = 2.280±0.768 ppm.

[e] In the context of linear molecules with a homologous carbon skeleton, it is correct to identify size (physical linear dimensions) and mass (physical weight characteristics), since they correlate well with each other.

2.1. Chromatogram and Spectrum Properties

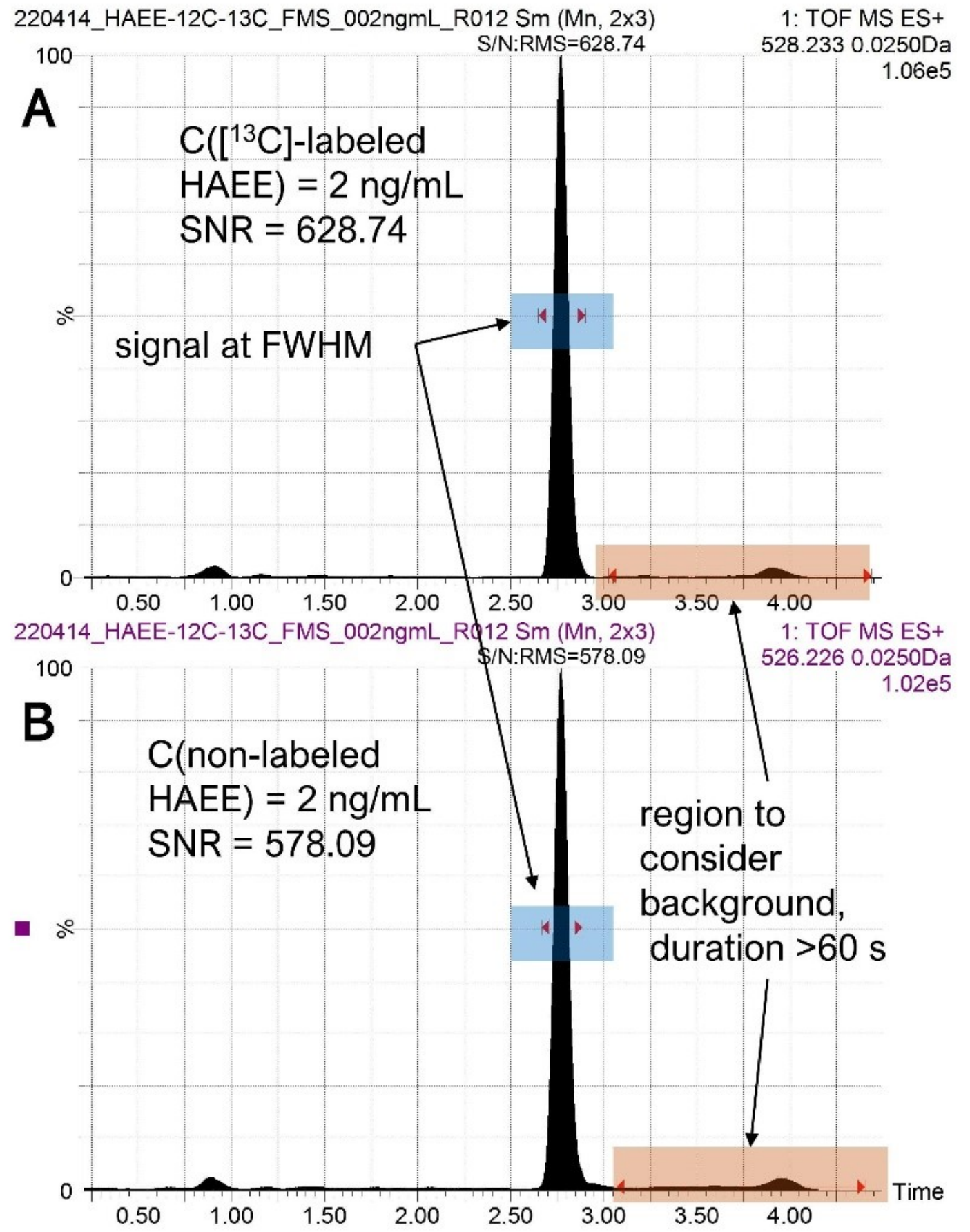

A typical chromatogram of non-labeled and labeled HAEE standards in full MS mode (**Figure 9**) display two chromatographic peaks identical in their mobility and correspond to m/z = 526.2256 (1+) and m/z = 528.2329 (1+). Using the example of an equimolar mixture of standards (the concentration of each standard is 2 ng/ml) in an aqueous solution of 0.5% formic acid (**Figure 9**), it is evident that the peaks coelute and their characteristic retention time on the column [f] is RT = 2.77±0.02 (0.72%) minutes, thus, non-labeled and labeled HAEE standards are characterized by a zero relative retention time (RRT = 0). The peak heights are related as RH = 1.041 (L/non-L ratio), the peak areas are related as RA = 1.052 (L/non/L ratio).

Figure 9. Extracted ion chromatogram of non-labeled and labeled HAEE standards in an equilibrium mixture (2 ng/mL of each standard in an aqueous solution of 0.5% formic acid). **(A)** Labeled HAEE standard with m/z = 528.2329 (1+), retention time on the column 2.77 minutes, signal-to-noise ratio SNR = 628. **(B)** Non-labeled labeled HAEE standard with m/z = 526.2256 (1+), retention time on the column 2.77 minutes, signal-to-noise ratio SNR = 578. The signal was calculated at the full width at half maximum (FWHM) of the chromatographic peak relative to the region of at least 60 seconds from the right front of the chromatographic peak.

The signal-to-noise ratio by standard deviation (RMS) is not less than SNR = 628 for labeled standard and not less than SNR = 578 for non-labeled HAEE standard. The mass measurement accuracy for labeled HAEE standard is ε = 0.874±0.221 ppm, for non-labeled HAEE standard it is ε = 0.613±0.289 ppm. The chromatographic peak is symmetrical (the asymmetry coefficient is As (0.1) = 0.978±0.057 [g]), the full width of the peak at the base is FW = 0.22 minutes (13.2 s), the width of the peak at half the maximum height is ω (0.5) = 0.07 (4.2 s),, the registration of non-labeled and labeled HAEE standards is carried out according to the four main transitions indicated abovein Table 1If the quantitative assessment is carried out according to the relative response of the base peak (quantifier ion, in this case the ion with m / z = 180 and m / z = 182 for non-labeled and labeled HAEE standards, respectively), then the identification, or identity of the registered molecule, in the MRM mode is traditionally assessed according to the set of other qualifier ions.

[f]Here and below, the data will be presented for chromatographic separation under optimized conditions on an Acquity™ Premier HSS T3 column manufactured by Waters, Inc., column geometry 2.1 × 150 mm, particle size 1.8 µm, pore size 60 A, column serial number: SN 02443030115742, PN 186009469, LN 0244303011

[g]The coefficient when measuring the half-width of the peak to the right and left fronts from the centroid at a level of 10% of the maximum height.

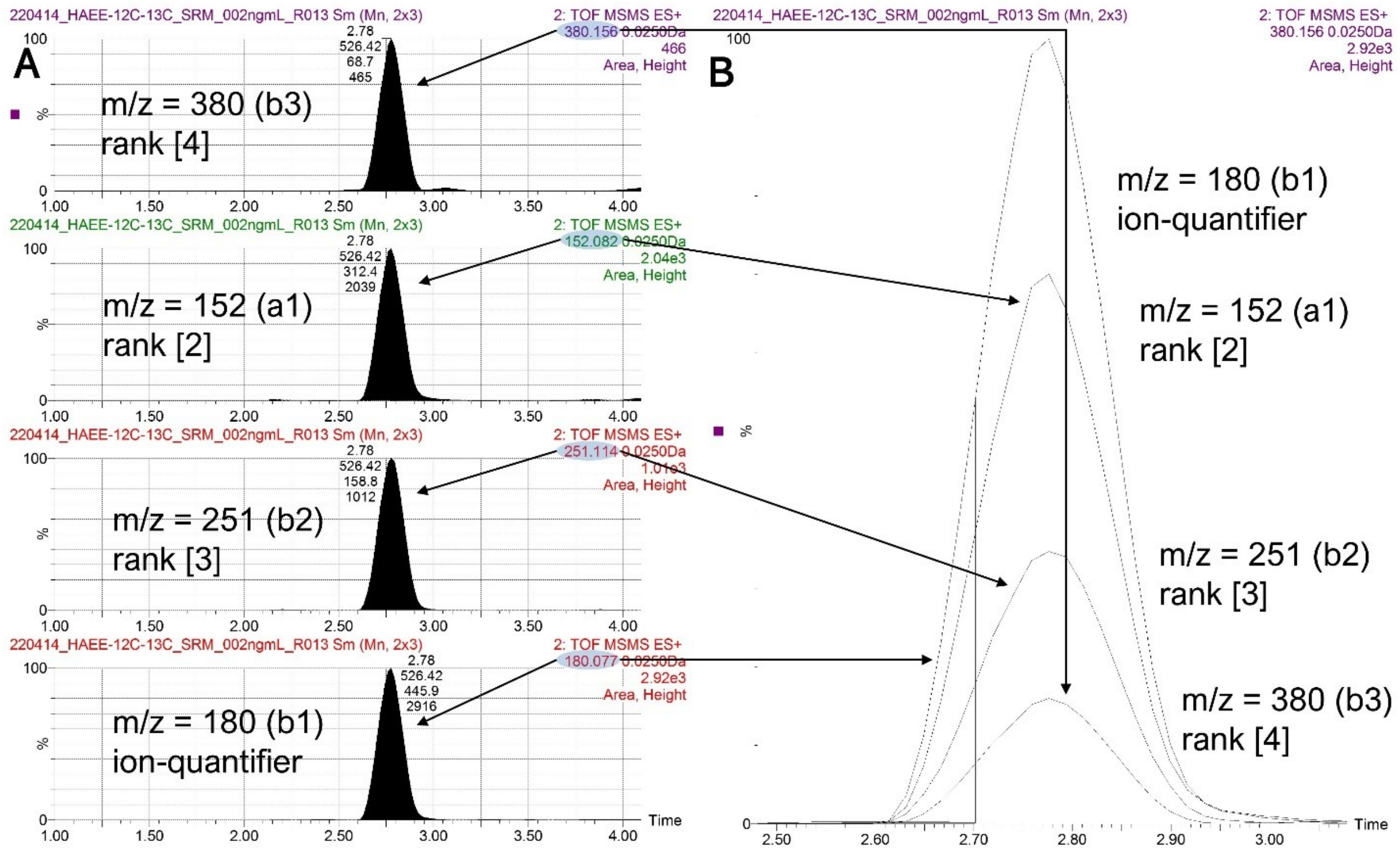

Figure 10. Extracted ion chromatogram in the MRM mode of non-labeled HAEE standard m/z = 526.2256 (1+), retention time on the column 2.78 in an equimolar mixture with labeled HAEE standard (2 ng/mL of each standard). **(A)** Deconvolution of the MRM signal over four transients corresponding to m / z = 380 (upper panel), m / z = 152, m / z = 251 and m / z = 180 (lower panel). The identity of the retention time of each fragment ion, i.e. their coelution, is an indicator of their origin from the same parent ion with m/z = 526.2256 (1+), of which they are transients. **(B)** Composite (consolidated) transient signal from standard A1 with m/z = 526.2256 (1+), obtained in MPM mode. Consolidation of inclusive peaks by the apex at the retention time on the column and by the peak width at the base corr esponds to the origin of the peaks from the same parent ion. The consolidated peak envelopes correspond to m/z = 180 (the uppermost peak, the base quantifier ion), then m/z = 152 (the second-ranked peak of the qualifier ion; see Table 2), then m/z = 251 (the third-ranked peak of the qualifier ion) and the lowest m/z = 380 (the fourth-ranked peak of the qualifier ion).

Figure 10 depicts the deconvoluted and overlaid chromatographic peaks corresponding to the signal of non-labeled standard with m/z = 526.2256 (1+) recorded in the MRM mode. The composite peak shows inclusive "nested" envelopes, each of which corresponds to a fragment peak. The height of the envelopes and the area under them are arranged in rank order. The upper peak (envelope) corr esponds to the peak of the quantifier ion (m/z = 180), the remaining peaks correspond to the qualifier ions, where the lowest peak is represented by the m/z = 380 ion. It is evident from the presented data that, as expected, the base peak is represented by a fragment with m / z = 180 (for labeled HAEE standard m/z = 182). This peak is a quantifier ion and for this reason is taken as 100% by the peak height and area, which are used for quantitative assessment. The areas and heights of other qualifier ions, including the diagnostic ion of the a 1-type, are normalized, respectively, to the area and height of the base peak.

Table 2. Main characteristics of the signal stability of non-labeled and labeled HAEE standards in an equimolar mixture (2 ng/ml of each standard) in the MRM scanning mode. The relative response was calculated as the ratio of the [^{13}C] standard to the [^{12}C] standard [h].

Rank	Fragment, m/z	Peak area, cnt × s	Peak height, cnt	Relative area	Relative height	Discrete accumulation, s	Relative area response ($^{13}C/^{12}C$)	Relative Altitude Response ($^{13}C/^{12}C$)
1	180	446	2916	100.00%	100.00%	100.00%	1.018	1.007
3	251	159	1012	35.60%	34.70%	102.60%		
2	152	312	2039	70.10%	69.90%	100.20%	1.051	1.023
4	380	69	465	15.40%	15.90%	96.60%		
1	182	438	2897	100.00%	100.00%	100.00%	1.041	1.026
3	253	151	989	34.50%	34.10%	101.00%		
2	154	300	1987	68.50%	68.60%	99.90%	0.954	0.985
4	382	72	472	16.40%	16.30%	100.90%		

According to the rank arrangement, the second ion (qualifier ion) is the peak with m/z = 152 (m/z = 154), for which signal fluctuations of up to 20% in absolute value are acceptable. That is, in this case, both the height and area of the peak are 70% of the base (see above Table 2), this means that the permissible limits of signal fluctuation are from 56% to 84% (20% of 70% in absolute value is 14%) of the relative area or height to the base quantifier ion. For the third highest qualifier ion (m/z = 251 for non-labeled HAEE standard and m/z = 253 for labeled HAEE standard), the relative height and area are 35%, and the limits of permissible signal fluctuation are 10% on a relative scale.

[h] Any order of calculation of direct and reciprocal quotient is allowed; the main condition is the correspondence of the calcul ated value to the expected one. In this case, direct and reciprocal quotient will be identical, since the mixture is equimolar. In the case of a nonequilibrium mixture, the value of direct and reciprocal quotient should differ by the value of the dilution coefficient of the mixture components in relation to each othe r.

That is, the limits of signal fluctuation are from 25% to 45% in relation to the base peak. For the fourth and subsequent (if any) ions, the permissible fluctuations are up to 15% if its initial intensity is at least 20% of the base ion, and up to 25% if its initial intensity is less than 15% of the base peak.

2.2. Signal Significance Threshold

The determination of the signal significance threshold in pilot experiments was carried out by analyzing the interference signal in a blank sample (solvent), in a biological sample (matrix interference) and by the detection limit in a surrogate matrix.

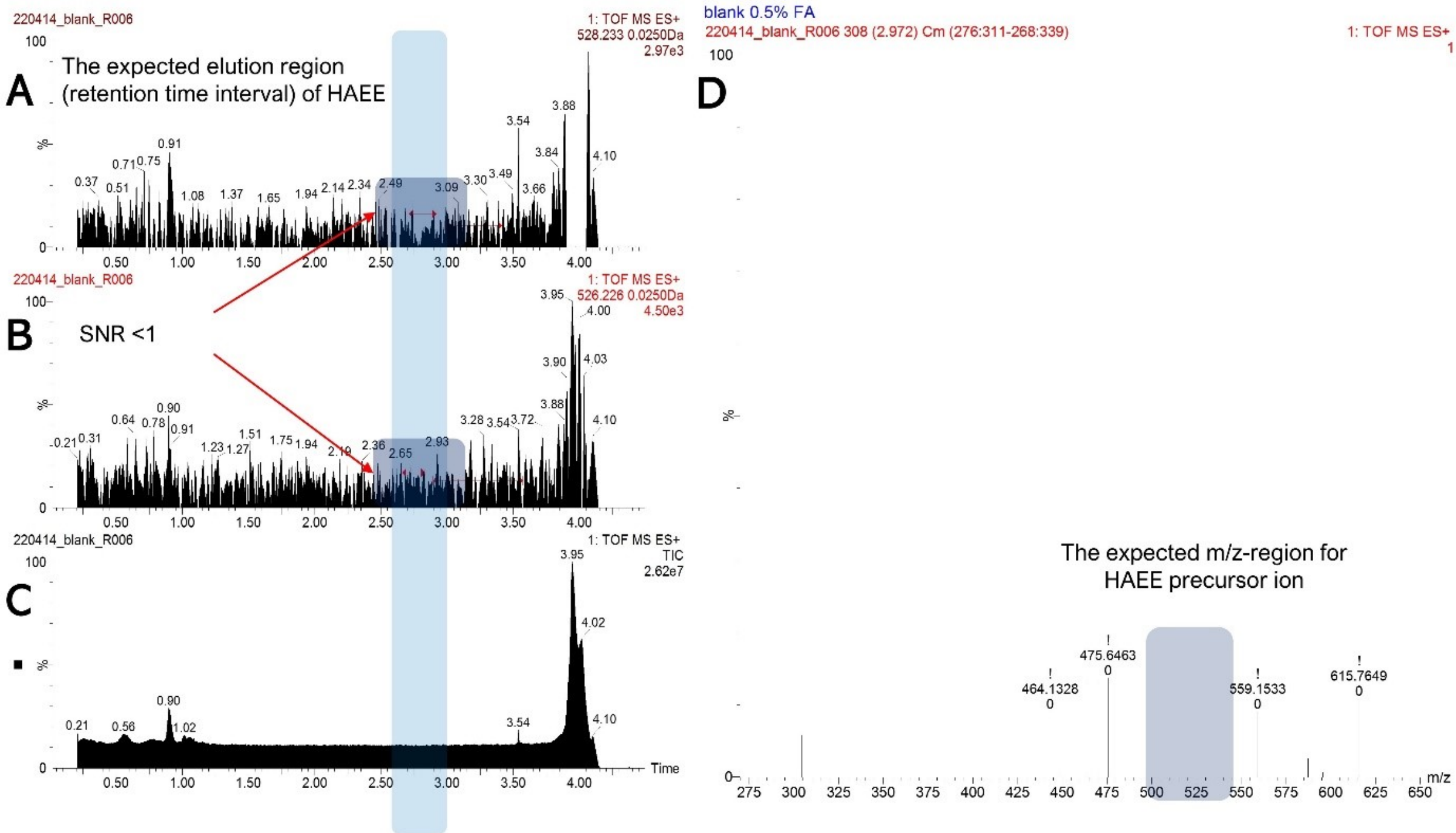

Figure 11. Isolated ion current chromatogram for (A) labeled HAEE with m/z = 528.2329 (1+) and (B) non-labeled HAEE standard with m/z = 526.2256 (1+) in a blank sample (system blank: solvent is an aqueous solution of 0.5% formic acid). The signal-to-noise level is less than unity (SNR < 1) in both cases. (C) Total ion current chromatogram in a blank sample. Peaks corresponding to standards in the characteristic retention time range on the column specified for them are not detected. (D) Frontal spectrum in the characteristic retention time range on the column for standards A1 and A2. The intensity of the spectrum on an absolute scale does not reach unity; the median intensity value is 0.27 cnts. Peaks corresponding to the mass-charge values of standards were not detected.

Figure 11 demonstrates that the isolated ion current chromatograms do not allow differentiating the signal corresponding to the identification criteria of both standards in a blank sample. The signal-to-noise level (indicated by the horizontal red interval in Figure 11) is SNR = 0.78 for non-labeled standard and SNR = 0.51 for labeled HAEE standard (average values based on the results of multiple measurements; n > 10). The chromatographic peaks are not formed; the morphology of the envelope curves is not expressed. In terms of the signal-to-noise level, the signal in the specified retention time interval characteristic of both standards does not differ from the background signal. The median signal level for BPC for non-labeled standard is 712±488 cnts, for labeled HAEE standard 916±356 cnts. The frontal scanning spectrum (Figure 11) is represented by a poor spectrum with scattered peaks, for which the maximum intensity level on the absolute scale does not reach even one. The median signal level in the frontal spectrum is 0.27 cnts. Thus, the possibility of potential interference of the signal of both standards in blank samples is not considered.

Determining the threshold significance of non-labeled HAEE standard signal in a biological sample (plasma or blood serum) is not possible, since non-labeled HAEE is an endogenous molecule, i.e., it is an integral property of the biological sample itself and the biological system. Thus, non-labeled HAEE is always present in a biological sample initially within the undefined boundaries of the concentration range (Figure 12).

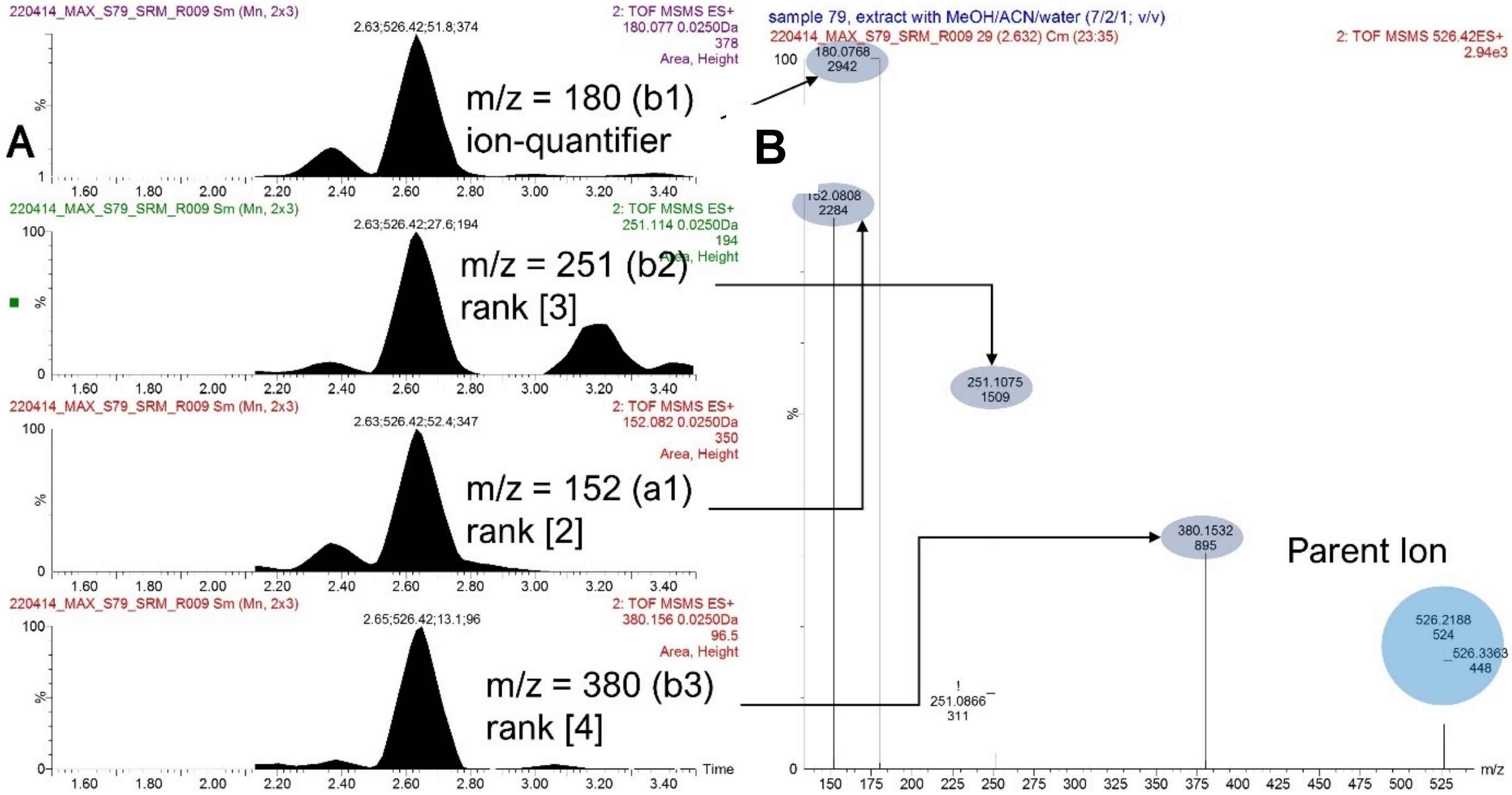

Figure 12. Deconvoluted MRM signal of endogenous substance (A) corresponding to non-labeled HAEE standard with amino acid sequence Ac - HAEE - Amide in biological sample number 79 (human blood plasma; after double freeze-thaw cycle) after isolation on an anion-exchange carrier under optimized conditions of solid-phase extraction (SPE) and optimized conditions of chromatographic separation. Deconvoluted chromatogram of MRM signal of endogenous HAEE in biological sample is identical to signal of standard (using data in Figure 10). (B) MRM fragmentation spectrum of endogenous HAEE corresponding to standard of HAEE by set of fragment ions, as well as their ranking by relative intensity (see Table 2).

Thus, determination of the significance of the signal is possible only in the presence of the internal labeled HAEE standard, identical in spectral, physicochemical and chromatographic properties to the non-labeled HAEE standard, which is an endogenous element of a human biological sample.

Section 3. Calibration Curve

The calibration curve [i] was plotted by the peak area and by the height against the concentration levels within the calibration range. from 0.01 ng/ml (10 pg/ml) to 10 ng/ml, i.e. within three orders of magnitude of the dynamic concentration range. In each of the n ine calibration levels, the concentration of the internal standard (ISTD), i.e. labeled HAEE standard with [^{13}C] atoms, was constant and amounted to 1 ng/ml. The analyte concentration (non-labeled HAEE standard) was varied and was presented by the following values (in descending order): 10 ng/mL, 5 ng/mL, 2.5 ng/mL, 1 ng/mL, 0.5 ng/mL, 0.25 ng/mL, 0.1 ng/mL (100 pg/mL), 0.05 ng/mL (50 pg/mL), 0.025 pg/mL (25 pg/mL) and 0.01 ng/mL (10 pg/mL) [j]. At each calibration level, measurements were carried out in seven consecutive technical replicates without excluding any of the replicates for further calculations. Thus, the scheme for constructing a calibration curve with an internal standard and measuring the analyte concentration by the relative response was realized. This algorithm also allows tracking the stability of the calibration curve over large concentration ranges (three or more orders of concentration) and applying the necessary weighting factors to correct the calibration curve in its various sections. Table 3 shows the summary data of measurements at each calibration level in each technical replicate. The first column shows the concentration levels according to non-labeled HAEE standard starting from 10 ng/ml to 25 pg/ml [k], the concentration according to standard A2 is not shown, since it is constant throughout the entire measuring range and is 1 ng/ml.

Table 3. Summary of the calibration curve of the analyte (correspond to non-labeled HAEE standard) in the concentration range from 0.025 ng/mL (25 pg/mL) to 10 ng/mL using the internal standard (labeled HAEE standard) with a constant concentration of 1 ng/mL throughout the calibration curve. Each calibration level was measured in seven consecutive technical replicates. Data for each level and for each technical replicate are shown. **[12C], ng/mL** is the concentration of the analyte; **[12C] A, cnt × s** is the area of the chromatographic peak of the analyte in the full MS scan mode; **[13C] A, cnt × s** is the area of the chromatographic peak of the internal standard; **[12C] H, cnt** is the height of the chromatographic peak of the analyte; **[13C] H, cnt** is the height of the chromatographic peak of the internal standard; **[12C]/[13C] RR [A]** – relative response (of the analyte to the internal standard) by the area of the chromatographic peak; **[12C]/[13C] RR [H]** – relative response by the height of the chromatographic peak; **δ/δ⁰, A** – accuracy of the measured concentration to the expected one by the area of the chromatographic peak, **δ/δ⁰; H** – accuracy of the measured concentration to the expected one by the height of the chromatographic peak; STD [12C] A – standard deviation of the area of the chromatographic peak of the internal standard; **STD [12C] H** – standard deviation of the height of the chromatographic peak of the analyte; STD [13C] H – standard deviation of the height of the chromatographic peak of the internal standard.

analyte concentration (non-labeled HAEE standard) [12C], ng/mL	area of the chromatographic peak of the analyte (non-labeled HAEE) [12C] A, cnt × s	chromatographic peak area of the internal standard (labeled HAEE standard) [13C] A, cnt × s	height of the chromatographic peak of the analyte [12C] H, cnt	height of the chromatographic peak of the internal standard [13C] H, cnt	relative response (analyte to internal standard) by chromatographic peak area [12C]/[13C] RR [A]	relative response to chromatographic peak height [12C]/[13C] RR [H]	accuracy of the measured concentration to the expected one based on the area of the chromatographic peak δ / δ⁰, A	accuracy of the measured concentration to the expected one based on the height of the chromatographic peak δ / δ⁰, H	standard deviation of the chromatographic peak area of the analyte STD [12C] A	standard deviation of the chromatographic peak area of the internal standard STD [13C] A	standard deviation of the chromatographic peak height of the analyte STD [12C] H	standard deviation of the chromatographic peak height of the internal standard STD [13C] H	Interlevel response of analyte (tends to the interlevel dilution coefficient)	Inter-level response of internal standard (tends to unity)
10	56949	7216	560432	74644	7.892	7.508	21.08%	24.92%	1.57%	2.12%	3.30%	2.43%	-	-
10	55859	7573	569699	78016	7.376	7.302	26.24%	26.98%						
10	56144	7692	559857	78003	7.299	7.177	27.01%	28.23%						
10	56146	7583	564422	75963	7.404	7.43	25.96%	25.70%						
10	54214	7639	519773	72958	7.097	7.124	29.03%	28.76%						
10	55555	7438	554088	74699	7.469	7.418	25.31%	25.82%						
10	55066	7583	533392	75775	7.262	7.039	27.38%	29.61%						
5	27597	6249	265067	60598	4.417	4.374	11.67%	12.52%	1.44%	4.04%	2.77%	5.54%	2.036	1.196
5	26642	5999	257671	58922	4.441	4.373	11.18%	12.54%						
5	27304	6532	267868	63318	4.18	4.231	16.40%	15.39%						
5	27725	6544	271557	65876	4.237	4.122	15.26%	17.56%						
5	27702	6578	274712	66528	4.211	4.129	15.77%	17.41%						
5	27056	6009	255732	57280	4.503	4.465	9.95%	10.71%						
5	27484	6161	272535	62558	4.461	4.357	10.78%	12.87%						
2.5	13755	5503	132957	53250	2.499	2.497	0.03%	0.13%	2.23%	2.14%	2.77%	2.70%	2.026	1.129
2.5	13378	5580	126732	53962	2.397	2.349	4.11%	6.06%						
2.5	13690	5602	134004	54329	2.444	2.467	2.24%	1.34%						
2.5	13595	5762	131746	55029	2.359	2.394	5.63%	4.24%						
2.5	13613	5676	132726	56489	2.398	2.35	4.07%	6.02%						
2.5	12878	5399	124440	52318	2.385	2.379	4.60%	4.86%						
2.5	13635	5517	132324	55939	2.471	2.366	1.14%	5.38%						
1	5045	5102	47757	48430	0.989	0.986	1.12%	1.39%	3.76%	4.17%	4.76%	5.18%	2.536	1.069
1	5312	5359	51178	52784	0.991	0.97	0.87%	3.04%						
1	5363	5295	53355	52297	1.013	1.02	-1.28%	-2.02%						
1	5262	5233	51631	51518	1.006	1.002	-0.57%	-0.22%						
1	5716	5278	55976	52200	1.083	1.072	-8.30%	-7.23%						
1	5312	5471	51648	53452	0.971	0.966	2.91%	3.37%						
1	5265	4798	51818	46240	1.097	1.121	-9.73%	-12.06%						
0.5	2506	5104	24653	50684	0.491	0.486	1.81%	2.72%	2.25%	2.98%	1.57%	2.23%	2.068	0.993
0.5	2591	5303	25309	51464	0.489	0.492	2.28%	1.64%						
0.5	2663	4972	25551	50232	0.536	0.509	-7.12%	-1.73%						
0.5	2632	5361	25541	52792	0.491	0.484	1.82%	3.24%						
0.5	2515	5314	24906	53174	0.473	0.468	5.37%	6.32%						
0.5	2558	5369	25428	52889	0.476	0.481	4.71%	3.84%						
0.5	2561	5375	25783	52347	0.476	0.493	4.70%	1.49%						
0.25	1323	4839	12731	47075	0.273	0.27	-9.35%	-8.18%	6.80%	3.67%	7.70%	3.83%	2.017	1.045
0.25	1450	4823	12521	46437	0.301	0.27	-20.28%	-7.85%						
0.25	1208	5196	10252	50083	0.232	0.205	7.01%	18.12%						
0.25	1215	5219	12123	51459	0.233	0.236	6.89%	5.77%						
0.25	1247	4844	11106	47874	0.257	0.232	-2.98%	7.21%						

[i] To visualize the distribution of the point density in the correlation field, the axes of the peak area, peak height, and concentration levels were converted to a logarithmic scale with a base of "2" (the base of the logarithm was chosen based on the standard dilution scheme – 1:2).

[j] Reference information for navigation through the concentration values of labeled and non-labeled HAEE standards:

Absolute concentration	Molar concentration	Degree of molarity	Absolute load	Quantitative load	Colloidal concentration
525 mg/ml	1 M	e0	-	-	-
100 mg/ml	190.48 mm	e-1	-	-	-
1 mg/ml	1.905 mM	e-3	-	-	-
1mcg/ml	1.905 µM	e-6	-	-	1 ppm
1 ng/ml	1.905 nM	e-9	8 pg	9.525 femtomoles	1 ppb
5 ng/ml	9.525 nM	e-9	40 pg	76.2 femtomoles	5 ppb
50 pg/ml	95.25 pm	e-11	400 fg	476.25 attomoles	50 ppt (500 ppq)
25 pg/ml	47.63 pm	e-11	200 fg	238.13 attomoles	25 ppt (25 ppq)

[k] The concentration of 10 pg/mL was excluded from the data table due to the lack of a stable relevant signal at this calibration level.

analyte concentration (non-labeled HAEE standard) [12C], ng/mL	area of the chromatographic peak of the analyte (non-labeled HAEE) [12C] A, cnt × s	chromatographic peak area of the internal standard (labeled HAEE standard) [13C] A, cnt × s	height of the chromatographic peak of the analyte [12C] H, cnt	height of the chromatographic peak of the internal standard [13C] H, cnt	relative response (analyte to internal standard) by chromatographic peak area [12C]/[13C] RR [A]	relative response to chromatographic peak height [12C]/[13C] RR [H]	accuracy of the measured concentration to the expected one based on the area of the chromatographic peak δ / δ^0, A	accuracy of the measured concentration to the expected one based on the height of the chromatographic peak δ / δ^0, H	standard deviation of the chromatographic peak area of the analyte STD [12C] A	standard deviation of the chromatographic peak area of the internal standard STD [13C] A	standard deviation of the chromatographic peak height of the analyte STD [12C] H	standard deviation of the chromatographic peak height of the internal standard STD [13C] H	Interlevel response of analyte (tends to the interlevel dilution coefficient)	Inter-level response of internal standard (tends to unity)
0.25	1216	5174	11746	50174	0.235	0.234	5.98%	6.36%						
0.25	1279	5110	12623	49975	0.25	0.253	-0.10%	-1.03%						
0.1	427	5098	3824	48608	0.084	0.079	16.28%	21.33%						
0.1	547	4903	5236	48604	0.111	0.108	-11.48%	-7.73%						
0.1	548	5123	5138	49453	0.107	0.104	-6.92%	-3.90%						
0.1	586	5139	5212	50174	0.114	0.104	-14.10%	-3.88%	9.52%	1.98%	10.46%	2.33%	2.392	0.995
0.1	563	5036	5292	50553	0.112	0.105	-11.87%	-4.68%						
0.1	528	5155	5155	51907	0.102	0.099	-2.48%	0.69%						
0.1	537	4940	5178	50150	0.109	0.103	-8.79%	-3.25%						
0.05	248	4901	2490	49260	0.051	0.051	-1.37%	-1.10%						
0.05	227	4873	2559	47608	0.047	0.054	7.00%	-7.50%						
0.05	251	4942	2096	50083	0.051	0.042	-1.62%	16.30%						
0.05	311	4809	2654	48047	0.065	0.055	-29.38%	-10.48%	14.13%	2.08%	8.83%	2.72%	1.957	1.024
0.05	282	5067	2251	51081	0.056	0.044	-11.36%	11.87%						
0.05	335	5083	2702	50773	0.066	0.053	-31.70%	-6.43%						
0.05	255	4880	2485	48624	0.052	0.051	-4.46%	-2.21%						
0.025	133	5227	1051	53139	0.025	0.02	-1.55%	20.89%						
0.025	121	4911	1212	50651	0.025	0.024	1.77%	4.29%						
0.025	117	5066	1093	51721	0.023	0.021	7.77%	15.47%						
0.025	125	5282	1133	52817	0.024	0.021	5.50%	14.19%	8.09%	2.38%	6.57%	2.24%	2.157	0.968
0.025	146	5073	1061	51074	0.029	0.021	-15.20%	16.90%						
0.025	126	5077	1021	50188	0.025	0.02	0.57%	18.63%						
0.025	118	5071	1007	50554	0.023	0.02	7.00%	20.32%						

The correctness of the analyte dilutions with the internal standard can be assessed by the value of the ratio of the area or height of the chromatographic peak for the analyte between adjacent calibration levels. This value should tend to the degree of dilution between adjacent or corresponding calibration levels. For example, the degree of dilution between 1 ng/mL and 0.5 ng/mL is d = 2, and the interlevel response of the analyte is 2.068 (Table 3), between calibration levels of 0.25 ng/mL and 0.1 ng/mL the dilution factor is d = 2.5, while the interlevel response is 2.392 (Table 3). The closer the correspondence of the interlevel response to the expected value, the more correct the dilutions and the greater the predictability of the calibration curve behavior. If, throughout the entire concentration range, the maximum correspondence of the interlevel response to the value of the dilution coefficient between adjacent calibration levels is observed, there is a high probability of a linear dependence of the signal. Meanwhile, the interlevel response for the internal standard should always be constant at each calibration level, regardless of the analyte concentration, since the concentration of the internal standard is constant throughout the entire range under study and is 1 ng/ml. We can track this relationship by the value of the interlevel response of the standard in Table 3, which really tends to unity at each level. However, in the upper concentration range, a slight excess of up to 1.196 and 1.129 is observed, respectively, at 10 ng/ml and 5 ng/ml. Further, minor fluctuations in the signal and its stabilization within 0.968–1.069 are observed, which fully corresponds to expectations. I assume that the excess of the relative response by 13–20% in the upper range is due to the effect of isotope interference.

When obtaining the basic fragmentation spectrum, it was found that the use of standards in an equilibrium mixture with high concentrations (10 ng/mL) reveals a small interference. Against the background of the basic spectrum, it was estimated that at these concentrations the isotope effect is small and can be (estimated) 0.59 ppb at a concentration of 10 ng/mL of non-labeled HAEE standard A1 (the contribution of its third isotope to the intensity of the first isotope of labeled HAEE standard). Thus, the preliminary estimate of the contribution of isotope interference at the level of obtaining the basic full spectrum was underestimated, and the total contribution at the upper limits of the concentration dependence can be from 12% to 20%, however, when measuring concentrations below 5 ng/mL (Table 3) this contribution becomes significantly lower and in the equilibrium mixture (calibration level number four: 1 ng of standard and 1 ng/ml of analyte) is no more than 6.9% or 0.69 ppb when normalized to a concentration of 10 ng/ml, which is a very close value to the primary estimate (0.59 ppb) when obtaining the basic full spectrum.

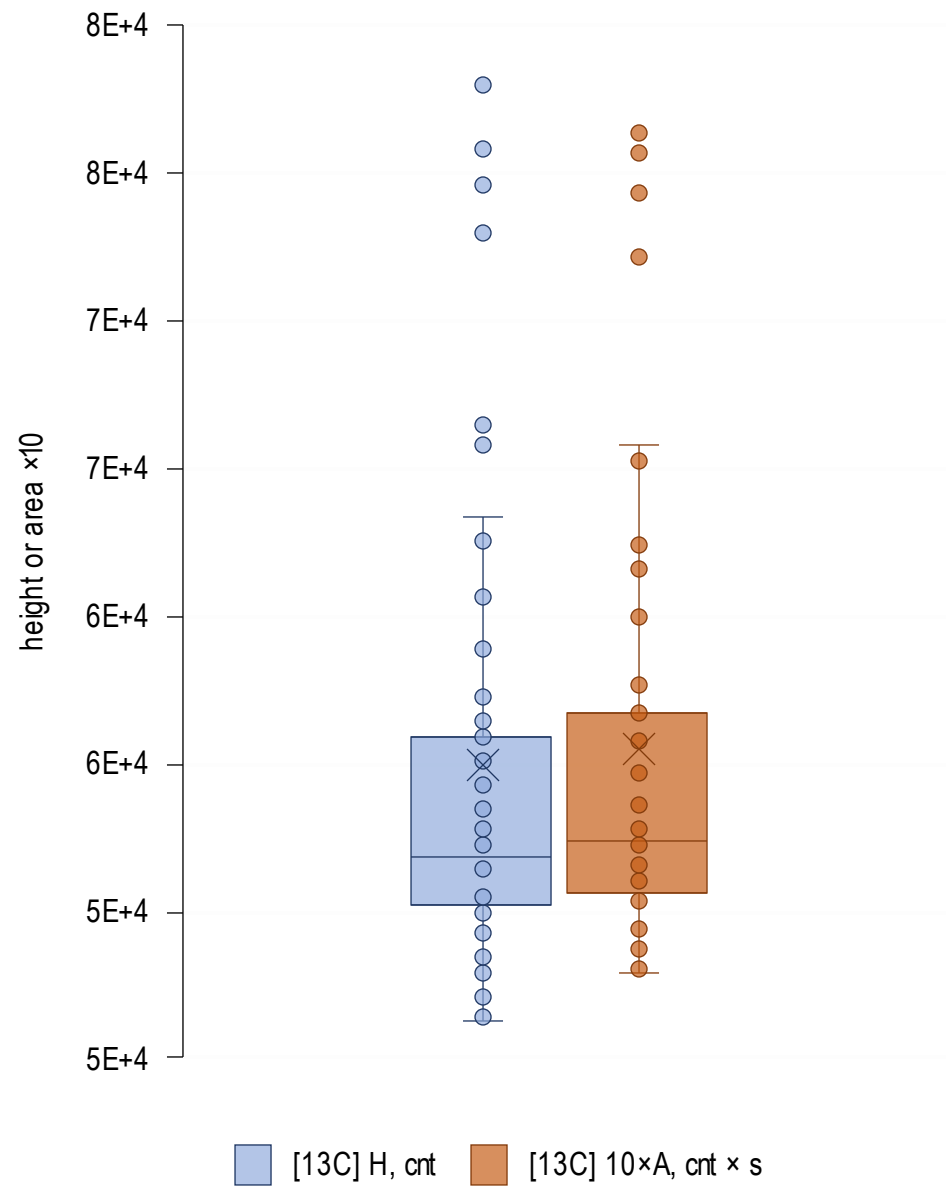

Figure 13. Distribution of the measurement density of the internal standard at a concentration of 1 ng/ml over the entire concentration range of the calibration curve. A multiplier of ×10 was applied to the area of the chromatographic peak to align it with the axis of the chromatographic peak height values. The outliers of values beyond the first quartile relate to the upper concentration limits of the calibration curve (10 ng/ml and 5 ng/ml) and are due to the effect of isotope interference.

Figure 13 shows a concentration of 1 ng/mL over the entire range of the calibration curve. A multiplier (×10) was applied to the peak area value to align the remaining values with the peak height (see the original data in Table 3). The full linear range of values covers the range from 4.6e5 (cnt/cnt × s) to 7.8e5 (cnt/cnt × s), with the upper measurement points corresponding exactly to the upper limits of the concentration range of the calibration curve. The median (5.2e5 cnt/cnt × s) and the mean value (5.5e5 cnt/cnt × s) coincide fairly well, indicating the similarity of the density of measurements in the main range of the calibration curve to a normal distribution. Excluding the upper concentration limits (5 ng/mL and 10 ng/mL) from consideration reduces the median and mean values to a single value (5.06e5 cnt/cnt × s), and the linear range of the residual range of values is reduced to 18% of the difference between the minimum and maximum data series.

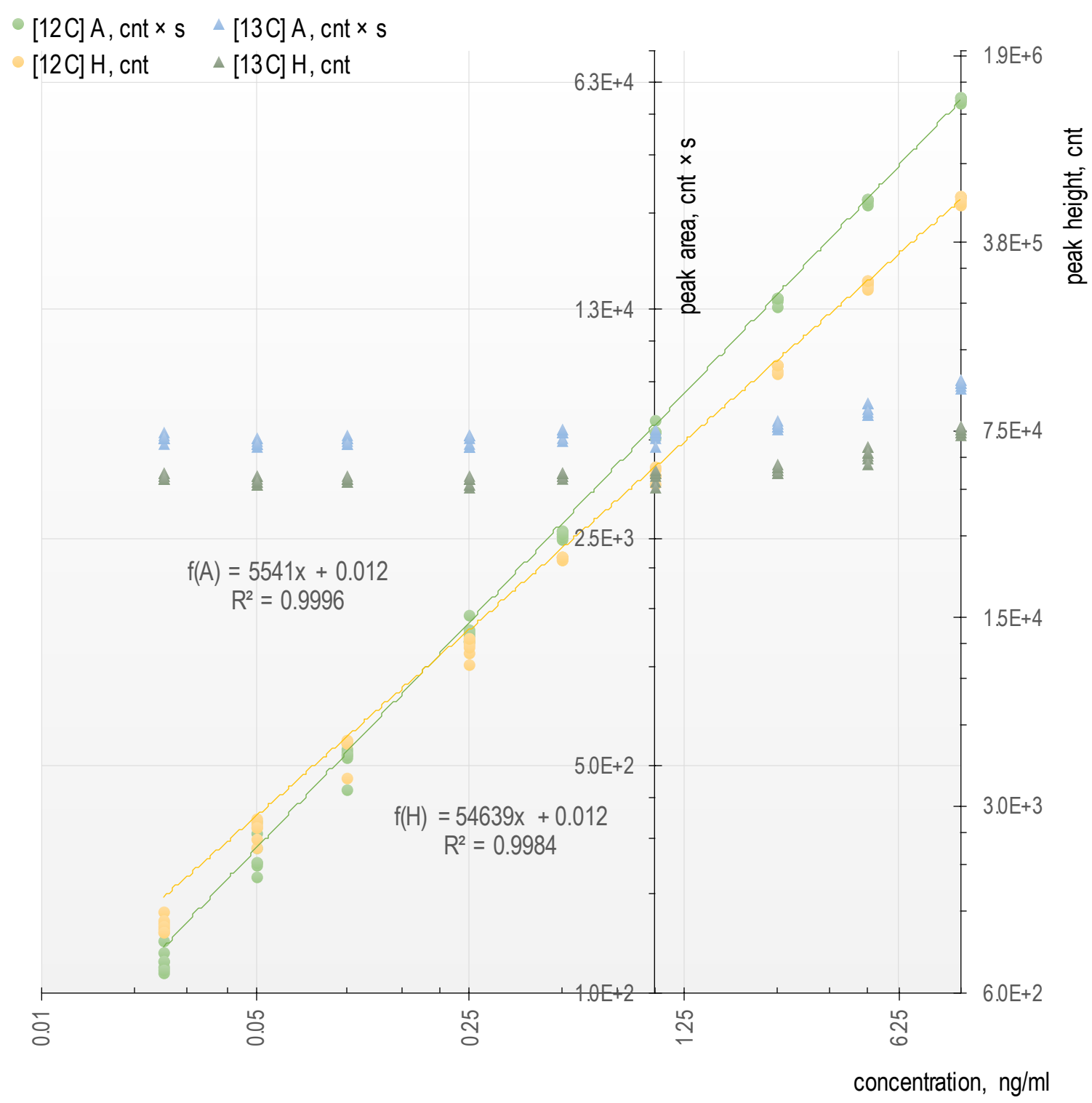

Figure 14. Calibration curve for the analyte (non-labeled HAEE standard) within the concentration range from 0.025 ng/mL (25 pg/mL) to 10 ng/mL. The calibration curve constructed from the relative response of the chromatographic peak area is shown in blue; the calibration curve constructed from the relative response of the chromatographic peak height is shown in green. The internal standard with stable isotopes of the [^{13}C] carbon atom at a constant concentration of 1 ng/mL throughout the entire range of the calibration curve is shown by the upper row of points by peak height and the lower row of points by peak area. The point of intersection of the calibration curves with the internal standard lies at 1 ng/mL, corresponding to the equilibrium mixture of the components of the fixanal of the fourth calibration level. The area and height scale of the chromatographic peak, as well as the concentration scale are logarithmic (with the base log (5)) to display the density of points in the region of low concentrations.

The calibration curve was constructed throughout the entire studied concentration range except for the last level (0.01 ng/ml = 10 pg/ml), since at this level it was not possible to discriminate a signal that met the identification criteria and exceeded the background signal by at least two times in terms of signal-to-noise level. Calibration curves (Figure 14) were constructed by the area and height of the chromatographic peak to compare the signal reproducibility and the accuracy of measuring the empirical concentration (correspondence to the calculated expected concentration). As can be seen from the data presented (Figure 14 and Table 3), the calibration curve constructed both by the peak area and by the peak height fully satisfies the linearity conditions. For both parameters (height (H) and area (A)) the slope tangent is practically identical, which is expressed in close values of the determination coefficients (R^2 = 0.9996 and R^2 = 0.9984 for the curve by area and by peak height, respectively). The use of the relative response (RR) for the internal standard with stable isotopes [^{13}C] of the carbon atom allows smoothing out possible signal fluctuations in areas with high and, in particular, low concentrations of the analyte (non-labeled HAEE standard). In particular, at the lower level of the calibration curve at the concentration point of 0.025 ng/ml (25 pg/ml), the signal covariance is only 8.09% and 6.57% by area and by height of the chromatographic peak, respectively, taking into account all seven technical repetitions. In general, the median relative standard deviation over the entire concentration range, taking into account all technical repetitions (63 points of the calibration curve, 7 points for each of the 9 calibration levels) is CV (A) = 5.53% by peak area and CV (H) = 5.41% by peak height. The minimum measurement accuracy is achieved at concentration levels of 10 ng/ml and is 26% and 27.14% by area and height; at the level of 5 ng/ml and reaches 13% and 14. 14% by area and height; at the level of 50 pg/ml and reaches 10.41% by area (at the same time the accuracy of concentration measurement reaches 0.06% by peak height); and at the level of 25 pg/ml and reaches 15.81% by height (by peak area the measurement accuracy is 0.84%). However, exclusion of one or two technical measurement repeats from the 25 and 50 pg/ml levels results in a significant improvement in the measurement accuracy and does not exceed 3%. Nevertheless, at the lower levels of the calibration curve or at the detection limit, the limits of measurement accuracy fluctuations of up to ±20% of the expected calculated concentration are allowed. The intersection of the calibration curves with the horizontal distribution of the internal labeled HAEE standard (Figure 14) lies within the

concentration of 1 ng/ml, which fully meets expectations, since at this calibration level (the 1 ng/ml point) both components of the fixanal are in the state of an equilibrium mixture. This proves the correctness of the fixanal composition according to the dilution scheme used, the correctness of the initial composition of the concentration working solutions, and also opens up the possibility of using this dependence curve for measurement purposes.

The convergence of the peak area at the point of the equilibrium mixture is 0.7%, the convergence of the chromatographic peak height is 0.4%, which is close to the values of the calculated magnitude of the isotope interference effect of the signal.

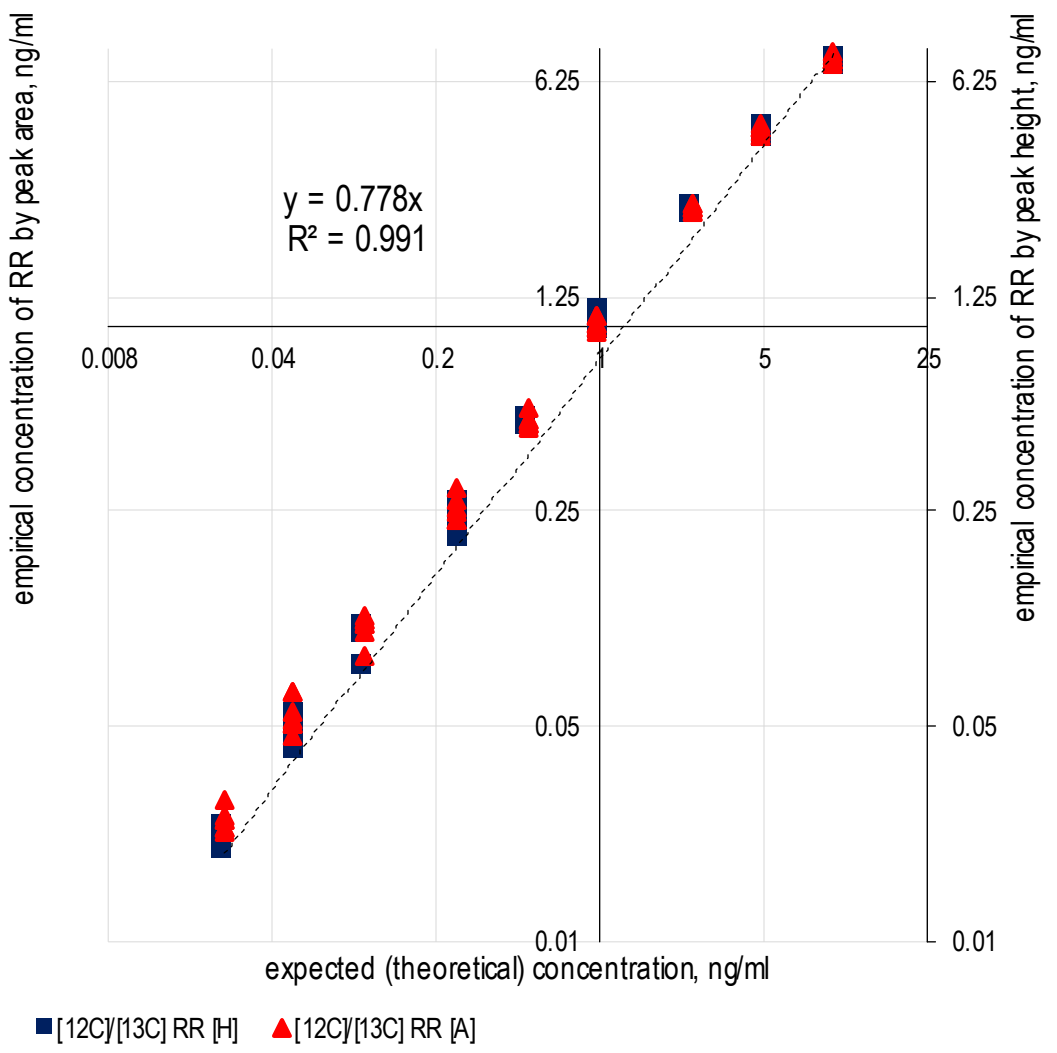

Figure 15. A linear plot of dependence of the empirical (measured) concentration of the analyte (non-labeled HAEE standard) on its expected (theoretical) concentration in the range from 0.025 ng/ml to 10 ng/ml by the relative response to the internal standard (labeled HAEE standard). The dependence is plotted by the area (triangular markers, red) and by the height (square markers, black) of the chromatographic peak. The determination coefficient was R^2 = 0.991 for the dependence over the entire dynamic range of concentrations.

Figure 15 demonstrates concentration at each calibration level versus the expected (theoretical) concentration. The curve of the analyte concentration versus the chromatographic peak area and the chromatographic peak height dependence passes through the equilibrium concentration point (calibration level number four; 1 ng/mL of non-labeled HAEE standard and 1 ng/mL of labeled HAEE standard) and intersects it. The determination coefficient was R^2 = 0.991. The distribution observed in the graph indicates a good positive correlation between the measured and expected values of the analyte concentration by the relative response to the internal standard over the entire concentration range. Such a dependence does not require the use of a weight correction factor and covers three orders of concentration (from 0.025 ng/mL to 10 ng/mL) with the same confidence interval. To determine the threshold of signal significance, a surrogate matrix solution in 0.5% formic acid and samples negative for standard were used. The threshold of significance was determined by a series of sequential measurements of each sample in ten technical replicates in the retention time interval specific for both labeled and non-labeled HAEE standards if it meets the identification criteria. A positive signal was considered conditionally positive and the probability value of the signal distribution was calculated throughout the analytical time of the elution gradient. As a result of the series of measurements, no interfering components that would contribute to the value of the true signal of non-labeled standard (in the surrogate matrix) or labeled HAEE standard were detected. Based on the results of the analysis of a series of negative samples, the presence of a signal at the level of an imaginary concentration of 0.004 ng/ml (4 pg/ml) with a standard deviation of σ=±87.5% was revealed. However, this concentration is outside the calibration curve and also outside the last calibration level (10 pg/mL) that was not included in the calibration curve. This calibration level can only be considered as an apparent detection limit under the given analytical conditions and can only be considered as a calculated value and, therefore, is not considered as being able to make a significant contribution to the true signal of the analyzed standard. It should be noted that the apparent signal of the negative sample does not meet the identification criteria and the quantification criteria, and the signal is at the level of the background signal and does not exceed it.

No signal corresponding to and fully meeting the identification criteria and quantitative evaluation criteria was found in the negative control sample. Integration of the area under the peaks is not possible due to the absence of a quantifier and specifier signal in the retention time intervals on the column characteristic of the standard. The signal recording spectra also do not meet the identification criteria, since, with the exception of the nominal mass-to-charge values, other criteria for differentiation and discrimination of the quantifier and specifiers are absent; the relative intensities of the specifiers and quantifier are beyond the limits specified in the signal identification criteria. The signal-to-noise level calculated under the chromatographic peak as the root mean square does not exceed 0.87–1.22 conventional units.

Thus, no significant signal capable of interfering with the signal of the tested standard and meeting the identification criteria was detected in the negative control sample. However, based on the analysis of data from all 10 measurements of negative control samples, a decision was made to set the cutoff threshold for the calculated concentration in the samples at 0.0041 ±0.0036 pg/ml (4.1 ±3.6 pg/ml, *n* =10) as the critical decision level. The cutoff threshold was set based on the detected signal that could meet the identification criteria for the quantifier ion. This concentration level was subsequently designated as the false positive signal limit (α-error). In the overwhelming majority of cases, when analyzing negative control samples, the peaks of specifier ions were not detected, or the signal of these ions was present, but integration of the peak area was not possible, which was regarded as non-compliance with the signal identification criteria

Section 4. Determination of Limits

Identification and reliability criteria: signal-to-noise level for the standard is not less than SNR>10, (according to the root-mean-square deviation algorithm). The maximum permissible deviation of the relative retention time on the column should be within RRT= ±1.0 %. The range of permissible deviation of the quantifier ion area is 90–110%. The range of deviation of the specifier ion area is not more than ± 10%, if the relative intensity is not less than 50% of the base ion; not more than ± 20% in absolute value, if the relative intensity is within 25% to 50% of the base ion; and not more than 15%, if the relative intensity is within 1% to 25% of the base ion). The accuracy of concentration measurement should be at least 10%.

To determine the basic analytical values for the qualitative and quantitative detection of the standard, only those data and calibration levels that strictly meet the signal identification criteria and the quantification criteria were used. The retention time on the column (RT) at all calibration levels shows high stability and is 3.26±0.013 minutes, which also meets the signal identification criteria, which allow a maximum deviation of the retention time of the analytical standard to the retention time of the internal standard of no more than Δ RRT = ±1.0 %. In this case, the deviation of the relative retention time on the column is Δ RRT = ±0.41% (for all technical replicates for all calibration points. The width of the chromatographic peak at half-height ($\omega_{0.5}$) shows high stability and is $\omega_{0.5}$ = 0.19–0.23 minutes (11.4–13.8 seconds). The stability of the peak width at half-height throughout the entire concentration range indicates that the stationary phase (chromatographic column) does not exceed the nominal binding capacity, and the ability to effectively chromatographically resolve the substance is maintained throughout the entire dynamic range of the calibration curve.

The asymmetry coefficient of the chromatographic peak is within the range γ = 1.089–1.104, indicating a slight excess of the right front of the peak at a height of *h* = 0.1 from the apex at high concentrations. This asymmetry distribution is normal and is due to the peculiarities of interaction with the carrier of the stationary chromatographic phase. The permissible values of the asymmetry coefficient deviation are Δγ = (- 0.7 / +1.6) and also largely depend on the concentration and load of the substance being detected. In this case, the asymmetry coefficient fully meets the requirements of the permissible limits of maximum and minimum deviation. The stability of the coefficient throughout the entire dynamic concentration range also indicates that the nominal binding capacity of the chromatographic column is not exceeded and that it maintains its effective resolving power, as well as proper dissolution of the standards in the mobile phase.

Taking into account the presented data on the calibration curve and the results of determining the signal significance threshold, the main analytical characteristics of the method for quantitative detection of the analyte (non-labeled HAEE standard) using the internal standard (labeled HAEE standard) were determined:

(1) Linearity limits (L): 0.025 ng/ml – 10 ng/ml

(2) Lower limit of quantification (LOQ): 0.025 ng/ml (25 pg/ml) – actual; 0.010962 ng/ml (10.962 pg/ml) – calculated.

(3) Critical concentration (*LC* = LOB): = 0.010022 ng/ml (10.022 pg/ml)

(4) Limit of detection (LOD): 0.010962 ng/mL (10.962 pg/mL)

(5) Lower limit of detection (LLOD): 0.010355 ng/mL (10.355 pg/mL)

(6) Method limit (ML): 0.00548 ng/mL (5.48 pg/mL)

The metrological characteristics given above were calculated as follows:

The critical concentration (LC, or LOB) was calculated using the formula:

$L_c = LOB = 1.645\sigma + \overline{R}$ (Formula 2)

where LOB is the negative sample signal limit, σ is the standard deviation of the negative sample signal, R is the average value of the negative sample signal. Thus, R = 0.0041, σ = 0.0036;

The lower limit of detection (LLOD) was calculated using the formula:

$LLOD = 1.645\sigma_c + LOB$ (Formula 3)

where LOB is the critical concentration, σ is the standard deviation at the minimum concentration point of the calibration curve. Thus, LOB = 0.010022, σ = 0.0002023.

The limit of detection (LOD) was calculated using the formula:

$LOD = LLOD + 3\sigma_c$ (Formula 4)

where LLOD is the lower limit of detection, σ is the standard deviation at the minimum concentration point of the calibration curve. Thus, LLOD = 0.010355, σ = 0.0002023.

The lower limit of quantification (LOQ) was calculated using the formula:

$LOQ = LOD + 10\sigma_c(|b|/k)$ (Formula 5)

where LOD is the detection limit, σ is the standard deviation at the minimum concentration point of the calibration curve, |b| is the modulus of the coefficient b in the calibration curve equation, k is the slope of the calibration curve. Thus, LOD = 0.010962, σ = 0.0002023, |b| = 0.012, k = 5541 (by chromatographic peak area).

The method limit (ML) was calculated using the following formula:

$ML = 2 \times LOQ$ (Formula 6)

where LOQ is the limit of quantitative detection. Thus, LOQ = 0.010962.

All the above analytical values are calculated in accordance with *Protocols for Determination of Limits of detection and Limit of Quantification, Approved Guideline. CLSI document EP17. Wayne, PA USA: CLSI; 2004* [1].

Taking into account the peculiarities of the physicochemical properties of the HAEE, namely the presence of three polar amino acid residues and blocking (neutralization) of the C- and N -terminal groups, it is advisable to isolate the standard from a complex biological matrix (human plasma or serum) under conditions optimized for anion exchangers. Nevertheless, at the initial stages of the extraction method development, several variants were tested, which were based only on liquid-liquid extraction (LLE), that is, the separation of liquid phases by density and relative hydrophobicity and the ability of the target substance to pass into the phase closest to it in physicochemical properties. The following were tested as the main extraction phase: (1) a mixture of acetonitrile with water (9/1, v/v); (2) a mixture of methanol with water (9/1, v/v); (3) 150 mM acetic acid (glacial); (4) a solution of 5% ammonium hydroxide in acetonitrile; (5) a solution of 5% ammonium hydroxide in methanol; (6) an aqueous solution of 7% trifluoroacetic acid and other solvents and their combinations (**Figure 16**). In many cases, the separation was carried out in the organic phase of ethyl acetate, dichloromethane, a mixture of ethyl acetate and n-hexane, tert-butyl methyl ether, or without separation in the organic phase if this was not possible (e.g., complete miscibility of ethyl acetate or tert-butyl methyl ether with methanol or acetonitrile).

Without going into details of the results obtained for each of the tested extraction methods at the first stages, we note that aprotic solvents turned out to be the least effective, while the addition of bases to the medium does not have a significant effect on the yield of the standard. For example, the use of acetonitrile showed the degree of extraction within 8%–12%, and when using ammonium hydroxide in acetonitrile, the degree of extraction was from 10% to 12%. Similar results were obtained when using weak acids in an aqueous medium: extraction in 150 mM acetic acid showed the degree of extraction at the level of 8% of the initial amount of HAEE standard. Perhaps the reason is insufficient deproteinization and denaturation of the protein components of the matrix, since acetic acid and standard A1 in a bound state precipitate along with proteins. However, increasing the concentration of acetic acid to 800 mM did not change the results of the extraction efficiency. Further testing of a strong acid (7% trifluoroacetic acid solution) showed a fairly satisfactory result in terms of the degree of extraction, which was up to 37% of the initial amount of the substance. At the same time, the level of the degree of extraction remained the same for the concentration of 5 ng/ml and 10 ng/ml and was 37.1±2.4% and 35.7±3.1%, respectively. However, there are several disadvantages to this method of isolating non-labeled HAEE standard as a cation exchanger. Firstly, the extraction takes place under extremely aggressive acidic conditions: a 7% aqueous solution of trifluoroacetic acid creates a reaction medium with a pH of ~ 0.5 (pH < 1), which significantly increases the risks of induced irreversible oxidation of both non-labeled HAEE standard itself (at the amino acid residue of histidine with the formation of 2-oxohistidine) and other components of the matrix, which in a modified (oxidized) form can acquire other activities and enter into chemical reactions or complex formation reactions with non-labeled HAEE standard. Thus, any of these options threatens a quantitative loss of the A1 standard and a decrease in the degree of extraction (**Figure 16**).

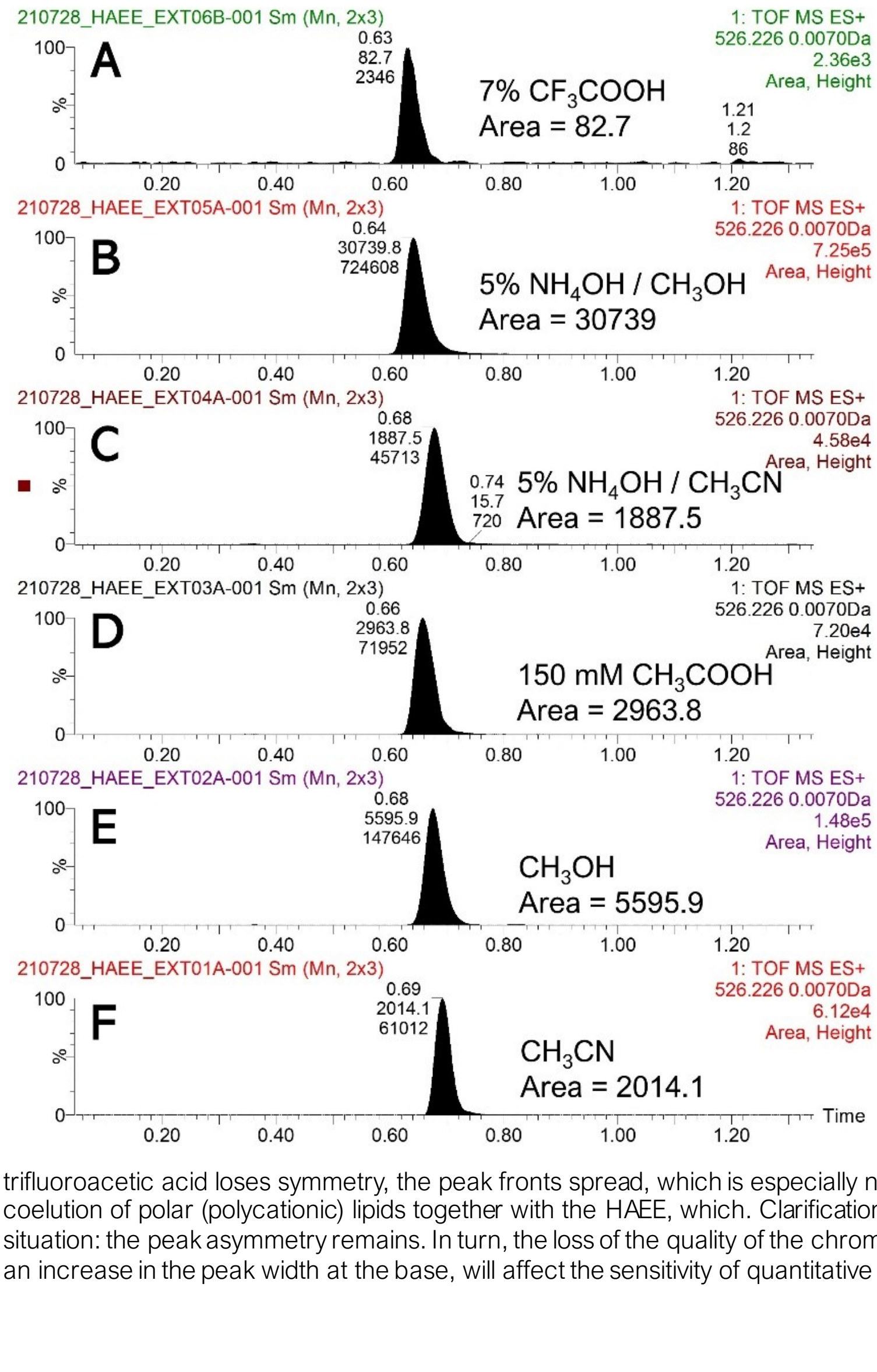

Figure 16. Extracted ion current chromatogram of non-labeled HAEE standard with m / z = 526.2256 (z = 1+) under different extraction conditions. The chromatographic separation conditions are not optimized. The concentration of standard in all the presented extraction cases is 3 ng/mL. (A) Extraction of standard in an aqueous solution of 7% trifluoroacetic acid, peak area A = 82.7; (B) extraction in methanol with 5% ammonium hydroxide, peak area A = 30739; (C) extraction in acetonitrile with 5% ammonium hydroxide, peak area A = 1887.5; (D) extraction in 150 mM acetic acid, peak area A = 2963.8; (D) methanol extraction, peak area A = 5595.9; (E) acetonitrile extraction, peak area A = 2014.1

During the MS analysis, oxidized forms of HAEE were not detected, but the recovery rate of 37% is far from the initial amount, which justifies concerns about the formation of complexes with other matrix components induced by an aggressive acidic environment. Secondly, trifluoroacetic acid, as a strong acid capable of independently forming salt complexes with peptides to forms internal salts. The solubility of such salts in an aqueous medium is changing, and they do not completely dissociate in the ionization source at high temperatures, which can also complicate the analysis due to the formation of multiple salt complexes (monomers and oligomers). In turn, this will be expressed either in a multitude of chromatographic peaks (corresponding to different salt forms) that differ in mobility properties, but belong to the same original substance, which complicates quantitative analysis and enforces the loss of HAEE substance at the isolation stage due to the different solubility of different salt forms. Thirdly, the chromatographic peak after isolation under conditions with trifluoroacetic acid loses symmetry, the peak fronts spread, which is especially noticeable in the leading peak. This may be due to the coelution of polar (polycationic) lipids together with the HAEE, which. Clarification of the extract in dichloromethane did not improve the situation: the peak asymmetry remains. In turn, the loss of the quality of the chromatographic peak shape, which is also accompanied by an increase in the peak width at the base, will affect the sensitivity of quantitative measurements, since the peak intensity and signal-to-

noise ratio decrease. Thus, we excluded aprotic solvents and weak acids as the least promising bases for the isolation of HAEE in the liquid phase.

More promising were the methods of extraction in methanol, as well as the additional use of bases in solvents. The total yield of HAEE in methanol was comparable to that noted after extraction under acidic conditions. However, in contrast to the resulting product of extraction with trifluoroacetic acid, the chromatographic peak after extraction with methanol was symmetrical, the right (tailing) front of the peak did not spread and was characterized by minimal horizontal diffusion and mass transfer. Addition of 5% ammonium hydroxide to methanol increased the total yield of the extraction product to 45%. However, the use of this approach also had some disadvantages. In particular, the high content of residual protein fraction in the solution. Methanol is a strong denatured alcohol and is used for deproteinization of biological samples. The mechanism of protein denaturation and precipitation occurs through the loss of the hydration shell, the rupture of hydrogen bonds with the solution outside the protein globule and, thus, the conformational eversion of the globule core to the solvent. The addition of a base, in this case a 5% ammonium hydroxide solution, promotes restructuring and the formation of an alternative network of hydrogen bonds of the globule with the external solvent, which, therefore, to some extent preserves the solubility of the protein globule in solution even in a denatured state. Thus, the addition of ammonium hydroxide on the one hand increases the yield of HAEE after extraction due to the neutralization of histidine and polarization of glutamic acid residues (isolation as an anion exchanger), on the other hand, it leaves residual solubility of denatured proteins, which can still be in a complex with HAEE, and also complicates both the process of drying the sample after extraction and the solubility of the dry residue of the extract in aqueous solutions, since it contains denatured proteins.

During the test trials, we used additional approaches to increase the degree of extraction and reduce the background from components of the biological matrix accompanying the extraction. For example, we used a number of organic solvents for additional purification and reduction of background extraction products. Maximum efficiency was achieved using ethyl acetate or its mixture with *n*-hexane (volume ratio 8:2). The use of this organic solvent, both alone and in combination, significantly reduces the background signal and does not distort the shape of the chromatographic peak of HAEE. But the most significant drawback was the partial solubility of HAEE in ethyl acetate: according to fractional analysis, up to 11% of the original amount of the substance passes into ethyl acetate. At high concentrations, this value can be neglected, but at low concentrations, a value of 11% loss can be critical. Collecting both fractions can correct the situation, but in this case, using ethyl acetate for additional purification is pointless, since the total fraction will contain all the original components before purification. Presumably, the partial solubility of HAEE in ethyl acetate is due to the weakly polar N-terminal acetyl group.

As a result, it was decided to modify the extraction method taking into account the available data and relying on polarity (anion exchanger under basic conditions) as the leading property of HAEE. For the efficient extraction of HAEE from human plasma or serum, a combination of delipidization in composite organic solvents and subsequent solid-phase purification (SPE) on mixed anion-exchange carriers (MAX) was used. Consequently, it was decided to isolate HAEE as an anion (negatively charged ion) in an alkaline medium; under these conditions, the amino acid residue of histidine remains electrically neutral. The use of a composite mixture of organic solvents during deproteinization allows, on the one hand, to denature proteins and precipitate them due to a complete loss of solubility. On the other hand, to preserve the solubility of HAEE, thereby reducing the likelihood of the formation of non-specific complexes with proteins in the precipitate or its capture by the surface of proteins during denaturation. The use of anion-exchange carriers for SPE purification allows the elimination of hydrophobic molecules that do not bind to the carrier, as well as neutral molecules and cations that can be eluted in a neutral medium or under acidic conditions.

The following extraction conditions on anion-exchange carriers proved to be optimal:

1. To one volume of blood plasma or serum (100 µl) add five volumes (500 µl) of a mixture of organic solvents methanol (CH_3OH), acetonitrile (CH_3CN) and water (H_2O) in volume ratios of 7:2:1 (v/v/v).

2. Incubate at a temperature of 40° C for 10 minutes with constant intensive stirring (at least 800 rpm).

3. Centrifuge at 10° C with an acceleration of 15,500 *g* for 10 minutes.

4. The supernatant liquid is collected and transferred to 5 ml of a 2.5% (v/v) ammonium hydroxide (NH_4OH) solution.

5. Five volumes of the organic solvent mixture are added to the precipitate again and steps (2) and (3) are repeated.

6. The supernatant liquid after secondary washing in a mixture of organic solvents is combined with the first fraction in 5 ml of a 2.5% ammonium hydroxide solution.

7. Conditioning Mixed Anion Exchange Carrier (Oasis ™ MAX 3 cc 60 mg; Waters, Inc.):

    7.1. Conditioning, washing, cleaning, elution and recovery are carried out using a vacuum station at a negative pressure in the range of -5…-10 mm Hg depending on the viscosity of the solvent and its interaction with the carrier: the worse the interaction with the carrier, the higher the negative pressure, and vice versa. The higher the viscosity of the solvent, the lower the negative pressure in the vacuum station chamber.

    7.2. Conditioning with 2 ml acetonitrile.

    7.3. Conditioning with 2 ml methanol.

    7.4. Conditioning with 2 ml of 4% ammonium hydroxide aqueous solution.

    7.5. Applying the sample to 5 ml of an aqueous solution of 2.5% ammonium hydroxide (see point (6)).

    7.6. Washing the carrier from salts in 2 ml of 2.5% aqueous ammonium hydroxide solution.

7.7. Elution of neutral molecules in 2 ml of water.

7.8. Elution of hydrophobic molecules in 2 ml of an aqueous solution of 60% methanol.

7.9. Elution of the target component (standard A1) as an anion in 1 ml of a solution of 3% formic acid ($HCOOH$) in acetonitrile.

8. The collected fraction with standard A1 is dried under vacuum at a temperature of 30 ˚C for up to 45 minutes.

9. The dry residue obtained after drying is reconstituted in ¼ of the original sample volume (i.e. 25 µl) in an aqueous solution of 0.5% formic acid.

Recommendations and Commentaries to the proposed Extraction Chain of Custody

(1) The use of methanol in combination with acetonitrile is preferable to the use of any of these solvents separately. The composite use allows for effective denaturation of the protein fraction due to the properties of acetonitrile and methanol and, at the same time, to preserve the effective solubility of HAEE due to methanol and water. It should be noted that the ratio of acetonitrile to methanol in volume fractions of 2:7 is also optimal. Shifting the ratio towards a higher acetonitrile content leads to the formation of a viscous precipitate of the protein fraction and a more rapid formation of the precipitate. Moreover, the precipitate formed by acetonitrile does not pass into a suspended state during secondary extraction (washing, point (5)) in contrast to the precipitate formed by methanol, which freely passes into a suspension during recovery.

(2) It should be remembered that the calculated volume of the supernatant obtained in point (1) is 600 µl (100 µl of biological material and 500 µl of the composite mixture of organic solvents), but in reality, the volume will be less than the calculated one, since the protein sediment has a tendency to accumulate a small fraction of water during denaturation, thus part of the volume of solvents will be compensated by the protein sediment. The supernatant should be quantitatively collected during the primary precipitation of proteins and secondary washing of the sediment, i.e. taking into account the compensation volume, which is about 10% of the calculated volume. Thus, during the primary sedimentation and secondary washing of the sediment, no more than 550 µl of the supernatant should be collected.

(3) The incubation temperature has a slight effect on the extraction efficiency. When testing in the range from 20 ˚C to 70 ˚C, it was shown that up to 40 ˚C the extraction efficiency increases (by approximately 4–5% for every 10 ˚C of incubation temperature), but then the degree of denaturation reaches a plateau (up to 50 ˚C) and begins to decrease (at a temperature of 60 ˚C) by 12–15%. The choice of the optimum at 40 ˚C is due to the fact that this temperature is sufficient for mild denaturation of proteins, as an auxiliary factor to chemical denaturation with a mixture of organic solvents. However, at this temperature, the probability of chemical reactions between the components of the biological matrix, which can affect the chemical identity of HAEE and, thus, the degree of its extraction, is low. Temperatures above 60 ˚C will obviously have a positive effect on the denaturation of the protein fraction but will also activate and catalyze reactions in the biological matrix itself, which may lead to a change in the structure of HAEE.

(4) Dilution by transferring the supernatant to an aqueous solution of 2.5% ammonium hydroxide necessarily reduces the concentration of organic solvents and inorganic salts in the biological matrix, which is favorable for loading (applying) the standard to the ion-exchange carrier. Despite the ion-exchange nature of the carrier, test experiments have shown that when applying a sample in organic solvents (methanol, acetonitrile), HAEE is detected in significant quantities in the "breakthrough" fraction, i.e., low affinity of the ligand to the carrier is noted.

(5) Preliminary experiments showed that the use of ammonium hydroxide at a concentration of 2.5% is as effective as a concentration of 4%. Sufficient buffer capacity of the solution (pH >11) provides the necessary conditions for loading onto the anion-exchange carrier and polarization of the carboxyl groups of glutamic acid (pKa = 2.19).

(6) The use of low temperatures (10 ˚C) during centrifugation is an additional auxiliary factor for protein precipitation. Thus, the solubility of the protein fraction is additionally reduced, whereas the temperature factor will not play a major role in the solubility of HAEE in the range of concentrations studied (up to 0.025 ng/ml) due to the polarity and sufficient solubility of the organic solvent mixture in the aqueous fraction. Moreover, the density of water changes more in the temperature range from 30 ˚C to 10 ˚C (995.41–999.77 kg/m^3) than in the range from 10 ˚C to 2 ˚C (999.77–999.94 kg/m^3), which also ensures stable solubility of HAEE in the aqueous fraction at a given temperature regime.

(7) When conditioning the anion exchange carrier, it is recommended to use a 4% ammonium hydroxide solution to fill the resin container.

(8) It is not recommended to exceed negative pressure values less than -10 mm Hg to avoid mechanical damage to the resin and loss of the binding properties of the carrier. The binding is electrostatic and proceeds better with a slower diffusion of the ligand through the affinity chemical groups of the carrier.

(9) It is not recommended to add acids to the solution for elution of neutral molecules (60% methanol solution in water), as this may reduce the efficiency of binding of HAEE to the carrier and reduce its quantitative yield.

(10) It is recommended to use a solution of 3% formic acid in acetonitrile rather than in water for elution of HAEE, since this significantly reduces the correction time under vacuum (the boiling point of acetonitrile is 82˚C, the density is 0.786 kg/m^3 the dynamic viscosity is 0.38 mPa × s (for water the dynamic viscosity is 1.0016 mPa × s)).

(11) The resulting dry residue after drying under vacuum is usually well soluble in aqueous solutions without residue. However, it is recommended to clarify the solution by ultracentrifugation (at 10,000–12,000 *g*) at low temperatures (10° C) for 5 minutes to avoid damaging microparticles getting into the stationary phase of the chromatographic column or into the injector microcapillary system.

(12) It is not recommended to use a concentration of formic acid in the recovery solution (item 9) higher than 0.5%, since its excess content can lead to ion suppression and to a slowdown in the evaporation of the spray in the ionization source chamber, which will need to be compensated for by increasing the temperature in the chamber and can lead to the formation of salt deposits (formates and their aggregates) at the entrance to the capillary and reduce the transmission of ions.

(13) Drying under vacuum at high temperatures (45° C and above) should be avoided, as this may catalyze the esterification and formylation reactions with formic acid. The risk of formylation was one of the leading reasons, along with the length of the drying step, for which elution of the standard from the support was carried out in acetonitrile, an aprotic medium.

(14) Despite the apparent obviousness, it is necessary to remind about the use of plastic containers during sample preparation, and not glass, since due to the peculiarities of the physicochemical properties, the peptide sticks to the glass surface, which entails quantitative losses of the substance. The only exception may be deactivated glass, for example, when transferring the A1 standard solution for HPLC-MS analysis into specialized inserts made of deactivated glass with a nominal volume of 150 or 200 µl.

Section 6. Recovery of HAEE After Extraction and Matrix Influence Factor

The recovery was calculated relative to the analytical standard of the corresponding concentration in the biological matrix (empirical degree of extraction) and relative to a pure solution of the analytical standard of the corresponding concentration (theoretical deg ree of extraction). The extraction of the standard was carried out using solid-phase extraction on a mixed anion-exchange carrier (SPE-MAX), as described above in points in Section no. 5. A 3% solution of formic acid in acetonitrile was used as the extracting (eluting) solvent. The procedure for extracting the substance from the biological sample was carried out identically for different concentrations of the standard (at a concentration of 1 ng/ml and 5 ng/ml). The theoretical recovery was calculated relative to the standard sample in a pure 0.5% formic acid solution, while the empirical recovery was calculated relative to the post-extraction biological matrix to which standard was added to the appropriate final concentration.

$$R_t = \frac{C(StdA2_t) - C(StdA2_{ex})}{C(StdA2_t)}, \quad R_e = \frac{C(StdA2_{mtrx}) - C(StdA2_{sp})}{C(StdA2_{mtrx})} \quad \text{(Formula 7)}$$

where C ($StdA2_t$) is the concentration of labeled HAEE standard in a pure solution of 0.5% formic acid; C($StdA2_{ex}$) is the concentration of labeled HAEE standard after extraction from a pure solution; C($StdA2_{mtrx}$) is the concentration of non-labeled HAEE standard added to the biological matrix after extraction; C ($StdA2_{sp}$) – concentration of labeled standard isolated from the biological matrix. To calculate the theoretical recovery, 1 ng/ml and 5 ng/ml of labeled HAEE standard were taken as the standard concentration; to calculate the empirical recovery, the concentration was taken to be the value that was measured for non-labeled HAEE standard using the previously constructed calibration curve (Figure 17 and Table 4).

Table 4. Main parameters of recovery (extraction degree) of labeled HAEE standard at concentrations of 1 ng/ml and 5 ng/ml from pure solution and from biological matrix. The recovery was calculated taking into account the dilution factor d = 4 in accordance with paragraph (9) of the Extraction Method (Section no. 5). Measurements were carried out in five technical replicates for each concentration point and for each matrix sample. The table shows the calculation of the recovery based on the area of the chromatographic peak of labeled HAEE standard (m/z = 528.2329 (z = 1+)). The matrix effect was calculated between a sample of pure solution of non-labeled HAEE standard and a sample of the biological matrix after extraction, to which the standard was added to the corresponding final concentration.

Standard concentration, ng/ml	Degree of extraction from pure solution	Degree of extraction from biological matrix	Matrix effect
1	0.9632±0.028	0.7014±0.024	0.9337±0.013
5	0.9813±0.011	0.7073±0.022	0.9836±0.008

The obtained data indicate a high degree of extraction of labeled HAEE standard from a pure solution, while a fivefold difference in concentration (1 ng/ml and 5 ng/ml) has no dramatic effect on the recovery from a pure solution (Table 4). During extraction from a biological sample (biological matrix), losses of about 30% are observed for both concentrations (Table 4), which is significantly lower than the expected losses. It is significant that the convergence of the results when isolating the concentration of 1 ng/ml and 5 ng/ml in terms of the degree of extraction both from a pure solution and from a biological matrix indicates the absence of a significant influence of the sample dilution effect, which is also indirectly confirmed by the previously established interlevel response coefficient (see Table 3), which fully corresponds to the concentration ratio between adjacent concentration levels.

To determine the matrix effect of ion suppression and potentially interfering components, a prepared sample of the biological matrix after extraction was used, which was reconstituted in 0.5% formic acid containing 1 ng/ml or 5 ng/ml of labeled HAEE standard (Figure 17). Measurements were performed against a sample of labeled HAEE standard in a pure solution with the appropriate concentration (Table 4). The matrix effect for labeled HAEE standard at a concentration of 5 ng/ml was 98.36%, i.e. signal suppression by f = 6.62%, for the standard at a concentration of 1 ng/ml the matrix effect was higher and was 93.37%, i.e. signal suppression by f = 6.63%. Due to the fact that the matrix effect exhibits unambiguous ion suppression at different concentrations of HAEE standard, it was suggested that the suppression is largely due to impurity components of the solvent.

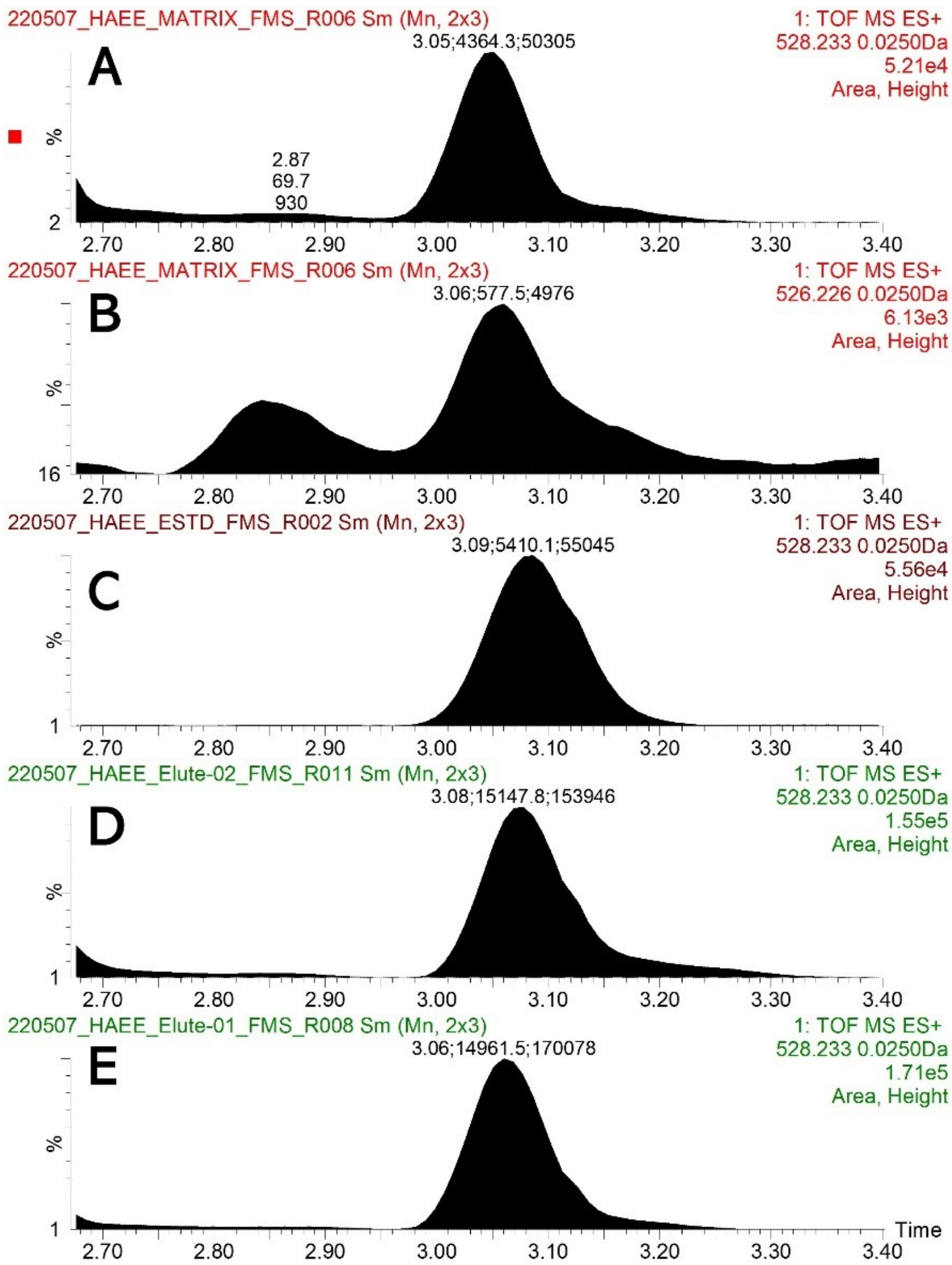

Figure 17. Chromatogram of the isolated ion current of labeled HAEE standard with m/z = 528.2329 (A) added to a final concentration of 1 ng/ml into a biological matrix after extraction to assess the matrix effect; (B) isolated ion current chromatogram of the endogenous (non-labeled) HAEE substance corresponding with m/z = 526.2256 (z = 1+); (C) isolated ion current chromatogram of labeled HAEE standard with m/z = 528.2329 in a pure solution (external standard) with a concentration of 1 ng/ml; (D) after elution in acetonitrile with 3% formic acid (labeled HAEE was added to the biological matrix before extraction). (E) after elution in an aqueous solution of 3% formic acid (labeled HAEE standard was added to the biological matrix before extraction).

However, taking into account the calculated value of the degree of extraction (within 70%), this value of ion suppression in the specified concentration range can be neglected in further calculations, whereas at lower concentrations the matrix effect can be compensated by the effect of isotopic distribution between labeled and non-labeled HAEE molecules. Note that the matrix effect and the recovery degree of extraction from the biological sample were determined only using labeled HAEE standard, which is chemically identical to non-labeled HAEE standard. It is difficult to carry out the same calculations and experiments for non-labeled HAEE standard due to its endogenous nature (Figure 17), which requires taking into account the additive signal with a large uncertainty for endogenous HAEE.

Section 7. Study of Stability of the HAEE Substance

Despite the apparent simplicity of the study, determining the stability of any substance is in fact a non-trivial task. In essence, the study of stability is determined by many physical, chemical and biochemical factors, the combination of which gives the researcher the key to understanding the rate of degradation of the substance, the necessary measures for transporting and storing the substance, its half-life, on which depends the reproducibility and truth of both the qualitative and quantitative results of instrumental measurement. The study of the stability of biological substances, especially those of endogenous origin, is complicated by the great uncertainty of the influence of the environment of the biological matrix itself, which can be predicted, in most cases, only indirectly, based on the initial physicochemical properties of the substance being studied. For example, modeling of peptide degradation by aminopeptidases and carboxypeptidases, utilization of proteins by ubiquitin machinery, transformation of steroids through a series of successive reactions of hydroxylation, bond restoration, reduction of radicals in the cytochrome system, etc. However, the stability of low-molecular compounds is also associated with such factors as non-specific affinity for some transport protein molecules capable of carrying away the target substance during sample preparation; the ability to form non-specific complexes with surrounding molecules induced by changes in the environment at the preanalytical stage; even the material of the walls of the container (test tube) in which the target substance is stored can have a significant effect on the apparent stability (binding of lipophilic molecules to walls made of polypropylene or polyethylsulfonate).

In this work, the study of the cyclic and continuous stability of labeled HAEE standard in a pure solution environment and in a biological matrix (EDTA-$2K^+$ human blood plasma) was carried out under the same hermetic conditions, regardless of the storage temperature conditions and the duration of observations (Figure 18).

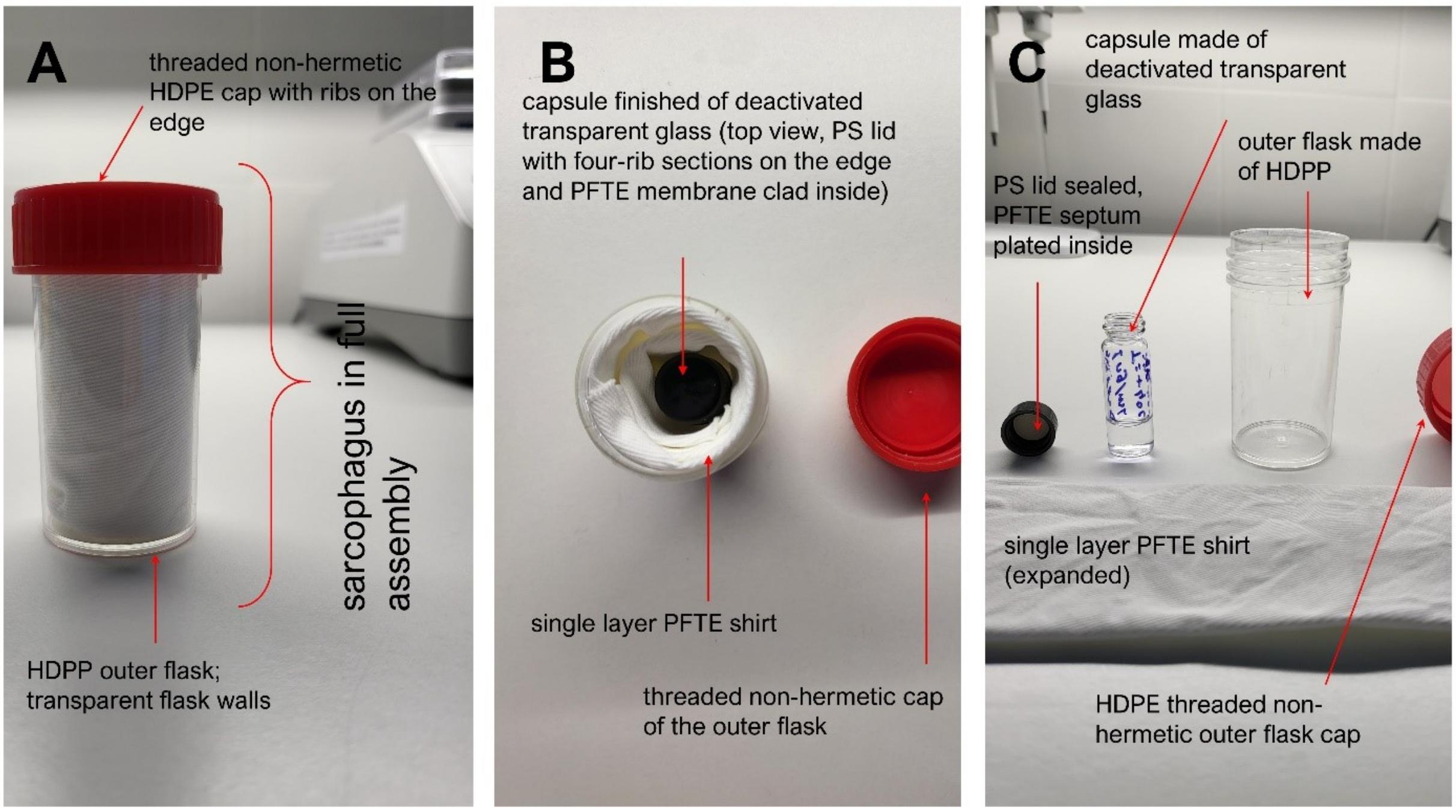

Figure 18. Schematic diagram of the sarcophagus for storing labeled HAEE standard in a pure solution environment and in a biological matrix environment. The sarcophagus for storing the fully assembled sample (A) throughout the entire observation period consists of a flask (A, B) made of transparent high-pressure polypropylene with an external threaded non-hermetic cap made of high-pressure polyethylene (B). The inner walls of the flask are lined with a single-layer polyfluoroethylene jacket (B, C). Inside the flask is a capsule (C) made of deactivated glass with a solution of labeled HAEE standard in 0.5% formic acid. The capsule is closed with a polystyrene threaded hermetic cap, plated with a polytetrafluoroethylene membrane from the inside (C).

All observation samples were stored in special sarcophagi (Figure 18), consisting of several elements. The outer part of the sarcophagus is represented by a flask made of transparent high-pressure polypropylene (HDPP) with a screw cap (non-hermetic) made of high-pressure polyethylene (HDPE). The inner walls of the flask are surrounded by a jacket made of single-layer polytetrafluoroethylene (PTFE). Inside the flask there is a capsule made of deactivated glass (*manufactured by Duran Wheaton Kimble, serial number 0000030206, catalog number 224722, model Vial 4 ML E - C Sample Clear Rubber Lined Cap*) with a hermetically sealed polystyrene (PS) ground-in cap lined on the inside with a polytetrafluoroethylene septum. The capsule is filled with either a solution of standard A2 in 0.5% formic acid or with the standard in a biological matrix at a final concentration of 1 ng/ml in both cases.

7.1. Continuous Storage: Stability of HAEE In the Environment of a Pure Solution

The stability of a substance in a pure solution environment is determined mainly by a number of physical factors: storage temperature and its fluctuations, humidity of the environment, reaction level of the environment (pH), natural convection of the solvent, physical properties of the solvent (density, viscosity, boiling and freezing point) in which the target substance is stored, etc.

In the case under study, the stability of labeled HAEE substance was studied in a pure 0.5% formic acid solution in water with an initial conductivity of less than 0.5 µS (µS; microSiemens) and a specific resistance of more than 18.2 MΩ (megaOhm). The reaction of the 0.5% aqueous formic acid solution is pH = 2.4±0.2 at a temperature of 20±2 °C, which generally preserves the carboxyl groups of HAEE standard in a neutral state, and secondary amines in a state with a partial positive charge. Thus, in terms of chemical properties, the solvent medium for HAEE standard is represented by at least 99.5% water and no more than 0.5% formic acid under the initial conditions of the study. The calculated density of such a solution [I] is ρ = 0.998115 g/cm^3, which differs from the density of the main component of the solution (water) by only 0.11% [2]. The equilibrium concentration of labeled HAEE standard in such a solvent is 1 ng/ml, which introduces an infinitesimal effect on the change in the density of the solution, and which for this reason can be neglected.

It is essential that storage of any substance in solution at room or low temperature is in any case associated with the processes of natural convection of the solution and its components, that is, with parallel processes of evaporation and condensation [3]. Despite the closed nature of the storage system in our study (all prepared standards are in a glass test tube made of deactivated glass with a nominal volume of 4 ml and closed with a plastic lid, while the test tubes at the initial level are filled to no more than 50% of the nominal volume, that is, the volume of the standard solution is 2 ml), the processes of natural and forced convection of the solvent occur even in such a system, which is facilitated by two main factors: firstly, the presence of a gas phase in a closed system (air phase above the surface of the solution in the test tube) and, secondly, the temperature of the external environment [4]. When the free surface of water comes into contact with air, heat and mass exchange occurs under the influence of external factors. During diffusion, steam from the saturated surface of the solvent is transferred to the gas phase. The rate of molecular evaporation is expressed in terms of the mass transfer coefficient k_G due to the pressure gradient and the driving force of the partial pressure (below) [5].

$$N_{mol} = k_G \times \left(p_{v,f} - p_{v,G}\right) \text{ (Formula 8)}$$

Using the equation of state of an ideal gas, we find the rate of molecular evaporation from the surface:

$$N_{mol} = k_G RT\left(C_{v,f} - C_{v,G}\right) = k_c\left(C_{v,f} - C_{v,G}\right) \text{ (Formula 9)}$$

Where k_c is the mass transfer coefficient due to the concentration gradient of substances [6,7]. Thus. The mass flow rate of a substance (solution) using the evaporation coefficient and absolute humidity can be represented as follows:

$$N_{H2O} = k_c M_{H2O}\left(C_{v,f} - C_{v,G}\right)\frac{\rho_f}{\rho_G} = \sigma\left(Y_f - Y_G\right);\ \sigma = k_c \rho_G;\ Y = \frac{c_v M_{H2O}}{\rho_G} \text{ (Formula 10)}$$

Where σ is the evaporation coefficient, Y is the absolute humidity, M_{H2O} – molecular mass of water C_v, – vapor concentration. Since in the case of a flat liquid surface the form resistance is negligible compared to the frictional resistance, the dependence of heat and mass transfer on the transfer of momentum can be used to determine the amount of evaporated water. In this paper, we can fairly consider the evaporation of water from the surface as a result of forced convection, since the system under consideration is conditionally closed and during evaporation of water, part of the condensate will be removed from the system as a result of its incomplete tightness, and the other part will form condensate. At the same time, with an increase in the storage temperature, one can expect an increase in the excess vapor pressure in the system during molecular evaporation, which in the gas phase will leave the closed system, which can create an imaginary increase in the concentration of the monitored component (HAEE) in the solution at a certain stage of observations, since the storage temperature of the solution is clearly insufficient for the molecular evaporation of HAEE from the surface. Thus, the calculated value of the mass flow rate of the substance (0.5% formic acid solution in water) at a solution temperature of about 22 °C and a gas phase temperature (reduced) of about 15°C and a humidity of 37% should be about 0.107 g/(m^2 × s) (calculated value). It should be noted that formic acid present in the solution at a concentration of 0.5% (vol./vol.) will provoke the formation of a hydrate shell around its molecules, which reduces the molar amount of water available for evaporation from the surface, but also contributes to an imaginary increase in the concentration of HAEE in the solution. The results obtained as a result of observations, therefore, will require correction for the rate of evaporation of the solution from the surface and the change in the volume of the solution as a result of forced convection.

The stability study was conducted for a solution of HAEE at a concentration of 1 ng/ml in three temperature modes: at a temperature of -20° C (symbolic designation FZ), at a temperature of 5±2° C (symbolic designation FR), and at a temperature of 21±2° C (symbolic designation RT). The temperature and humidity in the room were monitored only in the RT mode (Figure 19).

[I] The density of water at a temperature of 20°C is ρ = 0.997 g/cm^3; the density of formic acid at a temperature of 20°C is ρ = 1.223 g/ cm^3

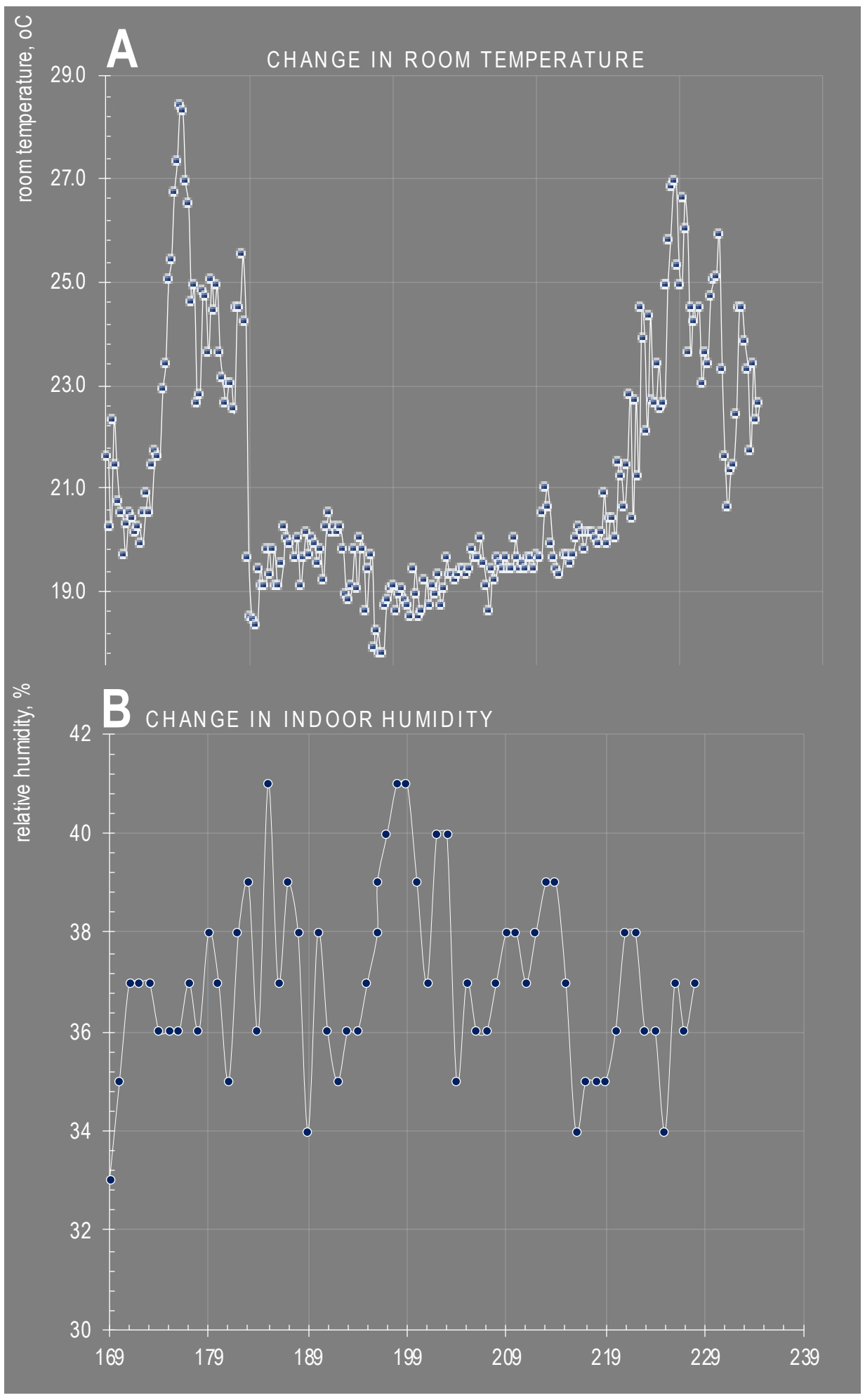

Figure 19. The inspected of temperature **(A)** and humidity **(B)** in the room during storage of a solution of HAEE at a concentration of 1 ng/ml in 2 ml of a solution of 0.5% formic acid in water in a test tube made of deactivated glass with a plastic cap.

The prepared standards for each storage mode were presented in the form of a 2 mL solution in 0.5% formic acid in water in glass test tubes (vessel walls made of deactivated glass) with plastic caps. At the same time, for the standards in the temperature mode of 5±2ºC (FR^S) and at a temperature of 21±2ºC (RT^s), solutions of 0.5% formic acid in a volume of 1 ml were prepared in identical test tubes (nominal volume 4 ml) for control weighing (test tubes with solution and cap) on Sartorius analytical balances *QUINTIX 2150-10 RU* (d = 0.01 mg, e = 1 mg).

Monitoring of 0.5% formic acid solution standards under continuous storage conditions in RT^s modes for 61 days and FR^s showed that over time at room temperature there is a pronounced tendency for the solvent to evaporate from the surface layer, while at a temperature within 5±2 ºC, on the contrary, a reverse convection process can be noted. Thus, during 61 days of observation in the sample at room temperature, the volume mass of the solution decreased by 0.13662 g (136.62 mg), and the mass of the solution at a temperature of 5±2 ºC increased by 0.00544 g (5.44 mg). Both processes have a well-defined monotonic tendency Figure 20, however, the evaporation process from the surface occurs at a higher rate than the reverse convection process despite the fact that the surface of the solution does not have direct access to atmospheric air, and the flask itself (vial with solution) is an imitation of an adiabatic system. The evaporation process is characterized by linear regression and occurs at a rate of about 8.255 nL/s×cm2 (Figure 20), thus, over the course of a year the evaporation volume will be 817.48 µL, that is, the remaining volume of the solution will be 182.51 µL, and the imaginary concentration of substance A2 at absolute stability will be 5.47 ng/ml.

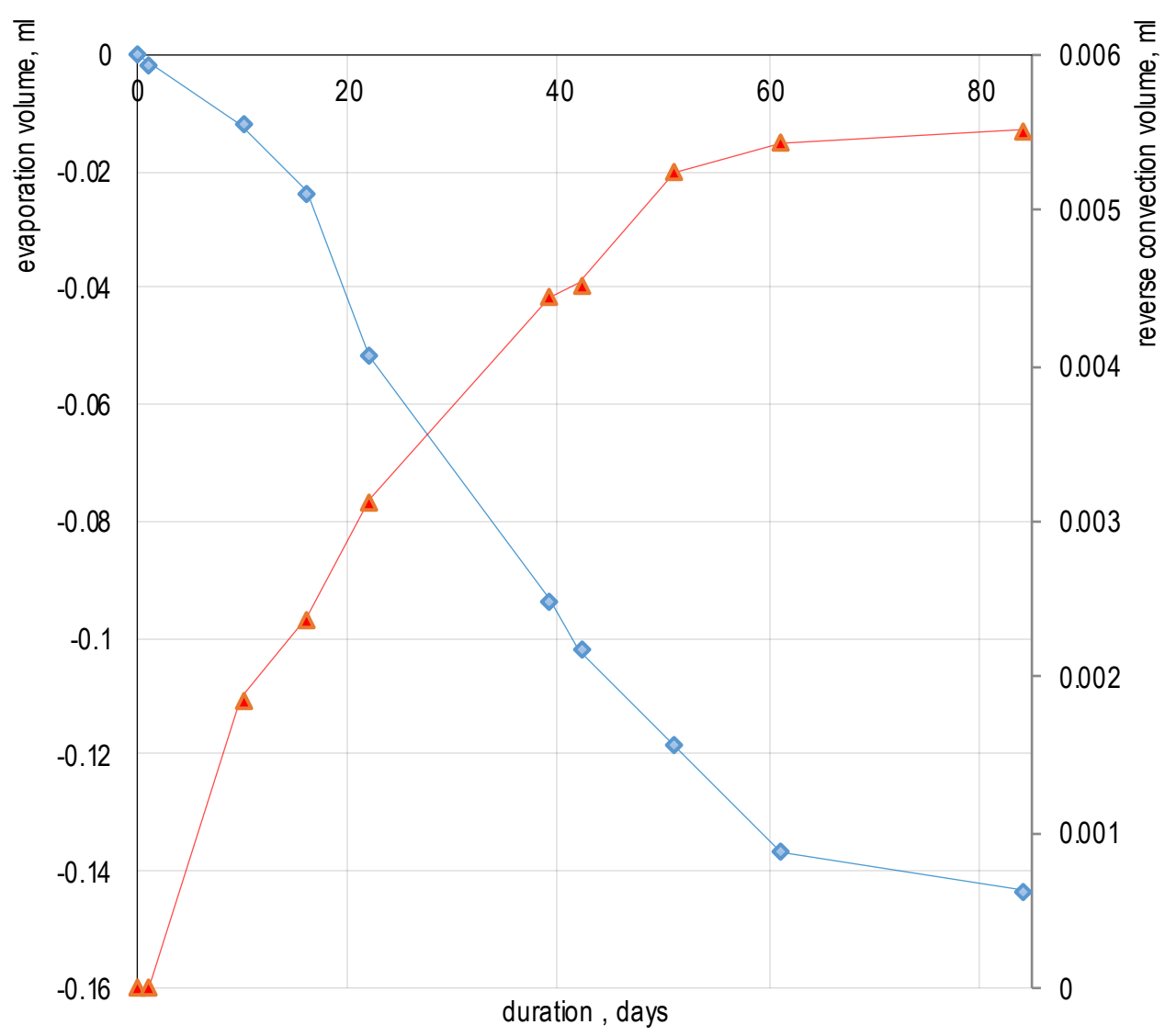

Figure 20. Changes in solvent volume in standard samples during 85 days of observation at a temperature of 21°C (RT mode; blue line on the graph) and at a temperature of 5°C (FR mode; red line on the graph). In the FR mode, a stable tendency of reverse convection is observed, whereas in the RT storage mode, stable evaporation of the solution volume is observed.

At the same time, the reverse convection process is also well expressed in the sample at a temperature of about 5ºC, but its speed is significantly lower compared to the evaporation process (Figure 20). Thus, calculations showed that the reverse convection speed is 0.329 nL/s×cm^2, i.e. the total accumulated volume for one calendar year will be 32.55 µL, and the imaginary concentration of HAEE, subject to absolute stability, will decrease by less than 4% (by 3.36%) over this period.

Table 5. Changes in the signal of **HAEE** in 0.5% formic acid solution by area (A) and by height (H) of the chromatographic peak in full MS and in high-resolution SRM mode (HR - SRM) during 60 days of observation at a temperature of -20°C (symbol FZ), and during 90 days of observation at a temperature of 5±2 °C (symbol FR) and at a temperature of 21±2°C (symbol RT). The repeatability of the measured concentration was determined by the ratio of the calculated concentration by height to the calculated concentration by the area of the chromatographic peak.

Chromatographic peak height	Duration of observation, days		0	1	6	19	24	50	61	90
	RT	R01	635093	597251	639450	702185	725147	701762	744304	752313
		R02	635209	620684	640487	704139	711153	723261	735778	752562
		R03	625527	618091	640339	709898	708942	733621	705696	752987
	Average value		631943	612008.7	640092	705407	715080.7	719548	728593	752621
	Relative standard deviation		0.88%	2.10%	0.09%	0.57%	1.23%	2.26%	2.78%	0.05%
	Calculated concentration, ng/ml		1	0.968	1.013	1.116	1.132	1.139	1.153	1.191
	FR	R01	648886	634385	685488	727616	742750	764252	810378	794350
		R02	648846	637838	677025	725485	736735	742803	794034	795016
		R03	649835	633919	654907	703599	726357	733229	768601	794826
	Average value		649189	635381	672473	718900	735281	746761	791004	794826
	Relative standard deviation		0.09%	0.34%	2.35%	1.85%	1.13%	2.13%	2.66%	0.05%
	Calculated concentration, ng/ml		1	0.979	1.036	1.107	1.133	1.15	1.218	1.224
	Duration of observation, days		0	1	5	10	20	30	60	
	FZ	R01	1206382	1213425	1131432	1126877	1128001	1112146	1115418	
		R02	1205397	1198463	1138491	1108695	1123806	1118090	1115614	
		R03	1198741	1182233	1129874	1144587	1122979	1127409	1119817	
	Average value		1203507	1198040	1133266	1126720	1124929	1119215	1116950	
	Relative standard deviation		0.35%	1.30%	0.41%	1.59%	0.24%	0.69%	0.22%	
	Calculated concentration, ng/ml		1	0.995	0.942	0.936	0.935	0.93	0.928	
Chromatographic peak area	Duration of observation, days		0	1	6	19	24	50	61	90
	RT	R01	42485	37305	42956	46654	46429	45218	54309	55809
		R02	42831	40060	43268	46021	46537.5	46858	53904	55675
		R03	43723	39952	43305	45241	44100.5	48061	53590	55819
	Average value		43013	39106	43176	45972	45689	46712	53934	55768
	Relative standard deviation		1.48%	3.99%	0.44%	1.54%	3.01%	3.06%	0.67%	0.14%
	Calculated concentration, ng/ml		1	0.909	1.004	1.069	1.062	1.086	1.254	1.296
	FR	R01	43824	40120	44301	47464	49550	50926.5	56456	56227
		R02	43710	39747	45825	48139	48381	49917.5	55839	56132
		R03	43917	39709	46206	46995	47721	48420.5	55337	56198
	Average value		43817	39859	45444	47533	48551	49755	55877	56186
	Relative standard deviation		0.24%	0.57%	2.22%	1.21%	1.91%	2.53%	1.00%	0.09%
	Calculated concentration, ng/ml		1	0.91	1.037	1.085	1.108	1.136	1. 275	1.282
	Duration of observation, days		0	1	5	10	20	30	60	
	FZ	R01	80996	80986	75533	74122	74317	74593	74888	
		R02	80365	80684	73870	74359	73879	74429	74913	
		R03	80971	79580	75966	74129	74776	74082	74729	
	Average value		80777	80417	75123	74203	74324	74368	74843	
	Relative standard deviation		0.44%	0.92%	1.47%	0.18%	0.60%	0.35%	0.13%	
	Calculated concentration, ng/ml		1	0.99 6	0.93	0.91 9	0.92	0.92 1	0.926	
A/H results		RT	100.00%	106.52%	100.91%	104.44%	106.53%	104.84%	91.95%	91.86%
		FR	100.00%	107.59%	99.88%	102.08%	102.22%	101.30%	107.30%	95.48%
		FZ	100.00%	99.99%	101.25%	101.91%	101.59%	101.01%	100.17%	

The signal of HAEE for concentration calculation was measured in three technical replicates in full frontal scanning and MRM modes at the initial observation point (on the day of standard preparation), 1 day after solution preparation, 6 days, 19 days, 24 days, 50 days, 61 days and 90 days after the start of observations. The observed concentration was calculated based on the chromatographic peak area (A) and the chromatographic peak height (H) with subsequent determination of the convergence of the results for these values. The relative standard deviation of the signal was calculated for three technical replicates. The dynamics of concentration change was determined by normalizing to the concentration of HAEE in solutions at the initial observation point (zero point), which was taken as a conditional 1 ng/ml concentration. The results of observing changes in the signal and concentration of HAEE over time under different storage temperatures are presented in Table 5 and Figure 21. The data for the FR/RF and FZ mode are presented separately, since the observations in the RT/FR and FZ modes were not synchronous with a lag of 30 days. In particular, by the time the 61-day observation point was obtained in the RT/FR modes, only the 30-day observation point was reached for the FZ mode. However, the observation data were later synchronized.

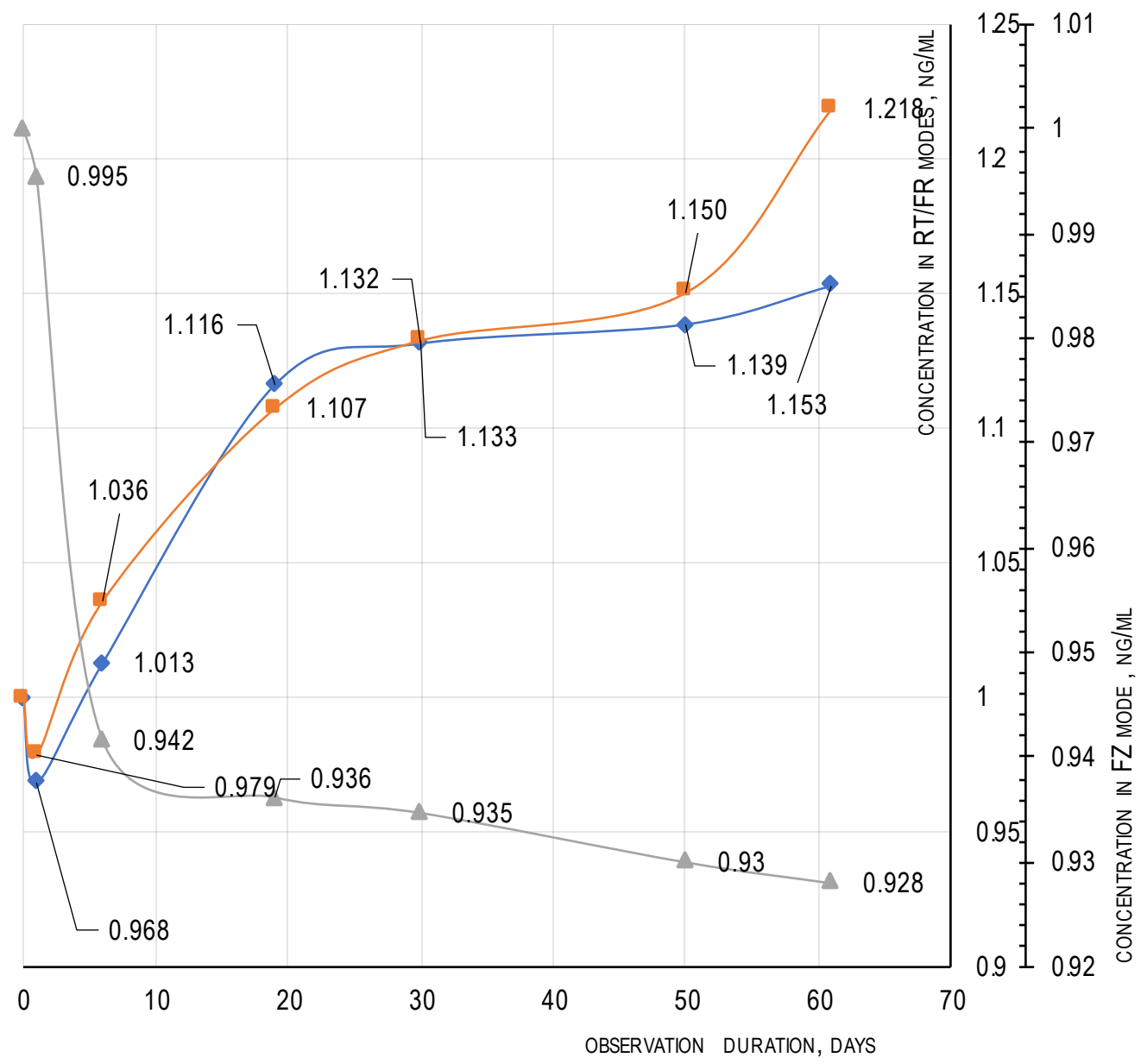

Figure 21. Dynamics of changes in the concentration of HAEE (the initial measurement point is taken as 1 ng/mL) under storage conditions at -20° C (symbol FZ, grey line), at a temperature of 5±2° C (symbol FR, orange lane) and at a temperature of 21±2° C (symbol RT, blue line) in Full-MS scanning mode (by the parent ion with m/z = 528.2329 (z = 1+)). The calculated concentrations, starting from the first day of observation, are normalized to the values of the area and height of the zero observation point in all modes.

The stability of the pure HAEE standard solution in the FR and RT modes meets the acceptance criteria of ±15% [8] for at least 50 days of observation (Table 5). In the RT mode, the corrected concentration over 61 days is 1.153 ng/mL, which slightly exceeds the requirements of the acceptance criteria, while in the FR mode, stability at the level of 15% can be noted over 50 days of observation, where the corrected concentration of HAEE was 1.150 ng/mL; further observation for 61 days shows a concentration value of 1.218 ng/ml. Significantly higher stability of HAEE standard is demonstrated in the FZ mode throughout the entire observation period, where over 30 days of observation, the concentration change was only 7.93% (Table 5 and Figure 21). Taking into account the signal stability in inter-experimental intervals at the level of ±3%, the signal stability in the FZ mode still remains high, since taking into account the integrated measurement error and the degradation effect, the measurement accuracy will not exceed 11%.

Recommendations:

1. It is recommended to use working solutions within no more than 50 days after preparation in an aqueous solution of 0.5% formic acid in case of their long-term storage at a temperature of 5±2° C.
2. It is allowed to store working solutions of HAEE in an aqueous solution of 0.5% formic acid in the form of aliquots of the volume required for work at a temperature of -20° C for a long period of at least 60 days without multiple cycles of freezing and thawing. In this case, the stability of HAEE is noted at a level of at least 92%.

A smooth increase in the concentration of HAEE in the solution in the RT and FR modes is observed throughout the entire observation period and reflects the effects of surface evaporation of the solvent and reverse convection, which were described earlier in this section. The increase in concentration correlates with the change in room temperature, documented in the RT mode (Figure 19A) and the change in the mass of the control solution, which was measured by precise weighing (Figure 20). During the entire observation period, the room temperature was 23.0±1.9° C, the maximum room temperature reached 26.9 ° C, and the minimum room temperature reached 19.9° C. At the same time, up to day 50 of observation, the change in concentration in the FR and RT modes occurred synchronously, and by day 61 of observation, the increase in the concentration of HAEE in the solution in the FR mode sharply accelerated (Figure 21). However, given the high measurement accuracy and signal reproducibility (at least 3%), the obtained data on the asymmetric acceleration of concentration change in the RT and FR modes cannot be considered as artifacts. Indirect confirmation is also the fact that stability at the level of 15% is achieved for the FR mode 10 days earlier compared to the FR mode (Figure 21and Table 5). The researcher cannot find a rational explanation for this phenomenon at the moment. The only assumption can be the presence (appearance) over time in the refrigerator (FR storage conditions) of strong acids or their salts (or anhydrides), which exhibit strong hygroscopic properties, enhancing the forced surface evaporation of the solvent from the vial with HAEE in the FR storage mode , in contrast to the standard in the RT mode, where the main external factor of change is only the room temperature at a fairly stable value of relative humidity (humidity fluctuations ranged from 33% to 39% during the entire observation period (Figure 19B).

7.2. Continuous Storage: Stability of HAEE In the Biological Matrix Environment

The study of any substance in a biological matrix environment has a number of features caused by the interaction of the substance being studied with the components of the selected biological matrix. The very nature of the biological matrix should reflect, first of all, the properties of the biological material in which the diagnostics will be carried out and in which the transportation will be carried out. The degradation of the target substance is determined by the total activity of such a biological matrix, which consists of a number of effects, including biochemical interaction determined by the activity of enzymes of the biological matrix. Biochemical interaction can lead to the transformation of the substance being studied by either its conversion into derivatives or complete or partial degradation. Losses, in this case, are limited by the time intervals of collection, storage and transportation of the biological matrix and can be slowed down by preservatives and inhibitors of varying specificity. On the other hand, specific and non-specific interactions with the components of the biological matrix can lead to imaginary losses of the target substance. In this case, losses may be due to co-precipitation of the test substance with the bound component of the biological matrix, thus reducing the concentration of the free substance in the matrix. It is not always possible to release the bound ligand from the complex: the interaction can be strong and non-specific, which significantly complicates the development of approaches to dissociation of the complex. It should be noted that caution is required in such cases, since the use of chemical methods for dissociation of complexes can lead to irreparable side effects, in particular, the initiation or catalysis of reactions of undesirable transformation of the test substance. Thus, along with the release from the complex, a number of by-products of third-party chemical reactions can be obtained, which significantly complicates both the interpretation of the final results and further isolation (extraction), since the resulting set of compounds as a result of such reactions can differ to a greater extent in their physicochemical properties and not be isolated in parallel. Thus, the determination of the conditions of isolation, storage and transportation

requires a careful approach to the choice of the nature of the biological matrix, based on its properties and the properties of the target substance itself.

In our study, the choice of biological matrix is limited to two types: serum or plasma. Preliminary studies to determine the concentration were conducted using both human serum and plasma. However, the properties of these biological matrices are not the same and require specific approaches depending on the goals and objectives of the analysis. The biochemical properties of the biological matrix can significantly affect the degree of extraction of the target substance, its stability in the environment of proteins, trace elements and low molecular weight compounds, the half-life of the target substance, and determination of the true concentration. The latter, determination of the true concentration, is especially important in the context of the stability of the substance in the environment of the components of the biological matrix. This is due to the fact that the internal standard introduced into the biological matrix may be characterized by apparent stability and apparent concentration due to the absence of direct influence of matrix components on it during preanalytical sample preparation, while the signal of the endogenous target component may be either completely absent or contained in very small apparent quantities due to the influence of matrix properties at the stage of preanalytical preparation. Moreover, the properties of the same type of biological matrix may differ and have different analytical purposes depending on the components of preanalytical preparation. Thus, plasma obtained with EDTA as an anticoagulant is intended for hematological studies (qualitative and quantitative blood composition), as well as for molecular genetic studies and determination of viral load. The use of EDTA in combination with aprotinin helps to stabilize unstable and problematic analytes, such as some hormones. The combination of potassium EDTA (trisubstituted), sodium fluoride and sodium oxalate help maintain sample stability for up to 48 hours at room temperature. The combination of both sodium citrate and dextrose helps maintain sample stability at a temperature of +2 °C…+6 °C for a fairly long time: from 21 to 35 days of storage. Finally, the use of heparin as a dominant anticoagulant is necessary for such studies as blood electrolyte composition, blood gas composition and determination of blood alcohol content.

The stability of a substance, both endogenous and exogenous, under long-term storage conditions requires conducting a study at various temperature conditions. Obviously, the experimental conditions of normal temperature, i.e. within 20±2 °C, are of little importance, since the storage of biological matrices under such conditions is simply unacceptable or limited to a very short period of time, such as when collecting blood in test tubes with sodium fluoride, which allows storing blood plasma for up to 48 hours at room temperature. The tasks of researchers include, as a rule, determining the stability of long-term storage at temperatures of -20 °C and below. In turn, there is an opinion that determining the stability at lower temperatures, i.e. up to -80 °C or below, does not have much practical value and is not even recommended, especially if sufficient stability has been demonstrated at a temperature of -20 °C. Nevertheless, the conditions of -20 °C and -80 °C, for example, can have striking differences in stability, especially when it comes to complex biological matrices [9].

It is obvious that low temperatures will proportionally slow down any chemical and biochemical reactions in the biological matrix, according to the well-known kinetic law of Arrhenius. However, the researcher must take into account that at lower temperatures protein molecules can undergo deeper and stronger denaturation with partial loss of their native three-dimensional structure. In turn, this can have an unexpected effect on the subsequent extraction capacity of the target molecule from the biological matrix with proteins, and the effect will be more pronounced if the low-molecular substance is a partner or ligand of some large (protein) molecule. It is significant that in such a case there is a large degree of uncertainty in the effect, since on the one hand, the low-molecular substance can be entrained inside the core of the denatured molecule, which complicates its extraction to the point of making it impossible to extract using organic solvents, and on the other hand, conformational changes in the protein molecule can lead to a complete loss of the ability to bind the low-molecular substance, which, on the contrary, significantly facilitates its further extraction from the biological matrix and, consequently, increases the degree of extraction [10] .

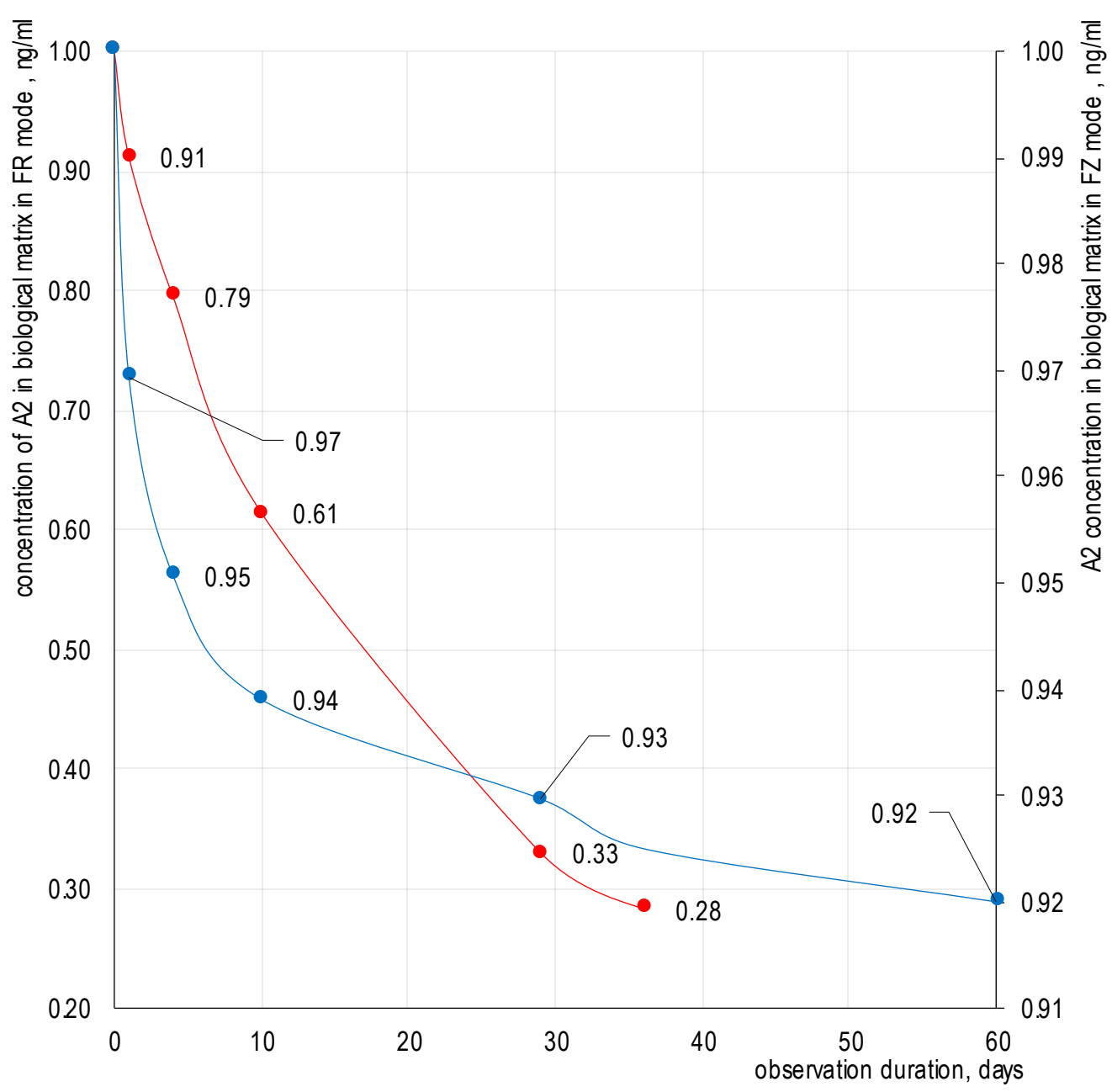

The stability of a substance under low-temperature storage conditions involves studying the cyclic defrosting mode and the long-term interval storage mode, i.e., when the matrix is defrosted once after specified storage time intervals. However, there is a reasonable opinion that a parallel study in such storage options is also of little value, since in the case of high stability of a substance after several defrosting and thawing cycles of the biological matrix containing it, a high probability of stability is expected with an identical shelf life of a biological sample, but with a single defrosting. It should be noted that in such studies, in parallel with the physical and chemical stability of the target substance and biological matrix, an assessment of the stability of the extraction is carried out. This is a non-trivial task and many researchers believe that such an assessment cannot be objective for many reasons, including heterogeneity (individual differences) of complex biological matrices, as well as the dependence of the extraction capacity not only on stability over time during storage, but also on the time of collection of the biological sample (matrix), the general condition of the patient, the technology of sample collection, transportation times, etc. A fair assessment of stability is post-preparation, that is, the stability of the extract itself under various temperature conditions.

Figure 22. Changes in the concentration of HAEE (the initial measurement point was taken as 1 ng/ml) in a biological matrix (human blood plasma EDTA-2K⁺) under storage conditions at -20 °C (symbol FZ, blue line) and at a temperature of 5±2°C (symbol FR, red line) in the full MS scanning mode (by the parent ion with m/z = 528.2329 (z = 1+)). Observations under FR conditions were stopped on the 36th day after the start of the experiment. The calculated concentrations, starting from the first day of observation, were normalized to the values of the area and the height of the zero observation point in all modes.

In our study, we evaluated the stability at temperatures of -20°C (**FZ**) and 5±2°C (**FR**) with different storage periods. Human blood plasma with EDTA-2K⁺ was selected as the biological matrix. The working sample of the biological matrix was prepared by combining equal volumes of four biological samples taken from different patients. Internal labeled HAEE standard was added to the combined biological matrix to a final concentration of 1 ng/mL. Next, 120 µL aliquots of the combined matrix were prepared for the study at -20° C (**FZ**) and 5±2°C (**FR**). The zero-observation point, i.e. the study of the biological matrix immediately after preparation with the internal standard, was taken as the reference and used for normalization of all further observations (Table 6). Despite the ambiguity of recommendations on the conditions for defrosting a biological sample, we always carried out this procedure in the same way at room temperature, since according to some studies, defrosting at moderately low temperatures (i.e. within +4…+8° C) is not optimal due to the longer exposure of the components of the biological matrix to ice crystals.

Table 6. Changes in the signal **of labeled HAEE standard in a biological matrix** (human blood plasma EDTA-2K⁺) by area (A) and height (H) of the chromatographic peak in the full MS scanning (Full-MS) and high-resolution SRM (HR-SRM) modes during 36 days of observation at a temperature of 5±2°C (symbol FR) and during 60 days of observation at a temperature of -20°C (symbol FZ). The repeatability of the measured concentration was determined by the ratio of the calculated concentration by height to the calculated concentration by the area of the chromatographic peak. Observations of labeled HAEE standard in the FR mode were interrupted after 36 days due to the degree of degradation reaching a level significantly exceeding the stability deviation standards of the substance under the specified storage conditions.

Chromatographic peak height	Duration of observation, days		0	1	4	10	36	No data
	FR	R01	810365	714206	640154	487125	223447	
		R02	809461	724439	629818	481330	221671	
		R03	742035	714146	606859	482316	222551	
	Average value			717597	625610	483590	222556	
	Relative standard deviation		4.98%	0.83%	2.72%	0.64%	0.40%	
	Calculated concentration, ng/ml		1	0.911	0.795	0.614	0.283	
	Duration of observation, days		0	1	5	10	29	60
	FZ	R01	1247197	1228871	1213654	1244515	1228874	1196671
		R02	1213967	1221354	1218937	1194781	1264794	1195412
		R03	1244379	1219617	1215664	1181709	1267635	1198934
	Average value		1235181	1223281	1216085	1207002	1253768	1197006
	Relative standard deviation		1.49%	0.40%	0.22%	2.75%	1.72%	0.15%
	Calculated concentration, ng/ml		1	0.99	0.985	0.977	1.015	0.969
Chromatographic peak area	Duration of observation, days		0	1	4	10	36	No data
	FR	R01	52326	46637	42576	31162	14118	
		R02	53802	46392	40550	32754	14325	
		R03	46665	46393	39152	32992	14508	
	Average value			46474	40759	32303	14317	
	Relative standard deviation		7.40%	0.30%	4.22%	3.08%	1.36%	
	Calculated concentration, ng/ml		1	0.912	0.8	0.634	0.281	
	Duration of observation, days		0	1	5	10	29	60
	FZ	R01	84741	82961	80091	79984	79683	78641
		R02	85283	82885	81241	79856	78610	78592
		R03	85128	81477	81237	79759	78919	78334
	Average value			82441	80856	79866	79071	78552
	Relative standard deviation		0.33%	1.01%	0.82%	0.14%	0.70%	0.21%
	Calculated concentration, ng/ml		1	0.969	0.951	0.939	0.93	0.923
Convergence A/H		FR	100.00%	99.89%	99.29%	96.85%	100.56%	No data
		FZ	100.00%	102.17%	103.56%	104.06%	109.18%	104.97%

The results of observations of the degradation kinetics (stability) of labeled HAEE in the biological matrix medium under FZ and FR conditions are presented in Table 6 and Figure 22. Observations of HAEE in the biological matrix medium in the FR storage mode were interrupted on the 36th day, since by this time the residual amount of the substance in the medium was only 36% of the initial concentration (Figure 22). Taking into account the requirements for stability of no more than ±15%, the most optimal shelf life of the biological matrix with labeled HAEE with an initial concentration of 1.00 ng/ml is 24 hours (one day; Figure 22and Table 6), since after four days the loss of the substance due to natural degradation in the medium is 20% or more (Table 6). Degradation of labeled HAEE in the biological matrix medium at temperature FR is characterized by the properties of nonlinear power regression (Figure 22), and half of the initial amount degrades within a period (calculated value) of 8.7 days. Under storage conditions of FZ, the substance exhibits satisfactory stability in the biological matrix throughout the entire observation period (60 days; Figure 22). During the observation period, quantitative losses of the substance in the biological matrix medium at temperature FZ amounted to no more than 7% (or no more than 2.3% by the height of the chromatographic peak, and taking into account the convergence of the results by the area and height of the chromatographic peak in the FZ measurement mode , the reduced (weighted) losses amount to no more than 4.1%; Table 6). At the same time, starting from the first day of observation, the degradation kinetics of substance A does not exhibit active properties and proceeds in a monotonic mode (Figure 22). The calculated shelf life of the biological matrix before reaching losses of half the initial amount does not fit into the framework of rational storage periods. The estimated shelf life of a biological sample with endogenous HAEE substance or labeled HAEE standard until the maximum permissible quantitative loss of 15% is reached, i.e. until the concentration reaches 0.85 ng/ml, is more than 50 years, which also goes beyond the rational shelf life.

Recommendations:

1. The shelf life of HAEE in the biological matrix environment or a sample of the biological matrix with endogenous HAEE substance under FR conditions (at a temperature of 5±2°C) is no more than one day (24 hours). In exceptional cases, storage of the matrix with endogenous HAEE or labeled HAEE standard under FR conditions is permitted for up to four days, taking into account possible quantitative losses of up to 20% of the initial apparent concentration.
2. Long-term storage of the biological matrix with endogenous HAEE or labeled HAEE internal standard in the FZ mode (at a temperature of -20±2°C) is safe throughout the entire observation period. Storage of the biological matrix with either of substance (endogenous or/and labeled HAEE) at the specified temperature is allowed for a period of at least 60 days with possible maximum quantitative losses of up to 6%. The estimated continuous shelf life of a biological sample with HAEE at a temperature of -20±2°C until quantitative losses of 15% of the initial concentration are achieved is more than 3 years.

7.3. Interval (cyclic) Stability of HAEE In Working Solution and Biological Matrix at Low Temperatures

Interval or cyclic stability is determined by the stability of the desired substance under conditions of continuous storage of the working solution or biological matrix for a long time with cyclic freezing and thawing. In our study, interval stability was determined under conditions of long-term storage of aliquots of the working solution of HAEE at a final concentration of 1 ng/ml or a biological matrix with internal HAEE to a final concentration of 1 ng/ml at a temperature of -20°C (FZ). In both cases, the volume of the prepared aliquots was 1200 µl (1.2 ml), which underwent several freeze-thaw cycles throughout the study. For the study, we collected 100 µl after each complete thawing cycle of the sample and prepared the sample in accordance with the recommendations outlined in Section no. 5.

The study of interval storage under low temperature conditions is determined by the need to establish time limits during which a biological sample can be stored after collection of biological material until it is sent to the analytical laboratory without causing significant damage to the integrity of the components of the biological matrix and minimizing the loss of the desired analyte in the biological matrix. Interval storage, as has already been previously indicated, involves long-term storage of the sample under established experimental conditions and, accordingly, provides for cyclic changes in storage conditions throughout the entire observation period. Thus, upon achieving high stability indicators of HAEE in the biological matrix over a long continuous period, these sample storage conditions are also a projection of the optimal conditions for transporting the biological material from the collection site to the site of analytical preparation and measurements.

In accordance with the recommendations *of the International Consortium for Harmonization of Bioanalytical Research* [11], stability studies should be continued until the signal deviation does not exceed 20% in a series or series of studies, and the deviations should be due specifically to storage conditions and be systematic in a series of observations.

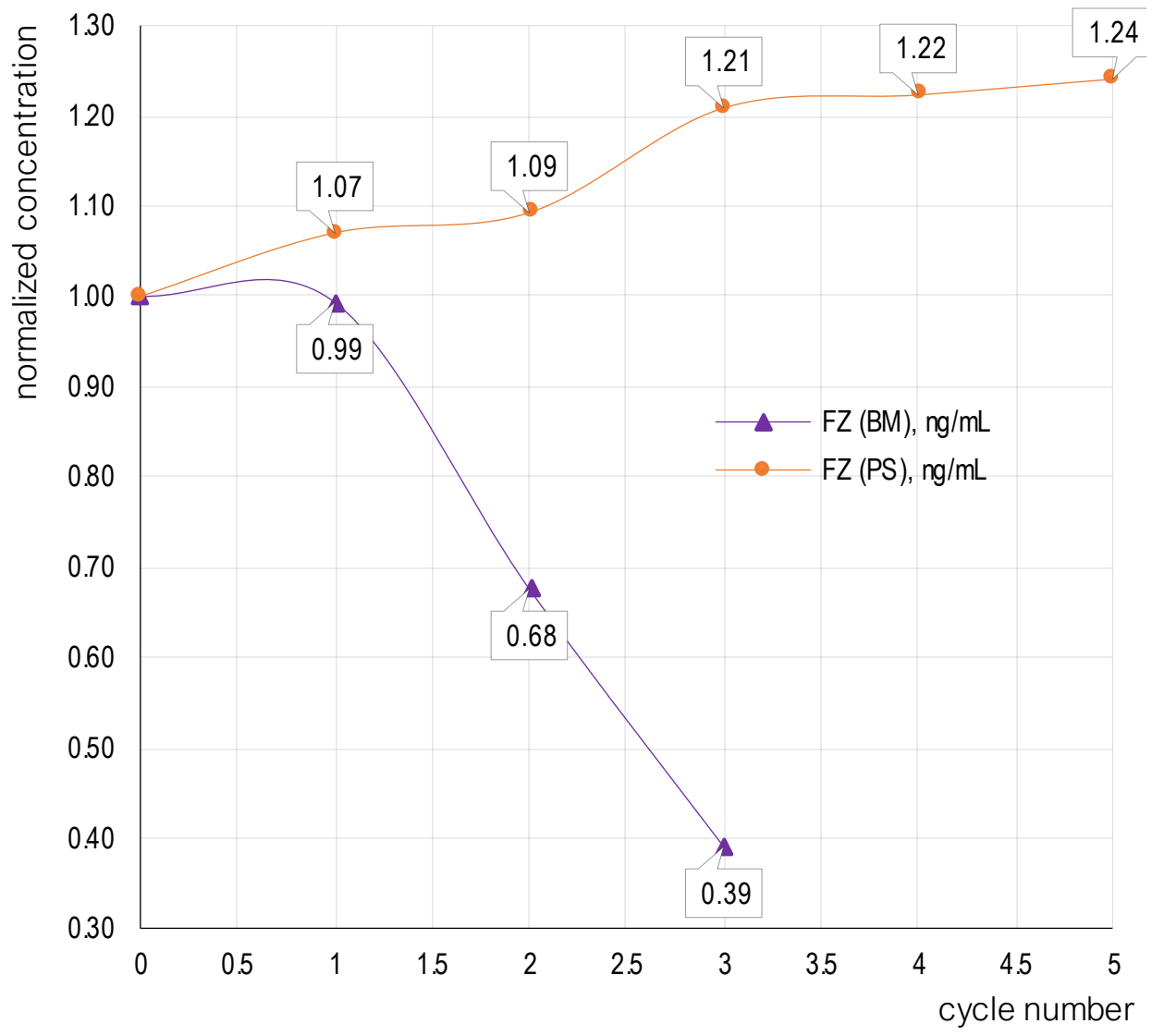

Figure 23. Graph of changes in the concentration of HAEE in pure solution (orange line) and in the biological matrix (purple line) during five and three cycles of freezing at -20°C and thawing at room temperature. The initial observation point corresponds to a concentration of 1 ng/ml of HAEE.

The study of interval (cyclic) stability of HAEE in a biological matrix (EDTA-2K+ human blood plasma) was conducted over three full cycles of freezing at a temperature of -20°C and thawing for 10 days, after which the study of stability in the biological matrix was stopped in the form of a decrease in the concentration normalized to the zero point of observations to a critically low level (Figure 23). Over time, by the third cycle, the residual concentration of HAEE in the biological matrix was only 0.39 ng/ml, i.e. no more than 39% of the initial concentration at the beginning of the observations (Table 7. Summary data on changes in the concentration of HAEE in a 0.5% formic acid solution and in a biological matrix (EDTA-2K+ human blood plasma) during five and three full cycles of freezing at -20°**.**

Table 7. Summary data on changes in the concentration of HAEE in a 0.5% formic acid solution and in a biological matrix (EDTA-2K+ human blood plasma) during five and three full cycles of freezing at -20°C and thawing at room temperature. The initial observation point corresponds to the reference concentration of 1 ng/ml of HAEE. The initial data are given for the height of the chromatographic peak.

Cycle number		0	1	2	3	4	5
FZ in solution	R01	643927	682374	693511	759032	774581	800561
	R02	637972	681004	696032	761927	778111	789726
	R03	622900	675644	691941	781523	777154	773028
Average value		634933	679674	693828	767494	776615	787772
Relative standard deviation		1.71%	0.52%	0.30%	1.59%	0.24%	1.76%
Calculated concentration, ng/ml		1	1.0705	1.0928	1.2088	1.2231	1.2407
FZ in matrix	R01	824840	786212	566182	311728	No data	
	R02	813437	825056	520328	322739		
	R03	782232	793215	549573	310335		
Average value		806836.5	801494	545361	314934		
Relative standard deviation		2.73%	2.58%	4.26%	2.16%		
Calculated concentration, ng/ml		1	0.9934	0.6759	0.39033		

The relative standard deviation of the signal (area and height of the chromatographic peak) in a series of three consecutive measurements at each observation point does not exceed 5% (Table 7), and the decrease in the calculated concentration is systematic throughout the entire observation period (Figure 23). Based on the data obtained, no more than one full cycle of freezing and thawing can be recommended, since in this case the decrease in concentration does not exceed 1% of the initial reference concentration of HAEE at the beginning of the observations.

Recommendation:

Thus, it is possible to recommend freezing the biological sample immediately after collection and transporting such a sample at a temperature of -20°C strictly without thawing, since in this case the results obtained in the analytical center will not differ significantly from the true concentrations of HAEE in the biological sample. Repeated freezing and thawing of the biological sample significantly affect the decrease in the concentration of HAEE in the biological matrix, which is probably associated with the entrainment of macromolecular complexes and proteins that denature during changes in temperature conditions.

In contrast to the biological matrix, a smooth increase in the concentration of HAEE is observed in the solution over at least five complete freeze-thaw cycles (Figure 23 and Table 7). Throughout the observation period, high stability of the HAEE signal can be noted in a series of three technical replicates, where the relative standard deviation does not exceed 2% (Table 7). The largest relative increase in concentration can be noted between the second and third observation cycles (an increase in concentration from 1.09 ng/ml to 1.21 ng/ml), corresponding to the 6th (six) and 19th days of observation, i.e. the interval between these two observations was two weeks. By the end of the fifth observation cycle (50th day of observation), the calculated concentration of HAEE in the solution according to the measurement results was 1.24 ng/ml, which is 24% higher than the initial concentration (1.00 ng/ml). The results of the study, which seem paradoxical at first glance, are related to the phenomenon of sample freezing during long-term storage at low temperatures, during which a physical process of crystallization of water molecules from the solution occurs upon contact with water molecules of the gas phase (water vapor), soluble in the liquid phase as the temperature decreases. Thus, the measured concentration is an imaginary concentration of HAEE from the point of view of analytical approaches. In this case, it can be assumed that the degradation rate of HAEE in a pure solution of 0.5% formic acid is significantly lower than the freezing rate of the solution, however, final conclusions can be made after additional research on solutions with a series of different concentrations and upon reaching the equilibrium state of the concentration change curve, caused by the stoichiometric equilibrium between the freezing rate of the solution and the rate of retention of solvent molecules by the hydration shell of HAEE and formic acid molecules.

<u>Recommendation:</u>

In this case, it is necessary to prepare stock or working solutions in small aliquots and regulate long-term storage at low temperatures without defrosting and refreezing in order to avoid obtaining false-negative results for measuring endogenous HAEE in a biological sample, since the concentration of labeled HAEE standard (ISTD), when used as an internal standard for measurement will increase with each defrosting and refreezing cycle.

Section 8. Carryover

The effect of mass transfer, or transfer of the analyte, during the analysis from one sample to another, is a frequent phenomenon in analytical chemistry and requires careful study. Horizontal mass transfer can be caused by a number of technical, physicochemical factors, properties of solvents, stationary carrier or a combination of these factors in various combinations. Horizontal mass transfer can be especially critical for decision-making in the case of research in the low concentration region of the quantitative range under study. If the effect of transfer is too large and exceeds the previously specified limits of permissible deviation of the relative response or absolute area and height of the chromatographic peak in a series of successive technical repetitions, the researcher must adjust the calculated values of the detection limits and the limit of quantification. Often, the effect of horizontal mass transfer can be clearly expressed even at the stage of constructing a calibration curve, when the linear range of concentrations can have a fairly large dispersion in the low concentration region. However, the effect may not be noticeable if the researcher uses the relative response of the signal to the internal standard, since calculating the concentration from the relative response is close to applying a weighting factor to the calibration curve over a wide concentration range.

Horizontal mass transfer can also be caused by the properties of the stationary phase of the chromatographic system. For example, incorrectly selected conditions of the elution gradient or physicochemical properties of the carrier can retain the substance under study or components of the biological matrix that non-specifically bind the target substance for a long time. In this case, it is possible to increase the washing time of the stationary phase in combination with the use of mobile phases that differ in properties from those used to create the elution gradient (for example, washing in methanol, isopropanol, octane, or their combination with an organic mobile phase of the elution gradient). Such an approach, of course, leads to a significant increase in the total analysis time, since it requires additional time not only for washing the stationary phase, but also for further equilibration in the initial conditions of the elution gradient before applying the next sample, so it is inappropriate to follow such an approach from the point of view of time and money.

On the other hand, the mass transfer effect may be caused by incorrect injector settings. Correct operation of the injector can be indirectly determined by the reproducibility of the height, area and width of the chromatographic peak at the base. An additional indicator is the pressure drop when switching the system, which will be inconsistent if there is a hidden dead volume in the bypass valve system or between the needle and the injector seat.

Previously, in our study, no significant indirect or direct signs of the mass transfer effect from sample to sample were noted. Although it must be recognized that such signs could be leveled by the use of an internal standard, the use of which, as was said earlier, is capable of hiding the signs of horizontal mass transfer due to the similarity to the weight factor. Nevertheless, in the process of studying the properties of the calibration curve, we noticed the effect of a small isotopic interference, but the nature of this effect is completely different from the nature of the horizontal mass transfer effect.

In the simplest case, the carryover effect is determined by calculating the additive area or peak height that exceeds the standard deviation in several consecutive measurements, with the additive value typically being detected in the direction from the highest to the lowest concentration of the measurement. Trace evidence of such a mass carryover effect can therefore also be detected in an empty blank sample, where the signal level may exceed the calculated LOB level by more than 1.647σ.

In our case, the study of the mass transfer effect is of particular importance, since the expected concentrations in the norm and pathology lie significantly below the upper and middle levels of the calibration curve. Thus, the mass transfer effect can become critically important for making a decision on stratification and qualification of the obtained results, especially if the measured values are at the border of the lower limit of the norm. In this regard, we developed a special design of the experiment to study the mass transfer effect. In the work, we used standards of low (0.125 ng/ml), medium (0.500 ng/ml) and high (1.000 ng/ml) concentration, prepared on the day of the experiment and designated hereinafter as ESTD - A, ESTD - B and ESTD - C, respectively. The first step involves determining the reference signal from each standard (Figure 24, Step 1) with a description of the standard deviation, the relative response, and the deviation of the calculated response from the theoretical one in terms of the height and area of the chromatographic peak in five consecutive technical replicates for each standard. The relative response was taken as the value of the dilution ratio, or relative concentration, of the standards in the following order: RR = 8 for calculating ESTD - C/ESTD - A, RR = 4 for calculating ESTD - C/ESTD - B , RR = 2 for calculating ESTD - A/ESTD - B ; these values are a theoretical guideline, and their constancy reflects the stability of the initial dilution, as well as the stability of the measurement in different assay schemes. Four assay schemes were used to study the effect of horizontal mass transfer (Figure 24, Step 1.1 and Step 1.2). The direct scheme (Step 1.1.1), in which the standards are measured in the direction from lower to higher concentration in triplets in three technical repetitions, that is, according to the scheme (A →B →C) + (A →B →C) + (A →B →C) or corresponds to the expression [A →B →C] × 3. The reciprocal scheme (Step 1.1.2) provides for the reverse order of concentration measurement, that is, from a higher value to meshes, or corresponds to the expression [C →B →A] × 3. The third scheme is a random measurement (Step 1.1.3) in triplets, where the position of each standard is not determined and can have any order in each triplet, but cannot be duplicated within one triplet, or corresponds to the expression [X, Y, Z] × 3. The three previous schemes of analysis are continuous, while the last, fourth, scheme of analysis is interval and represents the measurement of standards with the obligatory inclusion of a blank sample between each measurement. The interval measurement (Step 1.2) does not strictly regulate the scheme of the measurement order, since with any of them in the blank sample it is possible to detect a signal above the LOB level for any measured sample, if it is caused by the effect of horizontal mass transfer. In our study, we chose the classical, direct scheme of interval measurement (Step 1.2.1), or the corresponding expression [A →(bA) →B →(bB) →C →(bC)] × 3. The experimental design is presented in Figure 24.

It should be noted that the relative response, as well as the differential of the relative response within a measurement group and with respect to the reference signal for continuous direct, random and interval direct measurements were estimated as indicated above, namely RR = 8 for calculating ESTD - C/ESTD - A, RR = 4 for calculating ESTD - C/ESTD - B, RR = 2 for calculating ESTD - A/ESTD - B. For the continuous reciprocal study, the reciprocal values corresponding to RR (rA/rC) = 0.125, RR (rB/rC) = 0.250 and RR (rB/rC) = 0.500 were used. However, the use of direct or reciprocal values in the calculations does not change the essence of the final results and does not affect their interpretation. The results of the study were assessed based on the values of the differential relative response in each analysis scheme, the empirical additive effect, and the calculated (theoretical maximum permissible) integral additive effect for each standard in each analysis scheme.

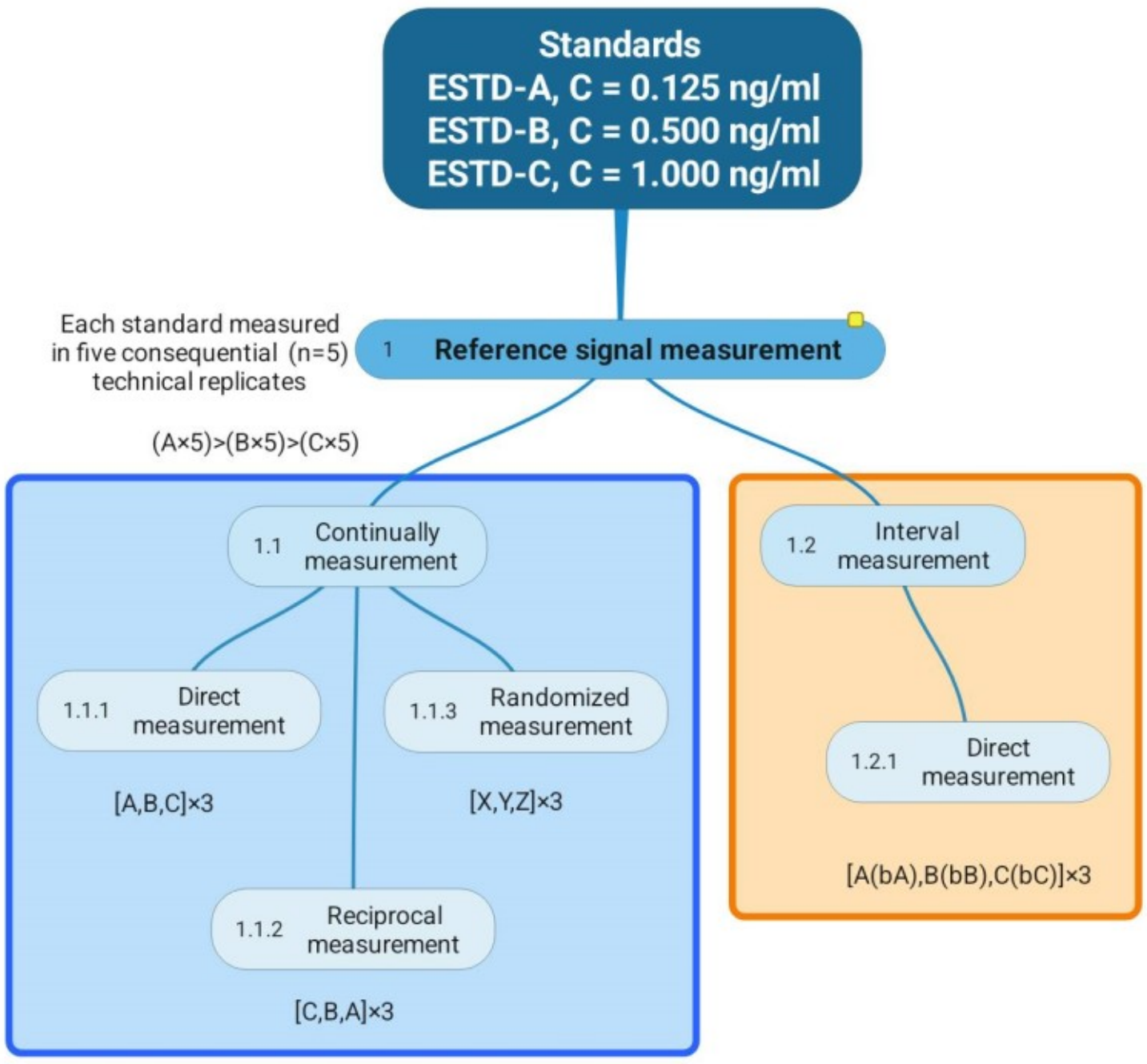

Figure 24. General scheme of the mass transfer effect study design. The reference signal measurements (chromatographic peak area and height) are carried out using three standards (ESTD - A = 0.125 ng/mL, ESTD - B = 0.500 ng/mL, and ESTD - C = 1.000 ng/mL) in five consecutive technical replicates for each standard (**Step 1**). Then, continuous (**Step 1.1**) or interval (**Step 1.2**) measurements are carried out. The continuous measurements include direct (**Step 1.1.1**), reciprocal (**Step 1.1.2**), and random (**Step 1.1.3**) analysis schemes. In each case, the measurements are made in triplets of three technical replicates for each standard in the order established by the analysis scheme. For interval (**Step 1.2**) measurements, only a direct analysis scheme (**Step 1.2.1**) is provided, which involves measuring each standard sequentially in the direction from lower to higher concentration with the inclusion of a blank sample between each measurement, regardless of the position of the standard.

Within each measurement series, the average signal value was calculated for the area and height of the chromatographic peak, as well as the standard deviation and the coefficient of relative variation of the signal within the series. The relative response and its differential to the expected relative response corresponding to RR = 8 for ESTD - C / ESTD - A , RR = 4 for ESTD - C / ESTD - B and RR = 2 for ESTD - A / ESTD - B in direct, direct interval and random measurements and the corresponding RR (rA/rC) = 0.125, RR (rB/rC) = 0.250 and RR (rB/rC) = 0.500 for continuous reciprocal measurement were calculated for each individual measurement in relation to the desired fixative within the corresponding measurement triplet. The additive effect, i.e. the coefficient of horizontal mass transfer, was calculated in each case of measurements in relation to the signal of the corresponding fixed channel in a series of reference measurements, as the difference between the differential of the relative signal within the triplet and the differential of the signal of the reference measurements. By definition, the additive effect cannot exceed the value of the standard deviation of the signal or the empirical value of the relative response within a series of reference measurements, but can take positive and negative values, where positive values of the additive signal can be a sign of horizontal mass transfer in a series of successive measurements. This effect can be expressed most clearly when using a reverse measurement scheme, and a random measurement scheme allows measurements to be carried out in a situation maximally close to a routine series of multiple measurements of samples with an uncertain concentration.

The expected integral additive effect reflects the maximum possible horizontal mass transfer during successive measurements taking into account the additive effect within each measurement series. The expected integral mass transfer effect allows for the probability of the additive effect and the error of the first kind, but with a probability no greater than the specified calculated value. In view of the fact that the horizontal mass transfer effect consists of a number of circumstances and factors with a large share of uncertainty, such as the operation of the injector, the properties of the chromatographic carrier, the properties of the capillaries, the individual properties of the biological matrix and its interaction with the desired substance, the speed of sample collection and injection, the speed and time of equilibration of the chromatographic system in the mobile phase and many other factors, it is advisable to use the harmonic mean when calculating the integral additive effect, since the harmonic mean, unlike the arithmetic mean and mathematical expectation, is a more stable parameter to random variables, and in the study of the transfer effect any additive effect is considered as a random variable with a large uncertainty.

Table 8. Measurement data of the reference signal of HAEE (m/z = 528.2339). The reference signal of the area and height of the chromatographic signal is presented for HAEE at a concentration of 0.125 ng/mL (ESTD - A), 0.500 ng/mL (ESTD - B) and 1.000 ng/mL (ESTD - C). The signal measurements for each fixanal were carried out in five technical replicates. To calculate the average signal value, standard deviation and relative covariance of the signal, none of the technical replicates were excluded. The relative response calculation was performed for the ESTD - C / ESTD - A signal ratio (the expected relative response is 8 units), for the ESTD - B / ESTD - C ratio (the expected relative response is 4 units) and for the ESTD - C / ESTD - B ratio (the expected relative response is 2 units).

Standard	Concentration	Peak height	Peak area	Average peak height	Average peak area	Standard deviation in height	Standard deviation by area	Relative response expected	Relative response calculated	Relative response differential
ESTD-A	0.125	63388	3920	63270	3890	0.90%	2.09%	8	8.871	10.88%
		62716	3749							
		63240	3922							
		62844	3904							
		64160	3956							
ESTD-B	0.5	267296	17475	260518	16652	3.57%	3.26%	4	4.199	4.98%
		263163	16447							
		244233	16020							
		262487	16483							
		265409	16836							
ESTD-C	1	548055	34847	548695	35281	1.14%	2.17%	2	2.112	5.62%
		541455	34654							
		558422	36362							
		549642	35815							
		545900	34728							

Table 9. Data of signal measurements of HAEE at concentrations of 0.125 ng/ml (ESTD - A), 0.500 ng/ml (ESTD - B) and 1.000 ng/ml (ESTD - C) in **direct measurement sequence** from lower concentration to higher concentration corresponding to scheme [A , B , C] × 3. Measurements were performed in triplets of three technical replicates. Calculation of average area and height of chromatographic peak corresponding to HAEE was performed for all three technical replicates in series of direct measurements. Calculation of relative response and differential of relative response were performed within series of direct measurements for each triplet; calculation of additive effect of horizontal mass transfer was performed in relation to reference signal within each triplet for the entire series of direct signal measurements. The calculation of the expected integral effect was carried out for each triplet within a series of measurements using the empirical relative response (calculated) in a series of measurements of the reference signal.

Standard	Concentration	Peak height	Peak area	Average height			Relative response expected	Relative response calculated	Relative response differential	Additive effect	Expected integral additive effect	
				ESTD-A	ESTD-B	ESTD-C						
ESTD-A1	0.125	68980	4174				8	8.268	3.35%	-7.54%		
ESTD-B1	0.5	275045	18468	68175±1.55%	273976±0.6%	553346±0.54%	4	4.206	5.15%	0.17%	6.75%	ESTD-A
ESTD-C1	1	552638	35579				2	1.968	1.61%	-4.02%		
ESTD-A2	0.125	68566	4221				8	8.18	2.25%	-8.64%		
ESTD-B2	0.5	272089	16617	Average area			4	3.953	1.19%	-3.79%	3.52%	ESTD-B
ESTD-C2	1	550749	35150				2	2.07	3.49%	-2.14%		
ESTD-A3	0.125	66980	4118				8	8.432	5.40%	-5.49%		
ESTD-B3	0.5	274795	17519	4171±1.24%	17535±5.28%	35316±0.65%	4	4.178	4.46%	-0.52%	1.91%	ESTD-C
ESTD-C3	1	556651	35220				2	2.018	0.90%	-4.72%		

Table 10. Data of signal measurements of HAEE at concentrations of 0.125 ng/ml (ESTD - A), 0.500 ng/ml (ESTD - B) and 1.000 ng/ml (ESTD - C) in **reciprocal (reverse) sequence of measurements** from higher concentration to lower concentration corresponding to the scheme [C , B , A] × 3. Measurements were carried out in triplets of three technical repetitions. Calculation of the average value of area and height of the chromatographic peak corresponding to HAEE was carried out for all three technical repetitions in a series of reverse measurements. Calculation of the relative response and differential of the relative response were carried out within a series of reverse measurements for each triplet; calculation of the additive effect of horizontal mass transfer was carried out in relation to the reference signal within each triplet over the entire series of reverse signal measurements. The calculation of the expected integral effect was carried out for each triplet within a series of inverse measurements using the empirical relative response (calculated) in a series of measurements of the reference signal.

Standard	Concentration	Peak height	Peak area	Average height			Relative response expected	Relative response calculated	Relative response differential	Additive effect	Expected integral additive effect	
				ESTD-C	ESTD-B	ESTD-A						
ESTD-C1	1	559671	35088				0.125	0.134	7.23%	1.61%		
ESTD-B1	0.5	268233	17324	559538±1.44%	273630±1.72%	71867±3.8%	0.5	0.486	2.70%	-2.28%	0.41%	ESTD-A
ESTD-A1	0.125	74874	4712				0.25	0.276	10.23%	-0.66%		
ESTD-C2	1	551402	35215				0.125	0.127	1.55%	-4.07%		
ESTD-B2	0.5	276956	17145	Average area			0.5	0.495	1.09%	-3.89%	5.69%	ESTD-B
ESTD-A2	0.125	69545	4499				0.25	0.257	2.70%	-8.18%		
ESTD-C3	1	567542	37628				0.125	0.121	3.49%	-2.13%		
ESTD-B3	0.5	275702	17626	35977±3.98%	17365±1.4%	4523±3.93%	0.5	0.477	4.58%	-0.40%	10.84%	ESTD-C
ESTD-A3	0.125	71181	4359				0.25	0.253	1.10%	-9.79%		

Table 11. Data of signal measurements of HAEE at concentrations of 0.125 ng/ml (ESTD - A), 0.500 ng/ml (ESTD - B) and 1.000 ng/ml (ESTD - C) in **a random sequence of measurements** corresponding to the scheme [X , Y , Z] × 3. Measurements were carried out in triplets of three technical repetitions. Calculation of the average value of the area and height of the chromatographic peak corresponding to HAEE was carried out for all three technical repetitions in a series of random measurements. Calculation of the relative response and the differential of the relative response were carried out within a series of random measurements for each triplet; calculation of the additive effect of horizontal mass transfer was carried out in relation to the reference signal within each triplet over the entire series of random signal measurements. The calculation of the expected integral effect was carried out for each triplet within a series of random measurements using the empirical relative response (calculated) in a series of measurements of the reference signal.

Standard	Concentration	Peak height	Peak area	Average height			Relative response expected	Relative response calculated	Relative response differential	Additive effect	Expected integral additive effect	
				ESTD-A	ESTD-B	ESTD-C						
ESTD-B1	0.5	282277	18423				4	4.249	6.23%	1.25%		
ESTD-A1	0.125	70219	4114	70709±0.99%	280440±2.48%	575202±1.12%	8	8.973	12.16%	1.28%	1.17%	ESTD-A
ESTD-C1	1	582614	39695				2	2.109	5.47%	-0.16%		
ESTD-A2	0.125	70397	4121				8	8.889	11.12%	0.23%		
ESTD-B2	0.5	286291	18425	Average area			4	4.269	6.72%	1.75%	0.57%	ESTD-B
ESTD-C2	1	570747	39856				2	2.078	3.92%	-1.70%		
ESTD-C3	1	572244	37693				2	2.11	5.51%	-0.11%		
ESTD-A3	0.125	71511	4222	4152±1.46%	18203±2.11%	39081±3.08%	8	8.465	5.81%	-5.07%	0.65%	ESTD-C
ESTD-B3	0.5	272751	17760				4	4.01	0.26%	-4.72%		

Table 12. Data of signal measurements of HAEE at concentrations of 0.125 ng/ml (ESTD - A), 0.500 ng/ml (ESTD - B) and 1.000 ng/ml (ESTD - C) in **the interval direct measurement sequence** corresponding to the scheme [A (b), B (b), C (b)] × 3. The measurements were carried out in triplets of three technical repetitions with measurement of the blank signal between each fixative within the triplets. The average value of the area and height of the chromatographic peak corresponding to HAEE was calculated for all three technical repetitions in a series of direct interval measurements. The relative response and the differential of the relative response were calculated within a series of direct interval measur ements for each triplet; the additive effect of horizontal mass transfer was calculated in relation to the reference signal within each triplet over the entire series of direct interval signal measurements. The calculation of the expected integral effect was carried out for each triplet within a series of direct interval measurements using the empirical relative response (calculated) in a series of measurement s of the reference signal.

Standard	Concentration	Peak height	Peak area	Average height			Relative response expected	Relative response calculated	Relative response differential	Additive effect	Expected integral additive effect	
				ESTD-A	ESTD-B	ESTD-C						
ESTD-A1	0.125	71409	4520	70800±1.56%	290854±1.1%	575306±1.69%	8	8.04	0.50%	-10.38%	7.62%	ESTD-A
ESTD-B1	0.5	292703	18595				4	4.106	2.66%	-2.32%		
ESTD-C1	1	567253	36776				2	1.958	2.11%	-3.52%		
ESTD-A2	0.125	71467	4460				8	8.066	0.82%	-10.07%	4.59%	ESTD-B
ESTD-B2	0.5	292696	19530	Average area			4	4.237	5.93%	0.95%		
ESTD-C2	1	572574	36212	4454±1.55%	18970±2.6%	36933±2.2%	2	1.905	4.74%	-0.88%		
ESTD-A3	0.125	69525	4382				8	8.529	6.62%	-4.27%	2.54%	ESTD-C
ESTD-B3	0.5	287163	18786				4	4.209	5.22%	0.24%		
ESTD-C3	1	586090	37812				2	2.027	1.34%	-4.28%		

Thus, the calculation of the integral effect of horizontal mass transfer can be expressed as follows:

$$T_m = \frac{\left(\frac{1}{n}\sum_{i=1}^{n}\gamma_i\right)}{RR_{et}} \times \left(\frac{\sum_{i=1}^{n}|F_i|}{n}\right) \text{ (Formula 11)}$$

Where Tm is the integral expected horizontal mass transfer; γ is the harmonic mean of the relative response of the *i* -th fixed channel within a series of measurements for all triplets; RRet is the relative response of the signal *i* -th fixed value in the reference measurement series; Fi is the empirical (calculated) additive effect of the *i* -th fixed value within the measurement series, taken modulo; n is the number of measurements of *the i* -th fixed value in the measurement series.

The results of the horizontal mass transfer study are presented in Table 8-12. The measurements of the reference signal (Table 8) reflect satisfactory signal stability in five technical replicates over the entire concentration range. The standard deviation in pea k height does not exceed 3.57% (for a concentration of 0.500 ng/mL), and in chromatographic peak area does not exceed 3.26% (for a concentration of 0.500 ng/mL; Table 8). The differential of the relative response is within the acceptable deviation limits: the maximum value is observed for a concentration of 0.125 ng/mL and is 10.88%, which is expected at the limiting or close to the limiting low concentration va lues of the linear range of quantitative assessment.

A detailed analysis of the horizontal mass transfer effect showed that the additive signal is practically not registered in the direct sequential analysis. The maximum positive transfer value can be noted only in the case of a concentration of 0.500 ng/ml, but the effect is not systematic, since it manifested itself only in a single (second) triplet of the direct sequence (

Table 9). In other cases, for all concentrations throughout the entire direct sequence, no positive additive effect is observed, and the modulus of the effect values does not exceed the deviation value of the relative response of the reference signal of the corr esponding concentration standards (

Table 9).

The analysis of the reverse sequence (

Table 10) also did not show a systematic additive signal. With the exception of the concentration of 1.000 ng/ml in the very first tr iplet of the sequence, where such an effect was 1.61%. However, in other cases, a positive effect is not observed, and the modulus of the values also does not exceed the differential of the relative response of the reference signal of the corresponding concentrations.

The random sequence is closest to the conditions of a real analytical sequence, in which each analyzed sample, all other thin gs being equal, is characterized by an uncertainty in the content of the substance being analyzed, therefore the data of a random s equence are of particular interest for analysis. In our case, it was shown that with a random sequence, the additive effect is observed only for concentrations of 0.125 ng/ml and 0.500 ng/ml, and such an effect may tend to repeat, i.e., it can be considere d systematic. However, the value limits are not the same in different triplets. Thus, for a concentration of 0.125 ng/ml, the additive signal was 1.28% and 0.23%, while in the first case the sample comes after the standard of 0.500 ng/ml, and in the second case after the standard with a concentration of 1.000 ng/ml (

Table 11). It is natural that in both cases the additive effect is observed if the sample with the concentration of 0.125 ng/ml follows immediately after the sample with a higher concentration. For the standard of 0.500 ng/ml, the increase in the relative respo nse is also observed within 1.25% and 1.75% in the first and second triplets, respectively. However, in this case the standard of 0.500 n g/ml follows sequentially immediately after the blank sample in the first case and immediately after the sample with a low er concentration (0.125 ng/ml,

Table 11) in the second case. Note, however, that such a signal is not strictly systematic, since the additive signal can be observed only in the first two triplets, while in the third triplet the additive signal is negative in all cases, and the modulus of the ad ditive signal is less than the differential of the relative response of the reference signal of the corresponding concentrations.

The direct interval sequence is also characterized by high signal stability and a low value of the additive signal, which can be noted only for the 0.500 ng/ml standard in the second and third triplets with a value of 0.95% and 0.24%, respectively (

Table 12).

The integral additive effect, i.e. the maximum value of the horizontal mass transfer effect that can be expected during the analysis, satisfies all the requirements of the maximum permissible deviations of concentrations at the average, upper and minimum levels of the linear range of quantitative analysis. The most indicative are the values of the integral value of the additive effect in a random continuous sequence (

Table 11), where the values of such an effect lie within the range of 0.57% - 1.17%. As expected, the maximum integral effects are noted in a direct interval sequence, since the blank sample removes the matrix effect of equilibration of the stationary phase, which is reflected in a gradual shift of the chromatographic peak, as well as more significant fluctuations in the signal by area and height in independent measurements (

Table 12). The results of the integral additive effect in the inverse continuous sequence, where its value directly correlates with the concentration of the analyzed standard, are somewhat contrary to expectations: the maximum value of the integral effect was 10.84% for a concentration of 1,000 ng/ml and is at an extremely close level to the differential of the relative response of the reference signal of the ESTD -A standard with the lowest concentration, which was 10.88% (Table 8). At the same time, the minimum value is noted for a concentration of 0.125 ng/ml and was only 0.41%, which is two orders of magnitude lower than the differential of the relative response in the reference measurements.

Based on the analysis results, it can be stated that the horizontal mass transfer effect does take place, but its effect is insignificant and cannot be critical when making decisions. The transfer effect is noted for concentrations of 0.125 ng/ml and 0.500 ng/ml up to 1.28% and 1.75%. The use of an interval analysis scheme with blank alternating samples does not provide an additional effect, and in some cases increases the signal instability due to over-conditioning of the stationary phase, thus, the interval analysis is irrational in terms of time and resource costs. The maximum expected additive effect (integral effect) does not exceed the differential of the relative response of the reference signal in all studied cases regardless of the analysis scheme, but the minimum integral effect can be noted when using a random continuous analysis sequence, which is as close as possible to the conditions of a real list of sample analysis, where each sample is characterized by an equal uncertainty of the concentration of the substance being analyzed.

The diagnostic and prognostic value of the analytical quantitative method largely depends on the accuracy and reproducibility of the measurements. The accuracy of the measurement allows the researcher to assert that the measured, i.e. calculated, concentration value is as close as possible in its value to the actual concentration in the biological sample, while the reproducibility of the results allows us to talk about the high reliability of the results obtained regardless of the time of the analysis, the characteristics of the sample, the technical repetition, etc. Both factors, accuracy and reproducibility, for a number of reasons depend not only on the operator, but also on the selected extraction method, specific steps of analytical isolation, the separation system, signal recording (instrument and technical features of signal stability under given conditions), as well as compliance with the storage and transportation conditions of the biological matrix.

The rather large complexity of the problem of studying the accuracy and reproducibility of measurements requires taking into account the maximum possible factors indicated above, or their preliminary determination in individual experiments. In this work, we developed a typical experiment for determining the accuracy and reproducibility of measurements, since many analytical quantities are linear. The limits of measurements and sensitivity, horizontal mass transfer, stability of the substance under various storage conditions, etc. were determined by individual experiments and designated in special sections. To conduct the study, an averaged sample of human blood plasma (1200 µl (1.2 ml) was prepared by combining equal volumes (300 µl each) of four human blood plasma samples from a test collection of biological samples. HAEE was added to the resulting matrix to a final concentration of 1 ng / ml (the initial concentration of the working solution of HAEE standard is 10 ng / ml in an aqueous solution of 0.5% formic acid). After thorough mixing, the resulting sample was divided into 9 (nine) aliquots of 120 µl each and frozen at a temperature of -20°C. The resulting aliquots were used over the next three days, three aliquots daily (triplet study) to study the stability and reproducibility of the signal within one experiment and between different experiments after preliminary extraction (Figure 25).

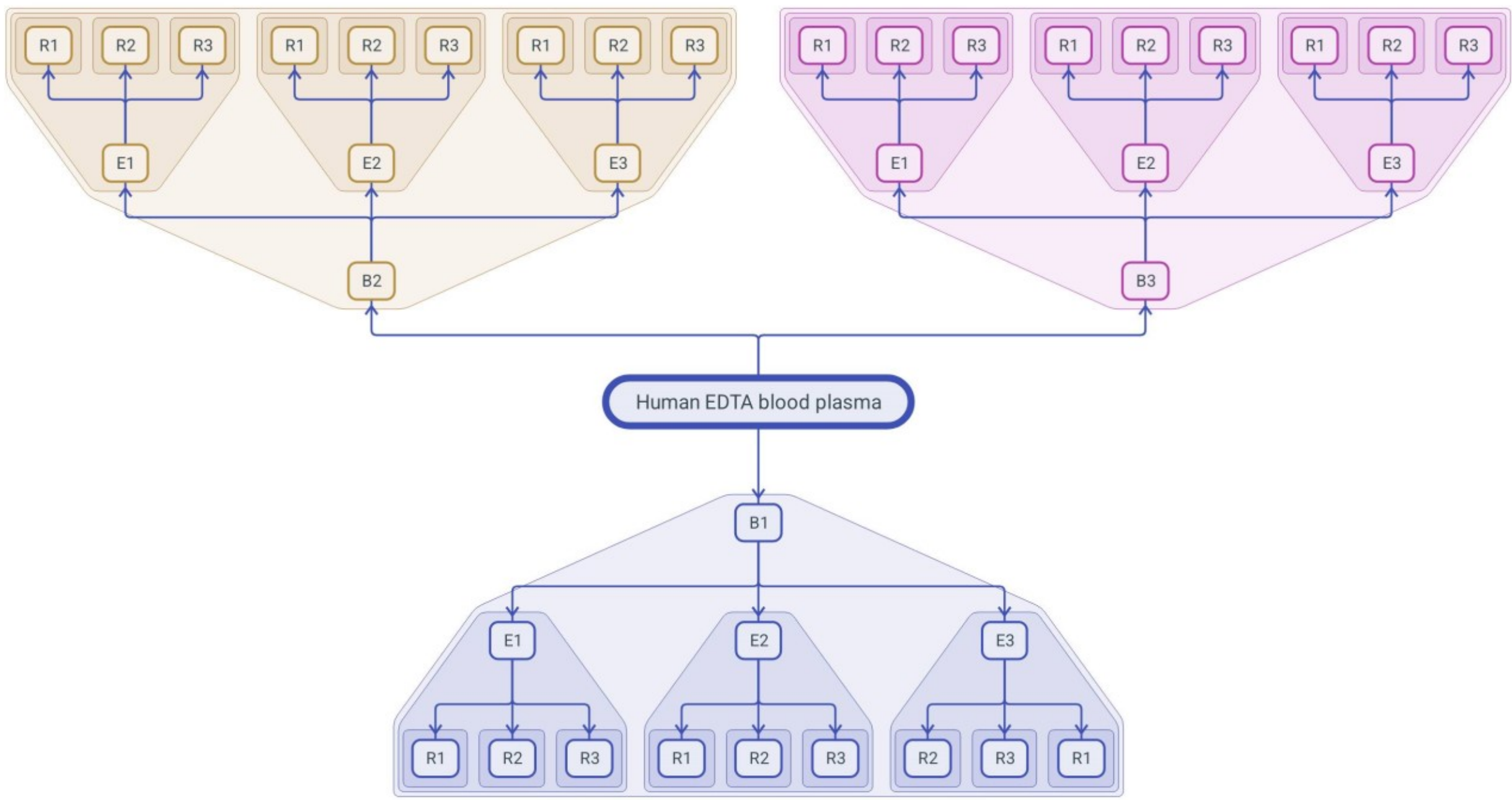

Figure 25. Schematic representation of the experimental design for assessing the stability and reproducibility of the endogenous HAEE signal based on the relative response of the internal standard (labeled HAEE) at a final concentration of 1 ng/mL. The experiment was carried out over three consecutive days (B1, B2, and B3). On each experimental series, endogenous HAEE and the internal standard of HAEE were extracted from three identical biological samples (E1, E2, and E3) for further assessment of the signal reproducibility in the proportions E1:E2, E2:E3, and E3:E1. In each sample, the signal measurements based on the chromatographic peak height were performed in three technical replicates (R1, R2, and R3). The stability of the endogenous HAEE signal was assessed between series B1, B2 and B3 in the proportions B1:B2, B2:B3 and B3:B1, with the following proportional elements being included within each experiment-B: [$E1_j$/ $E2_j$/ $E3_j$]: [$E1_{j+1}$ / $E2_{j+1}$ / $E3_{j+1}$].

The measurements were performed by the area and by the signal height, however, the concentration of the endogenous HAEE in relation to the internal standard of HAEE, as the final result of the analytical measurement method, was chosen as the value for comparing stability and reproducibility. The choice of assessing the measurement results by concentration, i.e. by the relative response, rather than by absolute dimensionless quantities, is more rational, since the reproducibility by absolute value can vary significantly due to the extraction conditions (solid-phase), i.e. the quality of the stationary phase of the mixed anion-exchange carrier, as well as the reproducibility between different samples at the LLE extraction level. However, distortions and errors in the extraction conditions will be reflected equally on the non-labeled and labeled HAEE due to the identity of their physicochemical properties.

Thus, determination of the stability and reproducibility of the signal by the relative response reflects the real stability and reproducibility of the signal between experiments (on the first, second and third days of experimental measurements) and within an experiment (within a triplet of samples). In total, we obtained 27 individual measurements, which correspond to the measurement of each of the nine samples (aliquots) in three technical replicates; each experimental day corresponds to nine individual measurements: three technical replicates for each of the three samples (Table 13).

Table 13. Summary data on determination of signal stability and reproducibility (concentration of non-labeled HAEE by relative response) during three independent experiments (three days of measurements; column with indices B1, B2 and B3) and measurements of three biological samples within one independent experiment (column with indices E1, E2 and E3) by three technical repetitions for each sample (indices R1, R2 and R3). Stability and reproducibility were assessed by the relative response of the signal (chromatographic peak height) of endogenous HAEE to the signal of ISTD (labeled HAEE) added at a known final concentration (1 ng/ml).

Series (day of measurements)	Experiment	Technical replay	Peak height [^{13}C] labeled HAEE	Peak height of non-labeled HAEE	Calculated concentration of non-labeled HAEE pg/ml	Average altitude [^{13}C] labeled HAEE	Average concentration of non-labeled HAEE, pg/ml	Standard deviation of height [^{13}C] labeled HAEE	Standard deviation of concentration of non-labeled HAEE	Intraday reproducibility by concentration	Inter-experimental stability by concentration of non-labeled HAEE
B1	E1	R1	801204	51486	64.26	931853	63.28	13.94%	1.63%	0.982	0.975
		R2	933428	59162	63.38						
		R3	1060928	65993	62.2						
	E2	R1	840659	53944	64.17	837342	64.47	0.34%	0.75%	1.027	
		R2	835622	53655	64.21						
		R3	835744	54349	65.03						
	E3	R1	764067	48622	63.64	732770	62.75	3.73%	1.29%	0.992	
		R2	713726	44284	62.05						
		R3	720518	45087	62.58						
B2	E1	R1	829687	52419	63.18	857052	63.62	4.15%	0.66%	1.017	0.989
		R2	897311	57446	64.02						
		R3	844158	53740	63.66						
	E2	R1	945912	59311	62.7	943488	62.54	0.25%	1.05%	0.986	
		R2	943243	59518	63.1						
		R3	941308	58190	61.82						
	E3	R1	1051478	67034	63.75	1145058	63.4	10.56%	1.54%	0.996	
		R2	1102168	68659	62.29						
		R3	1281527	82204	64.15						
B3	E1	R1	1005819	63785	63.42	1055907	63.58	4.12%	0.75%	1.032	1.037
		R2	1084602	69538	64.11						
		R3	1077299	68083	63.2						
	E2	R1	1055914	65406	61.94	1060169	61.63	0.39%	0.55%	0.97	
		R2	1060343	65405	61.68						
		R3	1064250	65204	61.27						
	E3	R1	1458296	92127	63.17	1454102	63.57	0.45%	0.57%	1	
		R2	1457391	92757	63.65						
		R3	1446620	92422	63.89						

To assess the reproducibility of the signal between individual samples within one experiment (E1, E2 and E3; Table 13), the ratio of the calculated measured concentration of endogenous HAEE by the relative response in the proportions E1:E2, E2:E3 and E3:E1 was used. Accordingly, to assess the stability of the signal of endogenous HAEE by the relative response between experiments (B1, B2 and B3; Table 13), the proportions B1:B2, B2:B3 and B3:B1 were used, with the following elements of the proportions being included within each experiment-B: ([$E1_j$ / $E2_j$ / $E3_j$] : [$E1_{j+1}$ / $E2_{j+1}$ / $E3_{j+1}$]). In an ideal model situation, any ratio of E-indices or B-indices should tend to one, which indicates high stability and reproducibility of the measured concentration of endogenous HAEE in the biological matrix, as well as the reproducibility of the extraction method and signal recording under given conditions.

The results of the study showed that variations within one biological sample in technical replicates and within one experiment can be significant. In particular, the standard deviation of the absolute signal value (chromatographic peak height) can be up to 10% and 13% in the case of samples B 1 E 1 and B 3 E 3, respectively (Table 13). Nevertheless, the average standard deviation for the entire sample of measurements (27 independent measurements) does not exceed 4.21%. Despite the cases of significant variation in the absolute signal value, as indicated above, the standard deviation of the concentration of endogenous HAEE by the relative response to the internal standard of (labeled HAEE) does not exceed 1.63% (in the case of sample B1E1), and the minimum deviation is 0.55% (in the case of sample B3E3); The average value of the standard deviation of the measured concentration of endogenous substance A1 based on the relative response of the HAEE internal standard was 0.98%, i.e. it does not exceed 10 pg/ml of the theoretical concentration of the HAEE internal standard (Table 13). The intraday reproducibility of the signal (absolute concentration of endogenous HAEE) is within 97%–103 (Table 13), which fully satisfies the requirements of the maximum limits of signal variation at the average and minimum concentration limits [m] applicable in the case under study [n] and set at 20%, or ±10%. The interexperimental stability of the signal (measured concentration of endogenous HAEE based on the relative response of the HAEE internal standard) was from 97% to 103%, which also corresponds to the limits of intraday signal stability (Table 13) and fully meets the requirements of the accuracy and reproducibility of measurements of the analytical quantitative method. Thus, the standard deviation of the endogenous substance concentration (63.2 pg/ml), calculated at the level of σ = 1.28% based on the results of all independent measurements, is fully within the permissible detected limits of signal stability and reproducibility, calculated at the level of 97%–103%, or ±3% from the baseline value, which is also significantly less than the permissible limits of signal reproducibility and stability at the level of ±10% from the baseline value.

[m] The standards of analytical research require the level of signal reproducibility at the lower and middle concentration limits of the established linear dependence within 90%–100%, or no more than 20% (or in the range of ±10% of the base value).

[n] In the test sample, the measured concentration is 63.2 pg/mL with a dispersion of 0.65 based on 27 independent measurements (Table 13), which is closer to the lower limits of the concentration range of the linear dependence (limit of quantification 25 pg/mL, detection limit (nominal) 10 ng/mL), which gives the right to use more stringent criteria for signal variation within the framework of the reproducibility study (see previous note).

Low stability in absolute signal value (chromatographic peak height) in individual cases (B1E1, B1E3, B2E3, B3E3) is most likely due to extraction heterogeneity at the level of mechanical properties of the solid-phase mixed anion-exchange carrier. In accordance with the manufacturer's specifications and the certificate of conformity, heterogeneity of the sorbent (solid-phase carrier) in particle size, pore size in particles (diameter) and total pore volume within ±15% of the nominal value [o] is allowed. In this regard, the researcher admits that the observed signal variation in individual cases is due to variations in the mechanical properties of the solid-phase carrier (sorbent), indicated in the note and generally not affecting the reproducibility and stability of the signal measured by the relative response, as shown by the study results. Due to the identity of the behavior of the endogenous HAEE and internal standard (labeled HAEE) at the extraction stage, regardless of the characteristics of the sorbent in each particular case.

[o] According to the in-box inserted certificate in form 150000219 version A dated January 28, 2020, for the sorbent with lot 0091 (cartridge lot 009130175A), the average pore diameter was 82 A (permissible limits 73 - 89 A), the total pore volume was 1.29 cm^3/g (permissible limits 1.18 - 1.44 cm^3/g), the average particle size was 28.5 µm (permissible limits 25 - 35 µm). At the same time, the test extraction rate ranged from 86% for nitrophenol (at a concentration of 5 µg / ml) to 92% for ketoprofen (at a concentration of 2.5 µg / ml) in six technical replicates with a standard deviation of 0.8% and 0.9% (maximum value 1.1% for salicylic acid), respectively.

Section 10. Linearity of Dilution

The study of dilution linearity and parallelism are necessary stages of validation of methods based on ligand binding, i.e. immunochemical methods of analysis, methods of irreversible binding, non-oriented binding, reactive chemical cross-linking, i.e. such methods to which the concepts of affinity, avidity and constant of dissociation and binding are applicable. In this work, none of the stages has a direct or indirect relation to the methods of binding to the ligand. However, at the request of the Customer, the study of parallelism and linearity of dilution was carried out within the framework of the third stage of R&D.

Dilution linearity defines the ability of a matrix of samples spiked with a detectable substance (analyte) above the upper limit of detection to provide reliable quantification after dilution within the ranges of a standard calibration curve. Samples with virtually no deviation in concentration after factorial dilutions are known to exhibit linearity. This confirms a high degree of precision and demonstrates the flexibility of the assay at different dilutions. It also clarifies whether low concentration samples without dilutions show similar concentrations compared to an equivalent sample concentration obtained by a high concentration spike dilution.

There are several key requirements and definitions for a dilution linearity test. According to the 2011 EMA definitions [12], dilution of samples should not affect precision. If applicable, dilution integrity should be demonstrated by spiking the matrix with the analyte at a concentration above the ULOQ and diluting. Precision should be within the established criteria, i.e. within ±15%. Dilution integrity should cover the dilution applied to the test samples. As recommended by the FDA [13], when applying a bioanalytical method to routine drug analysis, the following recommendations should be considered: *any necessary sample dilutions should be used as a matrix (e.g., person-to-person), eliminating the need to include actual quality control samples in the within-study dilution matrix*. Additionally, concentrations in unknown samples should not be extrapolated below the LLOQ or above the ULOQ of the standard curve. Instead, the standard curve should be extended and retested, or samples with higher concentrations should be diluted and reanalyzed [14] .Ministry of Health, Labor and Welfare of Japan (MHLW) and Welfare of Japan) issued the latest revision of the definitions and requirements for analytical testing in 2014, which concluded that dilution linearity is assessed to confirm the following: (a) The method can appropriately analyze samples at concentrations above the ULOQ of the calibration curve without any distorting effect; (b) The measured concentrations are not affected by dilution within the calibration range. Dilution linearity is assessed by analyzing a QC sample above the ULOQ of the calibration curve and serially diluting it at several concentrations.

To study the linearity of dilution, standard A2 and human blood plasma (1.5 ml) were used, obtained by combining ten individual samples (150 µl) from different patients provided by the Customer (patient CIDp520007989, sample 210003214; patient CIDp780038522, sample 780181939; patient CIDp780036033, sample 780181706; patient CIDp780038560 sample 780182609; patient CIDp520007964, sample 610008939; patient CIDp780032531, sample 780182001; patient CIDp700000106, sample 700000473; patient CIDp700000101, sample 700000411; patient CIDp520007977, sample 610009102; and patient CIDp700000104, sample 700000450).

A total of 196 µl of pooled blood plasma was collected and 4 µl of aqueous of labeled HAEE standard solution (250 ng/ml) were added to it to a final concentration of 5 ng/ml, which was taken as the initial working sample (Fd0) with a dilution factor of one (no sample dilution). Then, serial dilutions were performed with equal volumes of pooled blood plasma according to the 1: 2 scheme, obtaining a series of labeled HAEE standard concentrations from 2.5 ng/ml to 0.156 ng/ml (Table 14), observing the requirements of not going beyond the upper and lower detection limits of the method in order to avoid extrapolation of the results, as well as obtaining a series of at least five dilutions within the calibration curve. The upper dilution with a concentration of 2.5 ng/ml meets the requirement for adding analyte to the matrix at a concentration above the ULOQ, since typical analyte concentrations are measured in the range up to 1 ng/ml. Extraction of each obtained dilution was performed and the concentration of labeled HAEE standard was measured in the final extract in three technical replicates, calculating the area of the empirical concentration from the average sum of the area and the height of the chromatographic peak according to the calibration curve. The result obtained was assessed by the relative error of the empirical concentration to the expected concentration and by the average deviation in the dilution series. Both values should not exceed the threshold of 20% (in accordance with the recommendations of the FDA and EMA), however, in the framework of this work, a user limit of 10% was set due to the high accuracy of the measurement base compared to spectrophotometric and luminescent measurement methods.

Table 14. Data on empirical concentrations (in ng/ml) and relative error (in percent) of measurement to the expected concentration (in ng/ml) according to the calibration curve in five dilutions according to the 1:2 scheme.

Dilution ID	Dilution factor	Expected concentration, ng/ml	Peak area, cnts × s	Peak height, cnts	Empirical concentration, ng/ml	Relative error, %	Average deviation, %
Fd1	2	2.5	12254	192068	2.608	4.33%	2.87%
			12921	201336			
			12663	195525			
Fd2	4	1.25	6761	102227	1.283	2.64%	
			6665	102107			
			6658	102529			
Fd3	8	0.625	3541	52731	0.598	-4.24%	
			3488	52836			
			3420	51779			
Fd4	16	0.313	1585	24987	0.309	-1.20%	
			1650	25139			
			1640	24701			
Fd5	32	0.156	802	12217	0.161	2.80%	
			834	12697			
			811	12331			

The results of the study showed that the empirical concentration in the prepared dilutions corresponds to the expected concentration according to the calibration curve within 2.87% over the entire dilution range. The maximum deviation is observed for dilutions of Fd1 and

Fd3 with factors of 2 and 8, corresponding to expected concentrations of 2.500 ng/ml and 0.625 ng/ml, the minimum for dilution of Fd4 with a factor of 16, corresponding to the expected concentration of 0.156 ng/ml.

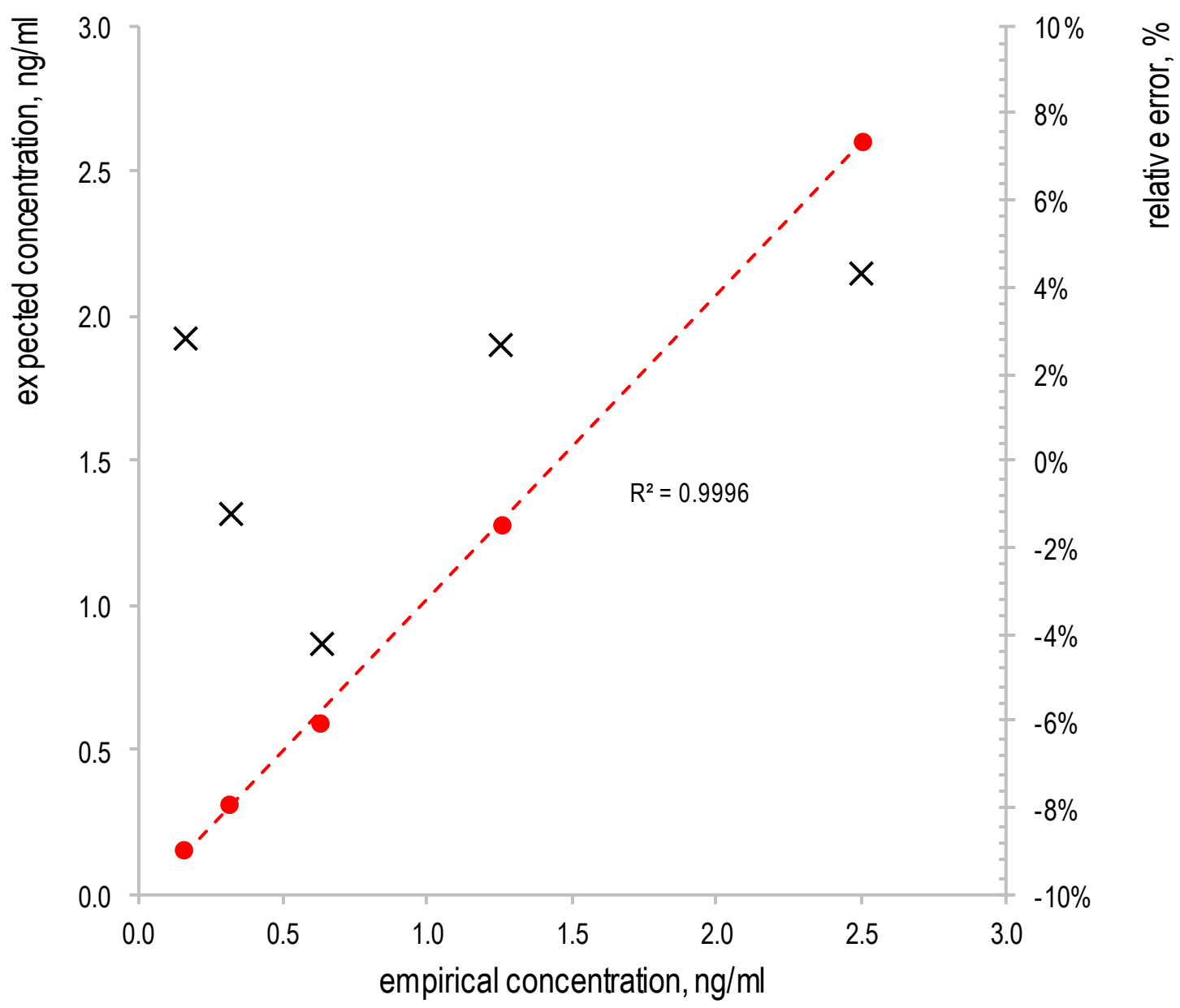

Figure 26. Plot of the distribution of the relative measurement error (**black asterisk**) of linearity between the empirical concentration (red dots) of labeled HAEE standard and its expected concentration in the corresponding dilutions.

The determination coefficient in the series of obtained dilutions was 0.9996 (Figure 26), which reflects more than satisfactory linearity of dilution according to the 1:2 scheme within the dynamic order of concentrations from 2.5 ng/ml to 0.156 ng/ml. The relative measurement error (in relation to the expected concentration of the calibration curve) fluctuates between -4.24% and 4.33%, which is within the limits of measurement accuracy established earlier. Thus, based on the results of the study, it can be stated that satisfactory linearity of dilution in the biological matrix has been established within the dynamic order of concentration, and that there is no distorting effect of the biological matrix on the quality of the signal and the linearity of the concentration dependence on the relative signal response.

Section 11. Parallelism

Parallelism determines whether test samples containing high concentrations of the endogenous analyte provide the same degree of detection on the standard curve after dilutions. Parallelism should generally be performed during screening of test samples. The key difference between parallelism and dilution linearity is that parallelism uses samples containing high levels of the endogeno us analyte and high concentrations of the spiked standard analyte. It is important to use high concentration samples because true parallelism may not be detectable when using sample concentrations close to the lower detection limit. As with dilution linearity, there is no singl e way to perform or interpret parallelism tests.

A common method for assessing parallelism is to back-calculate the concentration of diluted samples. Stevenson and Purushotama [15,16] also proposed plotting dilution-corrected concentration against sample dilution for multiple individuals to establish a weighted method bias where most samples are measured within the assay range and multiple dilutions outside the MRD yield accurate resul ts.

The European Bioanalysis Forum has summarized alternative methods for handling parallelism data. These approaches include plo tting measured concentration versus dilution factor, plotting dilution-corrected concentration versus dilution factor, plotting dilution-corrected relative error versus dilution factor, and plotting concentration versus the inverse logarithm of dilution [17,18]. All of these approaches rely on back-calculation of concentration and allow parallelism to be assessed against predefined ac ceptance criteria.

The EMA rules [19] for validation work for immunoassay systems regulate the following situation: If test samples are available, the parallelism between the calibration standard curve and serially diluted test samples should be assessed to detect possible matrix effects or different affinities for metabolites. A test sample with a high concentration (preferably close to C_{max}) should be diluted to at least three concentrations with a blank matrix. The precision between samples in a dilution series should not exceed 30%.

At the same time, the directory of the Ministry of Health, Labor and Welfare of Japan (MHLW) and 2014 Guidelines for Welfare of Japan state that *parallelism is defined as an established relationship between the dose-response curve of a dilution series of a test sample and that of a series of calibration standards, with no difference between the back-calculated concentrations for several dilutions of the test sample. At the time of issuance of this guideline, domestic and international knowledge has not accumulated and discussions h ave not yet been conducted on the cases in which parallelism has not been established, the reasons for failure to establish parallelism, and the extent to which such failure may affect pharmaceutical development. Therefore, assessment of parallelism is not necessarily required for all analytical methods. However, if parallelism is an inherent problem in an LBA-based bioanalytical method and may cause problems related to the nature of the analyte or method or the data accumulated during pharmaceutical development, scientifically based assess ment and evaluation of the impact on the measured concentrations should be considered to the extent possible.*

In this work, we used five biological samples (human blood plasma provided by the Customer) to investigate parallelism: patient CIDp780038522, sample 780181939; patient CIDp780036033, sample 780181706; patient CIDp780038560, sample 780182609; patient CIDp700000101, sample 700000411; and patient CIDp520007977, sample 610009102.

Standard of labeled HAEE was added to 50 µl of each patient's blank sample to a final concentration of 12 ng/ml and a series of dilutions was prepared according to the scheme $1:3\times2^n$, where n is the dilution number, thus maintaining a multiplicity of three, but maintaining a multiplicity of two between dilution factors (Table 15). Each sample of the resulting dilution was then prepared for each patient and the empirical concentration of the added standard of labeled HAEE was measured in three technical replicates.

Table 15. Dilution scheme with expected concentrations (ng/ml) and results of measuring empirical concentration in each dilution (ng/ml) for each patient (P1 – P5).

Dilution ID	Dilution factor	Expected concentration, ng/ml	Empirical concentration, ng/ml				
			P1	P2	P3	P4	P5
Fd1	1:03	4	4.139	4.172	4.107	4.14	3.939
Fd2	1:06	2	2.04	2.074	2.063	2.061	2.069
Fd3	1:12	1	0.984	0.967	0.93	1.086	0.978
Fd4	1:24	0.5	0.528	0.526	0.527	0.515	0.527
Fd5	1:48	0.25	0.24	0.245	0.235	0.247	0.218

The instrumental measurement of the empirical concentration in each sample for each patient was carried out in three technica l repetitions. The measured concentrations should differ between dilutions by a factor of two. To determine parallelism, the imagin ary concentration, or the empirical concentration adjusted for the dilution factor, was calculated in relation to the Fd1 dilution. That is, the final imaginary concentration in each measurement (for dilutions Fd2 - Fd5) should be as close as possible to the concentration in the Fd1 dilution. In this way, parallelism between patients and between dilutions in a series of measurements is determined.

Table 16. Results of calculating the empirical concentration (ng/ml) adjusted for the dilution factor (relative to Fd1) for each patient (P1–P5), the average concentration adjusted for the dilution factor for the sample of patients within each dilution, and the relative error in the average empirical concentration.

Breeding	Dilution factor	Empirical concentration corrected for dilution factor (relative to Fd1), ng/ml					Mean concentration corrected for dilution, ng/ml	Relative error, %
		P1	P2	P3	P4	P5		
Fd1	3	4.139	4.172	4.107	4.14	3.939	4.099	0
Fd2	6	4.08	4.147	4.127	4.122	4.138	4.123	0.57
Fd3	12	3.935	3.868	3.72	4.342	3.912	3.955	-3.51
Fd4	24	4.224	4.204	4.213	4.117	4.216	4.195	2.32
Fd5	48	3.835	3.924	3.752	3.951	3.488	3.79	-7.55

Based on the results of the measurements, it was established that within one dilution and between dilutions, both for each individual patient and between patients, the integrity (integrity) of the concentration is maintained with a correction for the dilution factor (imaginary concentration) throughout the entire dynamic range of concentrations (one order of concentration from 0.25 ng/ml to 4.00 ng/ml; Table 15).

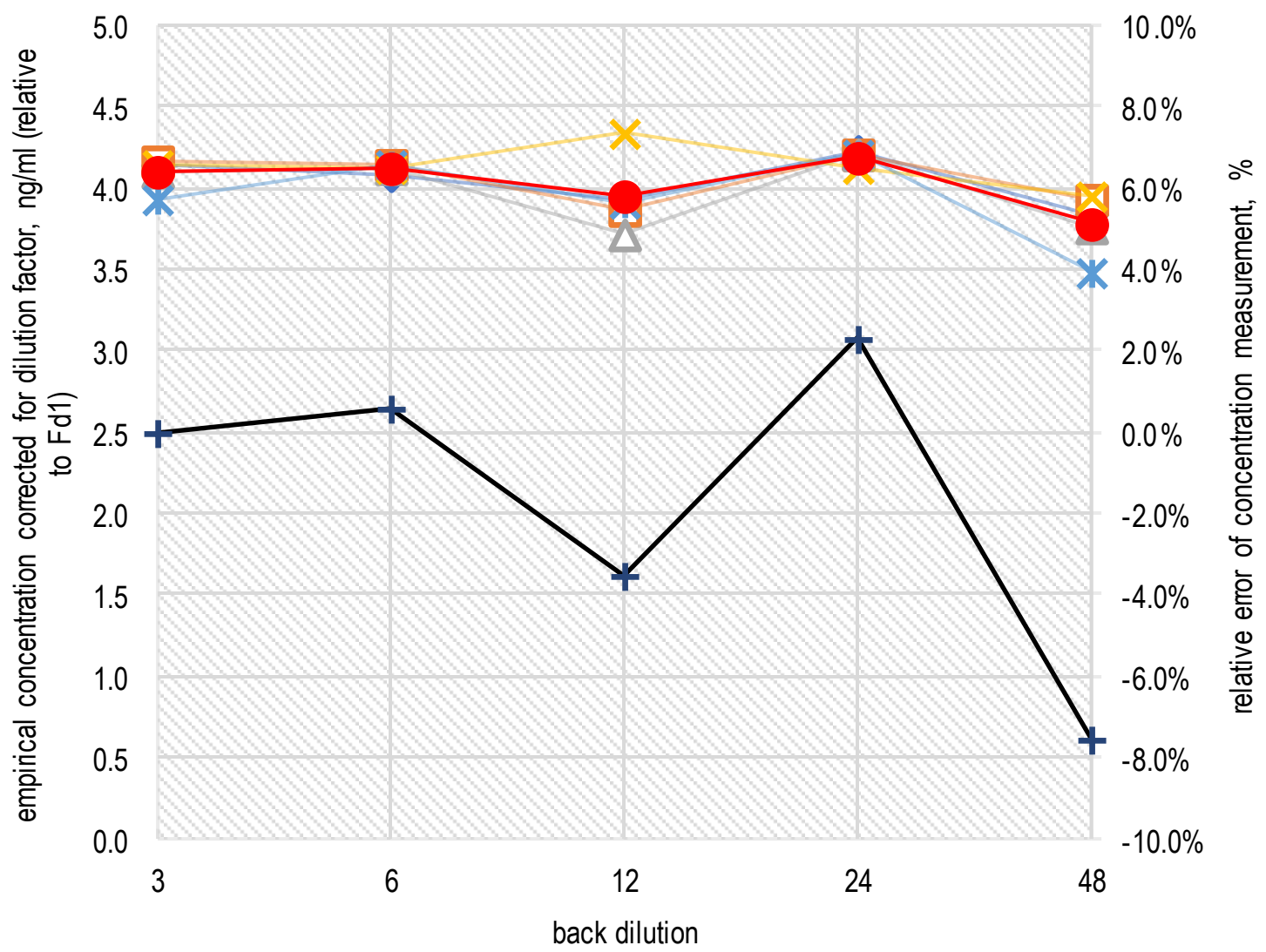

Figure 27. Plot of the dependence of the reciprocal dilution on the concentration, corrected for the dilution factor (red line). The trend of the mean concentration adjusted for the dilution factor for all five patients (biological samples) is shown; the of variation of the relative error (in percent) of the measurement of the empirical concentration adjusted for the dilution factor is shown **in black**.

The relative measurement error between dilutions in relation to the Fd1 dilution does not exceed the established standards, i.e. the accuracy between samples in a dilution series should not exceed 30%. The linear range of the relative error between samples is from 0.57% to -7.55% (Table 16). Strict parallelism of measurements is observed between the studied samples in a dilution series from 1: 3 to 1: 48 (Figure 27) with an average relative measurement error of concentration adjusted for the dilution factor not exceeding 2.79%.

Section 12. Cross-platform Validation. Measurement of Endogenous HAEE In Human Blood Plasma Samples Using UPLC-QQQ-SRM.

The reproducibility of the results of measuring the concentration of HAEE peptide in human blood plasma on a triple quadrupole (UPLC-QQQ-SRM) was carried out on a sample set received in 2024. Due to the fact that the measurement of samples from 2024 on the UPLC-QTOF-MS/MS was carried out randomly without reference to the study group or source (clinical center), additional randomization of samples for conducting studies on the UPLC-QQQ-SRM platform was not carried out, however, for sample preparation and instrumental measurement, alternative aliquots that were not previously used in the work were taken, which were stored at a temperature of -20 ° C. Thus, for the validation study, the first one hundred (100) samples were randomly selected from the study list on the UPLC-QTOF-MS/MS without attention the samples to the study group.

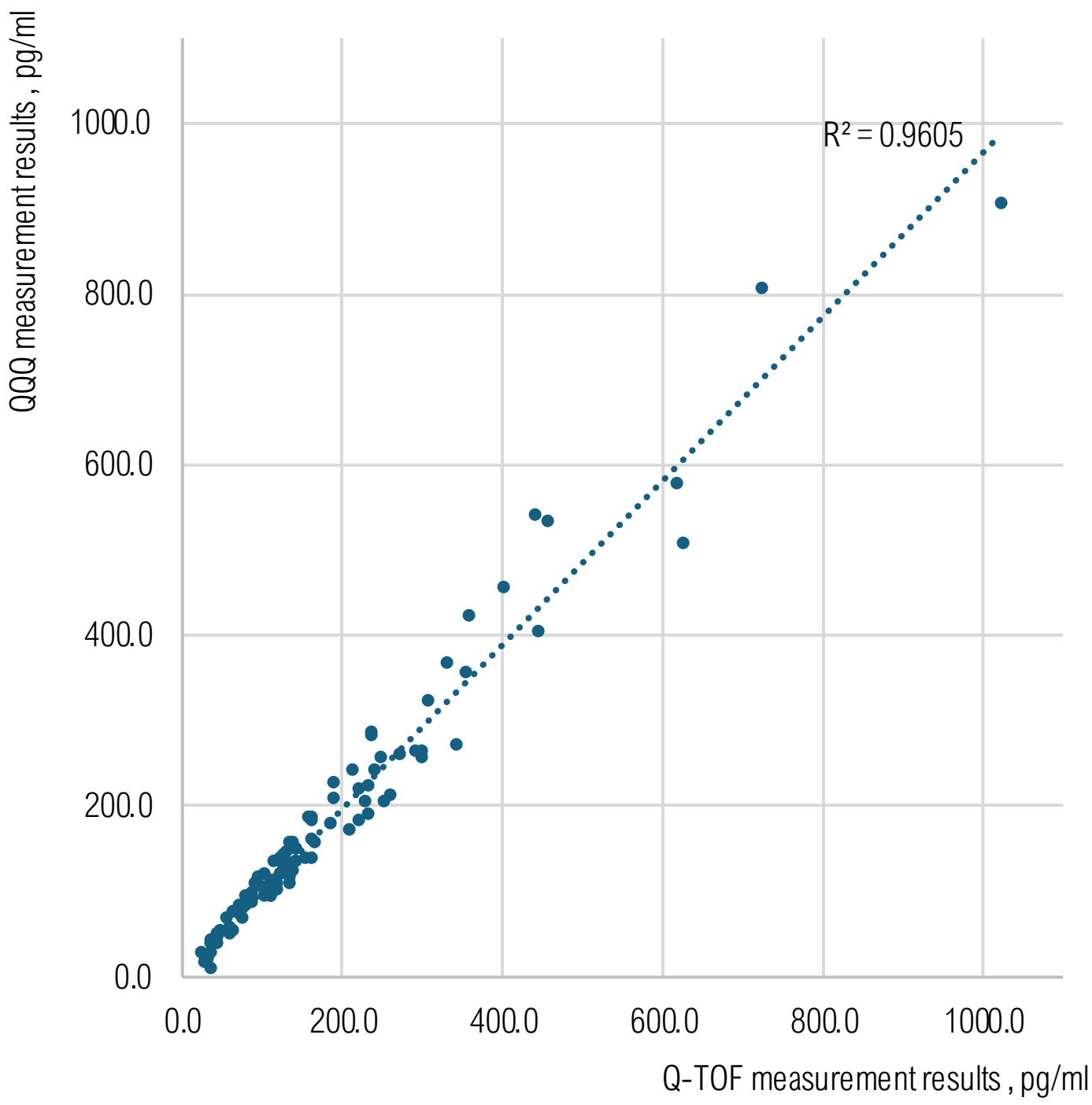

Figure 28. Distribution diagram of inter-platform measurements between Q - TOF and QQQ. The higher the convergence of the final measurements of endogenous HAEE substance concentration, the closer the Pearson coefficient tends to unity.

Based on the results of the measurements, a graph of the distribution of the final values of the concentration of endogenous HAEE in the blood plasma between the two platforms studied was constructed (Figure 28).

Kruskal -Wallis test show that there are no statistically significant differences in the measurement results between the two selected platforms (p = 0.91826, H = 0.0105).

Biserial correlation test show almost identical results for the coefficient of determination for both platforms (r = -0.67408, $p < 10^{-4}$ for Q - TOF; and r = -0.68719, $p < 10^{-4}$ for QQQ).

The results of the t-test for two independent measurements also show no statistically significant differences between the results of two measurements by two different platforms: t = 0.08109, p = 0.467225. The average error of inter-platform measurements was 26.71%, which more than satisfies the requirements of the method transformation, taking into account the use of different aliquots, different storage periods of aliquots, and different physical approaches to signal recording (detector types).

According to the results of descriptive statistics, the minimum concentration value on QTOF was 23.5 pg/ml, on QQQ 8.6 pg/ml; the maximum value of the measured concentration on QTOF was 1022.5 pg/ml, on QQQ – 904.8 pg/ml. The standard measurement error of QTOF was 15.82 pg/ml, the standard measurement error on QQQ was 15.51 pg/ml, the standard deviation on QTOF was 158.17 pg/ml, the standard deviation of measurement on QQQ was 155.03 pg/ml; the asymmetry coefficient of distribution of concentration values on QTOF was 2.581, on QQQ the asymmetry coefficient was 2.334.

Table 17. Measurement of HAEE in one hundred samples of EDTA human blood plasma from NBS using an internal standard with two stable carbon isotopes [13C] by ultra-high-pressure high-performance liquid chromatography with a triple quadrupole detector in the selective multiple ion formation reaction monitoring mode (UPLC - QQQ - SRM) and comparison with the results of endogenous HAEE measurement in EDTA human blood plasma samples of different aliquot identifiers, but identical clinical cases by high-pressure high-performance liquid chromatography with a quadrupole time-of-flight detector in the tandem scanning mode with selective isolation of parent ions (HPLC - QTOF - MS/MS). Research Groups: G01A – early Alzheimer's disease (AD) G01B – mild AD; G01C – moderate AD; G01D – severe AD; G02 – control group (healthy volunteers with no signs od AD or any neurogenerative disease).

Characteristics and identifiers of measurement samples			Q-TOF measurement results										QQQ measurement results											Cross-platform reproducibility		
Sample serial number	Research group	Patient ID	Sample ID	A-[12C], cnts *s	H-[12C], cnts (q/s)	A-[13C], cnts *s	H-[13C], cnts (q/s)	[12C] H/A	[13C] H/A	C[12C13C]-A	C[12C/13C]-H	Concentration, pg/ml	Intersection of results A and H	Sample ID	A-[12C], cnts *min	H-[12C], cnts (q/min)	A-[13C], cnts *min	H-[13C], cnts (q/min)	[12C] H/A'	[13C] H/A'	C[12C13C]-A'	C[12C/13C]-H'	Concentration, pg/ml	Intersection of results A and H	Relative, %	Absolute, pg/ml
1	G01A	CIDp530001416	780239056	55	590	528	5771	10.73	10.93	0.104	0.102	103.3	101.86%	780239055	856	9845	7251	84052	11.5	11.59	0.118	0.117	117.7	100.84%	13.94%	14.4
2	G01B	CIDp700000076	700001948	61	559	409	4492	9.17	10.98	0.149	0.125	124.6	119.72%	700001951	945	10021	7640	83799	10.6	10.97	0.124	0.12	121.7	103.48%	-2.33%	-2.9
3	G01A	CIDp530001419	780239067	32	414	601	9865	12.92	16.42	0.053	0.042	42	127.09%	780239064	378	4530	7596	97039	11.97	12.78	0.05	0.047	48.3	106.73%	15.00%	6.3
4	G01A	CIDp530001424	780239088	68	656	453	5137	9.65	11.34	0.15	0.128	127.8	117.43%	780239085	918	10389	7190	89390	11.31	12.43	0.128	0.116	122	109.92%	-4.54%	-5.8
5	G02	CIDp780040067	780227175	113	1192	453	4768	10.55	10.52	0.249	0.25	249.7	99.72%	780227176	1785	22490	7068	88346	12.6	12.5	0.253	0.255	253.6	99.19%	1.56%	3.9
6	G02	CIDp780040083	780238112	272	3000	592	9812	11.03	16.58	0.46	0.306	305.8	150.35 %	780238111	2682	32434	8325	100312	12.09	12.05	0.322	0.323	322.8	99.65%	5.56%	17
7	G01A	CIDp530001417	780239130	21	255	535	5665	12.12	10.6	0.039	0.045	42.2	87.40%	780239126	352	4296	7455	92045	12.22	12.35	0.047	0.047	47	101.07%	11.37%	4.8
8	G02	CIDp780040077	780227297	139	1576	603	6344	11.34	10.52	0.231	0.248	239.6	92.80%	780227296	2166	21490	8765	90867	9.92	10.37	0.247	0.237	241.9	104.49%	0.96%	2.3
9	G01A	CIDp700000065	700000287	63	701	508	4650	11.12	9.15	0.124	0.151	124	82.28%	700000289	1100	12410	8143	88530	11.29	10.87	0.135	0.14	137.7	96.33%	11.05%	13.7
10	G01A	CIDp780040590	780227478	52	583	477	4971	11.21	10.41	0.109	0.117	113.1	92.90%	780227473	948	12266	7030	95743	12.93	13.62	0.135	0.128	131.6	105.29%	16.36%	18.5
11	G01A	CIDp780040599	780227460	60	651	623	10376	10.84	16.67	0.096	0.063	62.7	153.72%	780227456	442	4993	8345	96478	11.29	11.56	0.053	0.052	52.4	102.42%	-16.43%	-10.3
12	G01A	CIDp780040584	780227411	21	219	616	6571	10.44	10.67	0.034	0.033	33.8	102.15%	780227406	198	2313	6797	86195	11.69	12.68	0.029	0.027	28	108.50%	-17.16%	-5.8
13	G02	CIDp780040084	780238132	121	1177	442	5154	9.73	11.65	0.274	0.228	228.5	119.77%	780238130	1828	20335	8713	103699	11.12	11.9	0.21	0.196	203	107.00%	-11.16%	-25.5
14	G02	CIDp780040076	780227292	119	1167	521	5483	9.81	10.52	0.228	0.213	220.6	107.21%	780227288	1987	21263	9011	97401	10.7	10.81	0.221	0.218	219.5	101.02%	-0.50%	-1.1
15	G02	CIDp780040061	780227245	141	1487	535	5816	10.54	10.88	0.264	0.256	259.7	103.19%	780227243	1611	20425	7561	98911	12.68	13.08	0.213	0.206	209.8	103.20%	-19.21%	-49.9
16	G02	CIDp780040068	780227231	137	1188	517	5373	8.67	10.39	0.265	0.221	221.1	119.87%	780227234	1461	15541	7992	87051	10.64	10.89	0.183	0.179	180.7	102.41%	-18.27%	-40.4
17	G02	CIDp780040088	780227378	152	1707	585	5942	11.23	10.15	0.26	0.287	273.6	90.39%	780227376	1993	26344	7472	103924	13.22	13.91	0.267	0.253	260.1	105.21%	-4.93%	-13.5
18	G02	CIDp780040085	780233999	120	1369	631	7213	11.41	11.42	0.19	0.19	190	100.15%	780238187	1901	22191	8452	99024	11.67	11.72	0.225	0.224	224.6	100.38%	18.21%	34.6
19	G02	CIDp530001429	530003247	80	958	341	3972	11.98	11.65	0.235	0.241	238	97.25%	530003245	2586	28668	8794	107181	11.08	12.19	0.294	0.267	280.8	109.96%	17.98%	42.8
20	G02	CIDp530001428	530003452	105	929	418	4240	8.84	10.14	0.251	0.219	235.2	114.68%	530003448	2302	26817	8010	95874	11.65	11.97	0.287	0.28	283.6	102.74%	20.58%	48.4
21	G01A	CIDp780040598	780239165	40	437	539	6522	11.04	12.1	0.073	0.067	70.3	109.62%	780239166	710	8046	8937	95192	11.33	10.65	0.079	0.085	82	94.02%	16.64%	11.7
22	G02	CIDp780040086	780227308	184	1821	629	7253	9.89	11.53	0.292	0.251	251.1	116.51%	780227305	1772	20483	8776	99680	11.56	11.36	0.202	0.205	203.8	98.27%	-18.84%	-47.3
23	G01A	CIDp700000061	700001873	16	157	559	6721	9.84	12.03	0.029	0.023	23.5	122.20%	700001870	194	2091	7685	82760	10.8	10.77	0.025	0.025	25.3	99.68%	7.66%	1.8
24	G02	CIDp780040046	780227316	67	618	416	4008	9.23	9.64	0.161	0.154	157.7	104.49%	780227317	1508	17567	8374	93331	11.65	11.14	0.18	0.188	184.2	95.65%	16.80%	26.5
25	G02	CIDp780040087	780227371	95	938	486	5828	9.87	11.99	0.195	0.161	160.9	121.46%	780227367	1446	17250	7848	93999	11.93	11.98	0.184	0.184	183.9	100.37%	14.29%	23
26	G01A	CIDp780040589	780227402	38	350	466	5925	9.22	12.7	0.081	0.059	59.2	137.82%	780227397	442	5209	8192	98699	11.79	12.05	0.054	0.053	53.4	102.22%	-9.80%	-5.8
27	G02	CIDp780040079	780238159	110	1145	517	5491	10.41	10.62	0.213	0.209	210.7	102.03%	780238155	1319	16385	7789	97805	12.42	12.56	0.169	0.168	168.5	101.09%	-20.03%	-42.2
28	G02	CIDp780040075	780238123	90	907	557	5686	10.07	10.22	0.162	0.159	160.6	101.40%	780238120	1310	15157	7185	84573	11.57	11.77	0.182	0.179	180.8	101.73%	12.58%	20.2
29	G02	CIDp780040071	780227342	88	979	550	5950	11.12	10.82	0.16	0.165	162.3	97.26%	780227339	655	13561	4904	97157	20.7	19.81	0.134	0.14	136.6	95.70%	-15.83%	-25.7
30	G02	CIDp780040060	780227224	179	1673	579	6145	9.35	10.62	0.309	0.272	290.9	113.60%	780227221	2062	25010	7592	97691	12.13	12.87	0.272	0.256	263.8	106.07%	-9.32%	-27.1
31	G01C	CIDp780040588	780227451	88	1038	596	6449	11.79	10.82	0.148	0.161	154.3	91.74%	780227446	983	11934	7346	86501	12.14	11.78	0.134	0.138	136	97.03%	-11.86%	-18.3
32	G02	CIDp780040070	780227331	245	2396	469	5381	9.78	11.48	0.523	0.445	445.3	117.42%	780227329	3395	39814	8157	103249	11.73	12.66	0.416	0.386	401	107.94%	-9.95%	-44.3
33	G02	CIDp780040082	780238175	322	3434	504	5625	10.66	11.16	0.639	0.61	624.9	104.70%	780238177	4071	46281	7910	93100	11.37	11.77	0.515	0.497	505.9	103.53%	-19.04%	-119
34	G02	CIDp780040078	780227360	238	2520	642	7521	10.59	11.71	0.37	0.335	352.8	110.59%	780227356	3104	35260	8484	102691	11.36	12.1	0.366	0.343	354.6	106.54%	0.51%	1.8
35	G01A	CIDp700000075	700001938	118	1220	572	8707	10.34	15.22	0.206	0.14	140.2	147.22%	700001936	1219	13510	8690	88561	11.09	10.19	0.14	0.153	146.4	91.93%	4.42%	6.2
36	G01A	CIDp530001420	780239074	78	880	609	6027	11.28	9.89	0.128	0.146	137.1	87.65%	780239077	1248	13723	8236	85388	11	10.37	0.152	0.161	156.2	94.27%	13.93%	19.1
37	G02	CIDp530001433	530003439	408	3908	526	5787	9.58	11.01	0.776	0.675	725.6	114.92%	530003438	7003	78261	9008	94403	11.17	10.48	0.777	0.829	803.3	93.78%	10.71%	77.7
38	G02	CIDp780040080	780238165	294	3684	607	10238	12.53	16.86	0.484	0.36	359.9	134.57%	780238164	3535	39135	8017	98173	11.07	12.25	0.441	0.399	419.8	110.60%	16.64%	59.9
39	G02	CIDp780040073	780238148	163	1703	482	4967	10.45	10.31	0.338	0.343	340.6	98.68%	780238147	2244	25492	8098	96621	11.36	11.93	0.277	0.264	270.5	105.03%	-20.58%	-70.1
40	G02	CIDp780040064	780227212	184	1748	535	5552	9.5	10.38	0.344	0.315	329.6	109.28%	780227215	3472	33563	9293	93532	9.67	10.07	0.374	0.359	366.3	104.13%	11.13%	36.7
41	G02	CIDp780040081	780238140	279	3083	466	6989	11.05	14.99	0.598	0.441	441.2	135.60%	780238137	4988	52077	9247	96837	10.44	10.47	0.539	0.538	538.6	100.30%	22.08%	97.4

Characteristics and identifiers of measurement samples				Q-TOF measurement results									QQQ measurement results											Cross-platform reproducibility		
Sample serial number	Research group	Patient ID	Sample ID	A-[12C], cnts *s	H-[12C], cnts (q/s)	A-[13C], cnts *s	H-[13C], cnts (q/s)	[12C] H/A	[13C] H/A	C[12C13C]-A	C[12C/13C]-H	Concentration, pg/ml	Intersection of results A and H	Sample ID	A-[12C], cnts *min	H-[12C], cnts (q/min)	A-[13C], cnts *min	H-[13C], cnts (q/min)	[12C] H/A'	[13C] H/A'	C[12C13C]-A'	C[12C/13C]-H'	Concentration, pg/ml	Intersection of results A and H	Relative, %	Absolute, pg/ml
42	G01C	CIDp700000129	700001774	51	562	403	5012	11.02	12.45	0.127	0.112	119.5	112.92%	700001773	907	9355	9228	93633	10.32	10.15	0.098	0.1	99.1	98.32%	-17.07%	-20.4
43	G01C	CIDp530001415	780239116	64	636	440	4691	9.93	10.66	0.145	0.136	140.6	107.31%	780239114	1257	12851	9671	93348	10.22	9.65	0.13	0.138	133.9	94.40%	-4.77%	-6.7
44	G02	CIDp780040074	780238196	131	1354	442	4439	10.34	10.04	0.296	0.305	300.7	97.12%	780238193	2377	25769	9127	97810	10.84	10.72	0.26	0.263	262	98.85%	-12.87%	-38.7
45	G01C	CIDp700000123	700001713	97	825	563	5767	8.5	10.24	0.172	0.143	143.1	120.44%	700001715	1498	14050	10292	94164	9.38	9.15	0.146	0.149	147.4	97.56%	3.00%	4.3
46	G01B	CIDp700000071	700002028	67	702	532	5328	10.48	10.01	0.126	0.132	128.9	95.51%	700002027	1060	13773	7989	101423	12.99	12.69	0.133	0.136	134.3	97.71%	4.19%	5.4
47	G01B	CIDp700000128	700001764	55	593	422	4321	10.78	10.23	0.13	0.137	133.8	94.86%	700001762	1399	15733	9380	98882	11.25	10.54	0.149	0.159	154.2	93.72%	15.25%	20.4
48	G01C	CIDp530001423	780239101	64	758	539	5921	11.84	10.99	0.119	0.128	123.4	92.76%	780239097	1125	11075	9186	94122	9.85	10.25	0.122	0.118	120.1	104.06%	-2.67%	-3.3
49	G01A	CIDp780040600	780227466	36	440	590	10007	12.22	16.97	0.061	0.044	44	138.84%	780227464	431	4338	9720	93292	10.06	9.6	0.044	0.047	45.5	95.44%	3.41%	1.5
50	G01A	CIDp700000072	700001906	59	584	561	6352	9.9	11.32	0.105	0.092	98.6	114.33%	700001909	928	9881	8659	95853	10.65	11.07	0.107	0.103	105.2	103.95%	6.69%	6.6
51	G01C	CIDp780040585	780227420	87	993	532	9130	11.42	17.15	0.163	0.109	108.9	150.17%	780227418	841	9477	8823	102398	11.27	11.61	0.095	0.093	94	102.97%	-13.68%	-14.9
52	G01A	CIDp700000122	700001844	62	890	546	11538	14.36	21.15	0.114	0.077	77.2	147.25%	700001846	902	8920	9791	98494	9.89	10.06	0.092	0.091	91.4	101.68%	18.39%	14.2
53	G01C	CIDp700000124	700001859	58	626	651	7286	10.78	11.19	0.089	0.086	87.5	103.74%	700001858	787	8590	8910	105159	10.92	11.8	0.088	0.082	85	108.09%	-2.86%	-2.5
54	G01C	CIDp780040583	780227390	60	593	519	6262	9.87	12.06	0.116	0.095	94.8	122.22%	780227387	867	10741	7720	94317	12.39	12.22	0.112	0.114	113.1	98.61%	19.30%	18.3
55	G01A	CIDp700000073	700001919	67	584	506	5215	8.72	10.31	0.132	0.112	112.1	118.17%	700001920	866	9046	9184	100673	10.44	10.96	0.094	0.09	92.2	104.99%	-17.75%	-19.9
56	G02	CIDp780040065	780227185	123	1391	535	6023	11.31	11.27	0.23	0.231	230.6	99.63%	780227187	1558	18680	7061	85217	11.99	12.07	0.221	0.219	220	100.69%	-4.60%	-10.6
57	G01A	CIDp700000120	700001834	68	678	488	5247	9.98	10.74	0.139	0.129	134.3	107.67%	700001830	1059	10775	9343	94589	10.18	10.12	0.113	0.114	113.7	99.47%	-15.34%	-20.6
58	G01A	CIDp700000074	700001930	58	692	539	8878	11.93	16.47	0.108	0.078	78	138.10%	700001931	715	6700	8881	80856	9.37	9.1	0.081	0.083	81.8	97.21%	4.87%	3.8
59	G01A	CIDp700000078	700002047	53	546	488	4768	10.3	9.76	0.109	0.115	111.6	94.76%	700002039	1051	10493	10207	93555	9.98	9.17	0.103	0.112	107.6	91.84%	-3.58%	-4
60	G01A	CIDp700000127	700001749	58	552	502	5393	9.52	10.75	0.116	0.102	109	112.99%	700001751	849	9484	8645	88976	11.18	10.29	0.098	0.107	102.4	92.08%	-6.06%	-6.6
61	G01B	CIDp700000062	700001991	51	505	422	4963	9.9	11.75	0.121	0.102	101.8	118.69%	700001989	922	9484	9879	99669	10.28	10.09	0.093	0.095	94.3	98.11%	-7.37%	-7.5
62	G02	CIDp780040066	780227199	88	943	425	4394	10.72	10.35	0.207	0.215	211	96.53%	780227196	2179	22412	9141	93069	10.28	10.18	0.238	0.241	239.7	99.00%	13.60%	28.7
63	G02	CIDp780040069	780227168	143	1751	598	9552	12.25	15.96	0.239	0.183	183.4	130.33%	780227165	1541	16567	8663	95478	10.75	11.02	0.178	0.174	175.8	102.54%	-4.14%	-7.6
64	G02	CIDp780040062	780227208	124	1160	636	6392	9.35	10.05	0.195	0.181	188.3	107.50%	780227205	1704	19015	7993	93178	11.16	11.66	0.213	0.204	208.7	104.50%	10.83%	20.4
65	G02	CIDp780040072	780227349	84	952	528	5539	11.34	10.49	0.159	0.172	165.5	92.55%	780227348	1376	14305	8682	94243	10.39	10.86	0.159	0.152	155.2	104.44%	-6.22%	-10.3
66	G01B	CIDp700000070	700001895	72	796	532	5799	11.06	10.89	0.135	0.137	136.3	98.50%	700001892	1189	12047	9884	98111	10.13	9.93	0.12	0.123	121.6	97.99%	-10.79%	-14.7
67	G01A	CIDp700000068	700001878	48	475	502	5320	9.9	10.61	0.096	0.089	92.6	107.09%	700001880	711	7085	6528	66077	9.97	10.12	0.109	0.107	108.1	101.55%	16.74%	15.5
68	G02	CIDp780040063	780227254	116	1129	585	6924	9.73	11.83	0.198	0.163	163.1	121.59%	780227251	1094	10775	6797	67872	9.85	9.99	0.161	0.159	159.9	101.36%	-1.96%	-3.2
69	G01C	CIDp530001418	780239091	43	549	495	4926	12.77	9.95	0.087	0.111	86.9	77.95%	780239095	920	9805	9140	103838	10.65	11.36	0.101	0.094	97.6	106.64%	12.31%	10.7
70	G01C	CIDp700000066	700001785	81	833	601	6872	10.28	11.44	0.135	0.121	128.1	111.25%	700001788	1281	13658	9100	91967	10.66	10.11	0.141	0.149	144.7	94.76%	12.96%	16.6
71	G01C	CIDp780040587	780227436	79	1032	502	8918	13.06	17.78	0.157	0.116	115.7	136.14%	780227435	979	9414	9230	83249	9.62	9.02	0.106	0.113	109.6	93.75%	-5.27%	-6.1
72	G01C	CIDp700000125	700001727	34	340	488	5783	10	11.84	0.07	0.059	58.8	118.42%	700001729	475	5097	8177	90580	10.74	11.08	0.058	0.056	57.2	103.16%	-2.72%	-1.6
73	G01C	CIDp700000063	700002003	24	222	359	3679	9.26	10.26	0.067	0.06	63.7	110.81%	700002010	667	7127	9002	95361	10.68	10.59	0.074	0.075	74.5	99.20%	16.95%	10.8
74	G01B	CIDp700000126	700001739	93	918	464	7529	9.88	16.22	0.2	0.122	122	164.25%	700001738	1038	10726	8554	93259	10.33	10.9	0.121	0.115	118.3	105.55%	-3.03%	-3.7
75	G01C	CIDp780040596	780227485	34	369	557	6124	10.87	11	0.061	0.06	60.8	101.27%	780227482	463	4619	9631	96772	9.98	10.05	0.048	0.048	48	100.72%	-21.05%	-12.8
76	G01B	CIDp700000119	700001819	32	372	640	9840	11.64	15.37	0.05	0.038	37.9	132.09%	700001815	352	3695	8385	93091	10.49	11.1	0.042	0.04	40.9	105.80%	7.92%	3
77	G01B	CIDp700000067	700001806	32	390	616	8886	12.19	14.43	0.052	0.044	43.9	118.35%	700001799	371	3852	10008	101760	10.38	10.17	0.037	0.038	37.5	98.00%	-14.58%	-6.4
78	G01B	CIDp700000069	700001970	48	433	528	5826	9.03	11.03	0.091	0.074	74.4	122.19%	700001968	652	6774	9677	100213	10.4	10.36	0.067	0.068	67.5	99.62%	-9.27%	-6.9
79	G01A	CIDp700000060	700001983	30	371	616	10409	12.36	16.9	0.049	0.036	35.7	136.68%	700001980	379	3714	8999	88755	9.79	9.86	0.042	0.042	42.1	100.78%	17.93%	6.4
80	G01C	CIDp780040597	780227492	21	247	524	6896	11.66	13.17	0.04	0.036	38.2	112.92%	780227494	353	3542	9022	96138	10.02	10.66	0.039	0.037	38.1	106.34%	-0.26%	-0.1
81	G01C	CIDp780040586	780227433	64	751	543	5661	11.73	10.42	0.118	0.133	125.2	88.83%	780227432	1249	11757	8881	83651	9.41	9.42	0.141	0.141	140.6	100.05%	12.30%	15.4
82	G01A	CIDp700000121	700001702	40	502	570	9207	12.55	16.16	0.07	0.055	54.6	128.78%	700001704	538	5693	8199	87470	10.58	10.67	0.066	0.065	65.4	100.88%	19.78%	10.8
83	G01B	CIDp700000064	700002014	86	812	583	6364	9.45	10.92	0.148	0.128	127.7	115.55%	700002021	1250	12449	9674	97974	9.96	10.13	0.129	0.127	128.2	101.70%	0.39%	0.5
84	G01C	CIDp530001422	780239044	37	459	603	9905	12.41	16.43	0.061	0.046	46.4	132.40%	780239043	499	5459	9279	102898	10.94	11.09	0.054	0.053	53.5	101.37%	15.30%	7.1
85	G01B	CIDp700000077	700001959	27	233	521	5556	8.61	10.66	0.052	0.042	41.9	123.72%	700001962	447	4457	10246	97171	9.96	9.48	0.044	0.046	44.8	95.18%	6.92%	2.9
86	G01C	CIDp530001421	780239110	28	235	572	6303	8.41	11.02	0.049	0.037	37.4	131.02%	780239107	374	3670	9357	95911	9.82	10.25	0.04	0.038	39.1	104.38%	4.55%	1.7
87	G01A	CIDp520008014	520043189	28.1	333	232	2424	11.85	10.45	0.027	0.03	28.5	88.17%	520043192	665	7071	9799	97013	10.64	9.9	0.015	0.016	15.5	93.05%	-45.61%	-13
88	G01C	CIDp520008003	520043231	38.6	362	251	2558	9.38	10.19	0.034	0.031	32.5	108.67%	520043229	940	9361	10160	100756	9.95	9.92	0.02	0.02	20.5	99.62%	-36.92%	-12
89	G02	CIDp530001431	780239350	160	1586	258	2572	9.91	9.97	0.62	0.617	618.4	100.57%	780239347	5365	56321	9437	97007	10.5	10.28	0.569	0.581	574.6	97.92%	-7.08%	-43.8
90	G02	CIDp530001426	780239336	210	2403	213	2269	11.44	10.65	0.986	1.059	1022.5	93.09%	780239335	8468	87742	9439	96186	10.36	10.19	0.897	0.912	904.8	98.36%	-11.51%	-117.7

Characteristics and identifiers of measurement samples			Q-TOF measurement results										QQQ measurement results											Cross-platform reproducibility		
Sampleserial number	Research group	Patient ID	Sample ID	A-[12C], cnts *s	H-[12C], cnts (q/s)	A-[13C], cnts *s	H-[13C], cnts (q/s)	[12C] H/A	[13C] H/A	C[12C13C]-A	C[12C/13C]-H	Concentration, pg/ml	Intersection of results A and H	Sample ID	A-[12C], cnts *min	H-[12C], cnts (q/min)	A-[13C], cnts *min	H-[13C], cnts (q/min)	[12C] H/A'	[13C] H/A'	C[12C13C]-A'	C[12C/13C]-H'	Concentration, pg/ml	Intersection of results A and H	Relative, %	Absolute, pg/ml
91	G01D	CIDp520008005	520044215	15.6	176	101	1070	11.28	10.59	0.034	0.036	35.1	93.90%	520044212	377	3930	9691	101105	10.41	10.43	0.009	0.009	8.6	100.21%	-75.50%	-26.5
92	G02	CIDp780040678	780239478	100	1149	229	2403	11.49	10.49	0.437	0.478	457.5	91.33%	780239477	5239	51443	10302	93267	9.82	9.05	0.509	0.552	530.1	92.20%	15.87%	72.6
93	G02	CIDp780040680	780241025	89	1029	234	2452	11.56	10.48	0.38	0.42	400	90.63%	780241022	4005	42812	8844	94279	10.69	10.66	0.453	0.454	453.5	99.73%	13.38%	53.5
94	G01C	CIDp780040637	780241218	39	471	235	2688	12.08	11.44	0.037	0.039	37.6	94.71%	780241216	1694	17478	9578	106575	10.32	11.13	0.039	0.036	37.5	107.85%	-0.27%	-0.1
95	G01C	CIDp780040638	780241227	113	1127	212	2107	9.97	9.94	0.117	0.118	117.5	99.65%	780241226	4239	44379	8653	92629	10.47	10.7	0.108	0.105	106.6	102.24%	-9.28%	-10.9
96	G01A	CIDp700000171	700002108	218	2238	169	1563	10.27	9.25	0.284	0.315	299.4	90.09%	700002107	11759	121166	10027	106565	10.3	10.63	0.258	0.25	254.1	103.14%	-15.13%	-45.3
97	G01A	CIDp520008013	520043406	355	3576	341	3331	10.07	9.77	0.229	0.236	232.7	96.97%	520043404	8318	82897	9499	98227	9.97	10.34	0.193	0.186	189.2	103.76%	-18.69%	-43.5
98	G01A	CIDp780040090	780242397	175	1802	291	2907	10.3	9.99	0.132	0.136	134.4	97.01%	780242394	4921	47857	9716	101170	9.72	10.41	0.111	0.104	107.8	107.08%	-19.79%	-26.6
99	G01C	CIDp780040643	780241250	136	1464	283	2830	10.76	10	0.106	0.114	109.8	92.90%	780241249	4402	44017	8852	85852	10	9.7	0.109	0.113	111.1	96.99%	1.18%	1.3
100	G01C	CIDp520007999	520043202	144	1529	443	4150	10.62	9.37	0.072	0.081	76.3	88.23%	520043201	3509	35000	10028	94820	9.98	9.46	0.077	0.081	79.1	94.79%	3.67%	2.8

Section 13. Analytical research

13.1. Description of the population

Measurement of the concentration of HAEE in 350 human blood plasma samples (EDTA blood plasma) provided by the Customer in 2023 and 2024. Statistical analysis was performed on a pooled sample of samples. Pooled clinical history data for each patient, in cluding healthy volunteers in the control group. Anthropometric summary data for the patient samples are presented below in the description and in Table 18.

- Control group: code G 02, total population size – 150 subjects;
- Group of patients with Alzheimer's disease: code G 01, total population size 200 subjects, of which;
    - subgroup of patients with mild stage of the disease: code G 01 A, subgroup size – 48 patients;
    - subgroup of patients with moderate stage of the disease: code G 01B, subgroup size – 50 patients;
    - subgroup of patients with moderate to severe stage of the disease: code G 01C, subgroup size – 52 patients;
    - subgroup of patients with severe stage of the disease: code G 01D, subgroup size – 50 patients;

Table 18. Anthropometric and demographic data for the group of patients with Alzheimer's disease and the control group (healthy volunteers).

Group		Control (G02)	Alzheimer's Disease Stage (G01)			
Stage diseases			*light (G01A)*	*moderate (G01B)*	*moderate-severe (G01C)*	*heavy (G01D)*
ICD-10 codes			G30.1; G30.7; G30.8			
Number of people populations	All in the collection	150	48	50	52	50
	Men	75 (50%)	12 (25%)	15 (30%)	15 (29%)	12 (24%)
	Women	75 (50%)	36 (75%)	35 (70%)	37 (71%)	38 (76%)
Age, years (mean±standard deviation)	All in the collection	70.6±6.5	73.3±9.6	70.6±15.2	72.9±8.8	74.9±10.7
	Men	70.7±5.9	70.8±12.8	75.9±9.8	75.2±8.9	71.9±13.7
	Women	71.3±7.1	73.8±8.0	74.3±11.8	74.0±8.7	76.1±9.5
Duration of illness from manifestation, years	debut	-	3 (6%)	-	-	-
	≤2 years		14 (29%)	4 (8%)	-	4 (8%)
	3 - 7 years		20 (42%)	30 (60%)	26 (50%)	29 (58%)
	> 7 years		11 (23%)	16 (32%)	26 (50%)	17 (34%)
MMSE, scale scores	≤9	-	-	-	10 (19%)	46 (92%)
	from 10 to 18		18 (38%)	40 (80%)	42 (81%)	4 (8%)
	from 19 to 23		27 (56%)	9 (18%)	-	-
	from 24 to 30		3 (6%)	1 (2%)	-	-

The final concentration was estimated by the relative response (relative to the signal of the internal standard A2 at a concentration of 1 ng/ml in each biological sample) by the area and height of the chromatographic peak. 200 unique samples from 2023 and 170 samples from 2024 were prepared and measured using the internal standard. The measurements were given in random order (without grouping by medical history, gender, age criterion, etc.).

No age differences were observed within the groups (for the group of patients with Alzheimer's disease $p = 0.7781$, for the control group $p = 0.8052$. Sample size analysis showed that for the reliability of the statistical analysis and minimization of the distorting effect, the given

sample size (350 patients) and the size of each group satisfy the conditions (*Cohen's d* = 0.198013; *Glass's d* = 0.163889; *Hedges 'g* = 0.19178).

Only 2% of patients (three from subgroup G 01 A and one from subgroup G 01 B) do not meet the MMSE criteria for neurodegenerative diseases [p] , thus, 98% of patients from group G 01 meet the criterion, of which 28% have a result of less than 9 (nine) points, which corresponds to the acute stage of the disease, and 52% have a result of 10 to 18 points, which corresponds to the moderate an d moderately severe stage of the disease (Table 18).

The Mini-Mental State Examination (MMSE) is a widely used battery of questions to test cognitive function [20]. This test is not suitable for diagnosis but can be used to detect the presence of cognitive impairment, for example in a person suspected of having dementia or after a head injury [21]. The test is much more sensitive in detecting cognitive impairment than using an informal interview or a general impression of the patient's orientation. The test only takes about 10 minutes but is limited in that it does not detect subtle memory losses, particularly in well-educated patients [22]. Education and ethnicity may need to be taken into account when interpreting test results [23]. The Mini-Mental State Examination measures orientation, incorporation (immediate memory), short-term memory (but not long-term memory), and language functioning.

All samples were collected between 8:00-and 9:50 hours in the morning in the control group (G02), in the period from 6:00 to 10:40 am-in the group of patients with Alzheimer's disease (G01), which fully complies with the requirements for time restrictions for collecting biological material.

Thus, of the factors with potential distorting effects on the data, only the age distribution factor between the control groups and patients with Alzheimer's disease was identified, which must be taken into account in the final interpretation of the data.

13.2. Results of instrumental measurements

The summary numerical data of the instrumental measurement of the HAEE in blood plasma samples were collected and the main results of the statistical analysis are presented below.

Table 19. HAEE measurement data in blood plasma of patients in groups G 01 (Alzheimer's disease) and G 02 (healthy volunteers).

Criterion	group control (G02)	Disease Alzheimer's (G01)				
		All patients with asthma (G 01)	easy stage (G01A)	moderate stage (G01B)	moderate to severe stage (G01C)	heavy stage (G01D)
Average	360.5	175	147.4	187	145.1	219.3
Standard error	17.9	8.2	21.3	11.4	11.3	18.1
Median	285	143.1	111.9	176.5	125.2	183.5
Standard deviation	219.2	116.1	147.9	82.5	80.8	128.3
Dispersion samples	48028.6	13477.6	21867.6	6800.5	6529.3	16467.2
Excess	0.3988	6.096	8.3627	0.5169	0.6256	3.4143
Asymmetry	1.1	2	2.8	0.6	1	1.8
Interval	957.7	731.7	731.7	400.4	336.2	600.2
Minimum	99.6	23.5	23.5	37.9	32.5	35.1
Maximum	1057.3	755.2	755.2	438.3	368.7	635.3
Normality of distribution (Kolmogorov-Smirnov test)						
D	0.16282	0.12609	0.29111	0.11347	0.13393	0.18599
p	0.00061	0.00303	0.00042	0.48206	0.29297	0.05488

The Kolmogorov-Smirnov test (Table 19) shows the absence of normality of the distribution of the measured values in the groups of healthy volunteers (G02), as well as in the generalized group of patients with Alzheimer's disease (G01). However, in the stratified subgroups G01B, G01C, G01D, as well as in the group G03, a normal distribution of the measured data is observed (marked in yellow in **Table 19**). Nevertheless, the statistical apparatus of the t-test was not used to check the significance of the obtained results, since a mandatory requirement is the normal distribution of values in each compared group or subgroup without exception. sample.

[p] Mini mental state examination (MMSE) – less than or equal to 9 points – acute phase; from 10 to 18 points – middle phase; from 19 to 23 – mild phase; from 24 to 30 – normal. The maximum number of points is 30. https://patient.info/doctor/mini-mental-state-examination-mmse

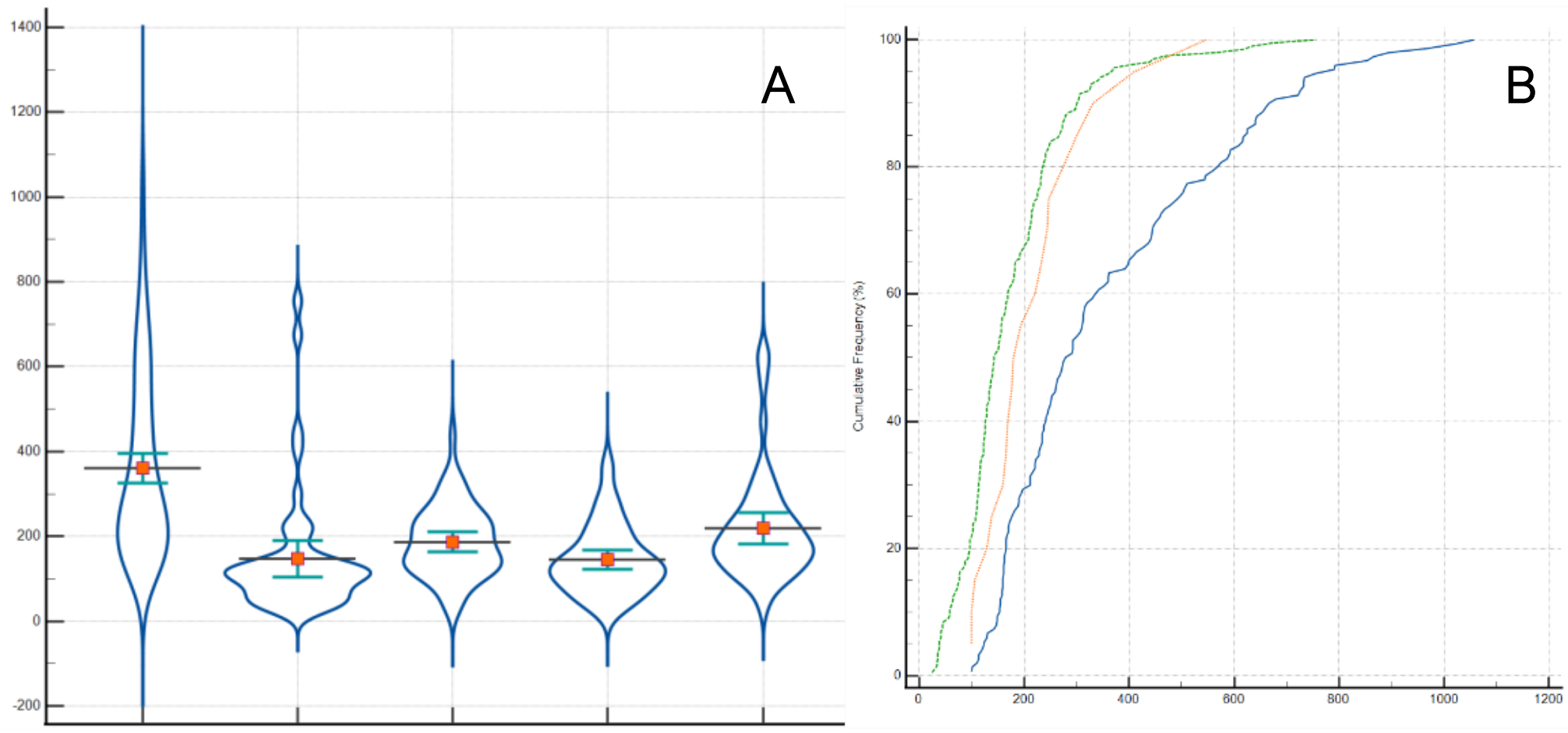

Figure 29. Distribution of HAEE concentration in the blood plasma of a group of patients with Alzheimer's disease (G 01), stratified by the stage of the disease, and a group of healthy volunteers (G 02). The median in group G 01 A (Alzheimer's disease, mild stage) was 111.9 pg/mL, the mean value was 147.4 pg/mL, the standard error of the sample was 21.3 pg/mL; in group G 01 B (Alzheimer's disease, moderate stage) the median concentration was 176.5 pg/mL, the mean value was 187.0 pg/mL, the standard error of the sample was 11.4 ng/mL; in group G 01 C (Alzheimer's disease, moderate-severe stage), the median concentration was 125.2 pg/ml, the mean value was 145.1 pg/ml, the standard error for the sample was 11.3 pg/ml; in group G 01 D (Alzheimer's disease, severe stage), the median concentration was 183.5 pg/ml, the mean value was 219.3 pg/ml, the standard error for the sample was 18.1 pg/ml; in group G 01, generalized for all stages of the disease (Alzheimer's disease, all stages), the median concentration was 143.1 pg/ml, the mean value was 175.0 pg/ml, the standard error for the sample was 8.2 pg/ml; in the G 02 group (control, healthy volunteers), the median concentration was 285.0 pg/ml, the mean value was 360.5 pg/ml, and the standard error for the sample was 17.9 pg/ml; **(A) .** Ranked distribution of HAEEconcentration measurements in the blood plasma of patients with Alzheimer's disease, Parkinson's disease, and among healthy volunteers in the control group. Up to 21% of the measured concentration values in the range up to 200 ng/ml are characterized by homoscedasticity in the G 01 and G 02 groups, after which a pronounced heteroscedastic distribution is observed for almost 79% of the entire general sample of study participants **(B)** Images were created using the *Med program Calc version 23.0 (https://www.medcalc.org/).*

To determine the homogeneity or heterogeneity of the distribution of deviations in both samples (the group of patients with AD and the group of healthy volunteers), the Levene test was used. The results of Levene's test showed no homogeneity of distribution of deviations between groups G 01 [q] and G 02 (F test = 79.62, p <0.0001).

The ranked distribution of measurements between the study groups reflects a significant proportion of the population (more than an 79%) with a heteroscedastic distribution (Figure 29), which is indirectly confirmed by the results of the previously established heterogeneity of the distribution of variances within each group (Levene's test). In the case under study, the indicator of high heteroscedasticity indicates a positive result with a high potential for a significant difference in measurements between group of study (G 01) and the control group (group G 02). The heterogeneity of the comparison groups studied compared to the control group is indicated by the differences in the median concentration and standard measurement error (Figure 29and Table 19).

Taking into account the stratification of the group of patients with Alzheimer's disease by stages of the disease, it should be noted that some statistical tests will be further carried out not only for the entire sample G 01, but also for each subgroup and within the group of patients with Alzheimer's disease itself.

Due to the heterogeneity of the distribution of the measurement dispersion between the groups, as well as the abnormality of the distribution of measurements within the groups, the null hypothesis of the measurement results between the study groups is tested using the Mann-Whitney test (U -test), which does not require normal distribution in the sample, the Kruskal-Wallis test (between all study groups and between subgroups within the G 01 group), which is resistant to the distribution of dispersions and deviations in the sample, the multivariate ANOVA test between all study groups and between subgroups of the patient group G 01. The results of the calculations and checks of the statistical tests performed are as follows:

1. The Kruskal-Wallis test was used with a given significance *of* p < 0.01. The test results showed that:
   1.1. Within the group of patients with Alzheimer's disease (G 01), subgroups G 01 A , G 01 B , G 01 C , G 01 D (stratification of patients by disease stage: mild, moderate, moderate-severe and severe stages of the disease) are not statistically <u>different from each other with p = 0.74571</u> : H = 0.1056 (population size 200, number of degrees of freedom 3).
   1.2. Subgroups G 01 A , G 01 B , G 01 C , G 01 D (stratification of patients by disease stages: mild, moderate, moderate-severe and severe disease stages) and group G 02 (control group of healthy volunteers) <u>differ significantly from each other with p <0.00001</u> : H = 112.488 (population size 350, degrees of freedom 4).
2. The Mann-Whitney test (U -test) was used with a given reliability *of* p <0.01. The test results showed that:
   2.1. Group G 01 (as a generalized sample of patients with different stages of Alzheimer's disease) and group G 02 (a control group of healthy volunteers) <u>differ significantly from each other with p < 0.00001</u> : U = 6036, z = -9.61108.
3. The ANOVA test was used with a given reliability *of* p < 0.05. The test results showed that:

[q] Group G 01 as a general population without stratification by disease stage.

3.1. When comparing subgroups G 01 A , G 01 B , G 01 C , G 01 D (patients with Alzheimer's disease stratified by disease stages) and G 02 with each other, significant differences are observed only in pairs between subgroups G 01 A , G 01 B , G 01 C , G 01 D with group G 02 , while in pairs between subgroups G 01 A , G 01 B , G 01 C and G 01 D with each other, statistically significant differences are not observed.

Table 20. Summary of ANOVA test results

Paired comparison		HSD .05 = 85.1630<br>HSD .01 = 101.8746	Q.05 = 3.8781 Q.01 = 4.6391
**G02:G01A**	M 1 = 360.67<br>M2 = 147.44	213.24	Q = 9.71 (*p* < 0.00001)
**G02:G01B**	M 1 = 360.67<br>M3 = 187.12	173.56	Q = 7.90 (*p* < 0.00001)
**G02:G01C**	M 1 = 360.67<br>M4 = 145.16	215.52	Q = 9.81 (*p* < 0.00001)
**G02:G01D**	M 1 = 360.67<br>M 5 = 219.30	141.37	Q = 6.44 (*p* = 0.00007)
**G01A:G01B**	M2 = 147.44<br>M3 = 187.12	39.68	Q = 1.81 (*p* = 0.70518)
**G01A:G01C**	M2 = 147.44<br>M4 = 145.16	2.28	Q = 0.10 (*p* = 0.99999)
**G01A:G01D**	M2 = 147.44<br>M 5 = 219.30	71.86	Q = 3.27 (*p* = 0.14289)
**G01B:G01C**	M3 = 187.12<br>M4 = 145.16	41.96	Q = 1.91 (*p* = 0.65938)
**G01B:G01D**	M3 = 187.12<br>M 5 = 219.30	32.18	Q = 1.47 (*p* = 0.83834)
**G01C:G01D**	M4 = 145.16<br>M 5 = 219.30	74.14	Q = 3.38 (*p* = 0.12119)

4. Calculates the 95% confidence interval [r] using the *Z* statistic, within each sample and for the population:

4.1. For the group of healthy volunteers (**G 02**): M = 360 ng/ml, 95% CI [*324.95, 395.05*].

4.2. For the group of patients with Alzheimer's disease (**G 01**) without stratification by disease stage: M = 175 ng/ml, 95% CI [*158.96, 191.04*].

4.2.1. For the group of patients with mild Alzheimer's disease (**G 01 A**): M = 147 ng/ml, 95% CI [*105.41, 188.59*].

4.2.2. For the group of patients with moderate Alzheimer's disease (**G 01 B**): M = 187 ng/ml, 95% CI [*164.71, 209.29*].

4.2.3. For the group of patients with moderate-severe Alzheimer's disease (**G 01 C**): M = 145 ng/ml, 95% CI [*123.04, 166.96*].

4.2.4. For the group of patients with severe Alzheimer's disease (**G 01 D**): M = 219 ng/ml, 95% CI [*183.52, 254.48*].

13.3. Determination of sensitivity, specificity and predictive value

Sensitivity and specificity are the main indicators of a diagnostic test. Their calculation requires a sufficient sample size of patients with a confirmed diagnosis of the disease that the diagnostic test is aimed at detecting, and a sample of healthy volunteers with a confirmed absence of the disease. The sensitivity of a diagnostic test reflects the proportion of patients with the detected disease from all patients with the disease, and it is expressed as a percentage. In turn, the specificity of a diagnostic test can be called the proportion of individuals without a detected disease from all those examined with a negative diagnostic test result. In other words, sensitivity primarily reflects the true positive result (TP), while specificity reflects the true negative result (TN). Obviously, these indicators, as well as the prognostic values

[r] A 95% confidence interval means that 95% of the target population will have the indicator (in this case, the measured concentration of HAEE) within specified interval.

that follow from them (positive and negative prognostic value) require determining the number of true positive results (TP), true negative results (TN), false positive results (FP) and false negative results (FP) [24].

$Specificity = \frac{TN}{FP + TN}$ *(Formula 12)*

Specificity (Formula 12) is calculated as the ratio of true negative (TN) results to the sum of true negative (TN) and false positive (FP).

$Sensitivity = \frac{TP}{FN + TP}$ *(Formula 13)*

Sensitivity is the ratio of true positives (TP) to the sum of true positives (TP) and false negatives (FN).

$Accuracy = \frac{TP + TN}{TP + TN + FP + FN}$ *(Formula 14)*

The accuracy of a test is the ratio of all true results to the sum of all results obtained (the size of the sample participating in the test).

$PPV = \frac{TP}{TP + FP}$ *(Formula 15)*

Positive predictive value is expressed as the ratio of true positive results (TP) to the sum of true positives (TP) and false positives (FP). Positive diagnostic value (PDV) reflects the probability of the presence of the disease with a positive test result

$NPV = \frac{TN}{TN + FN}$ *(Formula 16)*

Negative predictive value (NPV) is expressed as the ratio of true negative results (TN) to the sum of true negative (TN) and false negative (FN) results. Negative predictive value (NPV) reflects the probability of the absence of a disease with a negative test result. In general, any predictive value of a result is the probability of the presence or absence of a disease with a known test result and is determined by the sensitivity and specificity of the diagnostic method, as well as the prevalence of the disease in the population being studied.

$PLR = \frac{Sensitivity}{1 - Specificity}$ *(Formula 17)*

The positive likelihood ratio (PLR) reflects the true positive result when a test is performed, that is, the probability that a sick patient who receives a positive result is truly sick.

$NLR = \frac{1 - Sensitivity}{Specificity}$ *(Formula 18)*

The negative likelihood ratio (NLR) shows the truth of a negative result, that is, the probability that a sick patient who received a negative result is actually (truly) healthy.

To determine the sensitivity and specificity of the test, outliers were determined in each study group separately at the initial stage. As a result, the following 14 samples were excluded from the calculations of sensitivity, specificity and predictive value :

1. From group G 01 A : patient CIDp 700000171, sample 700002108; patient CIDp 780040592, sample 780142189; patient CIDp 780040644, sample 780241492; patient CIDp 780040636, sample 780241518; patient CIDp 780040602, sample 780142237.
2. From group G 01 B : patient CIDp 780038503, sample 780182322.
3. From group G 01 C : patient CIDp 520007969, sample 610008993; patient CIDp 780040642, sample 780241261.
4. From group G 01 D : patient CIDp 780038570, sample 780182690; patient CIDp 780038573, sample 780182729; patient CIDp 780038504, sample 780182376; patient CIDp 780038506, sample 780182357.
5. From group G02: patient CIDp530001426, sample 780239336; patient CIDp700000112, sample 700000539.

Using data on the concentration of HAEE in blood plasma samples of the remaining 337 patients (residual sample), ROC was constructed and the sensitivity and specificity of the test were determined.

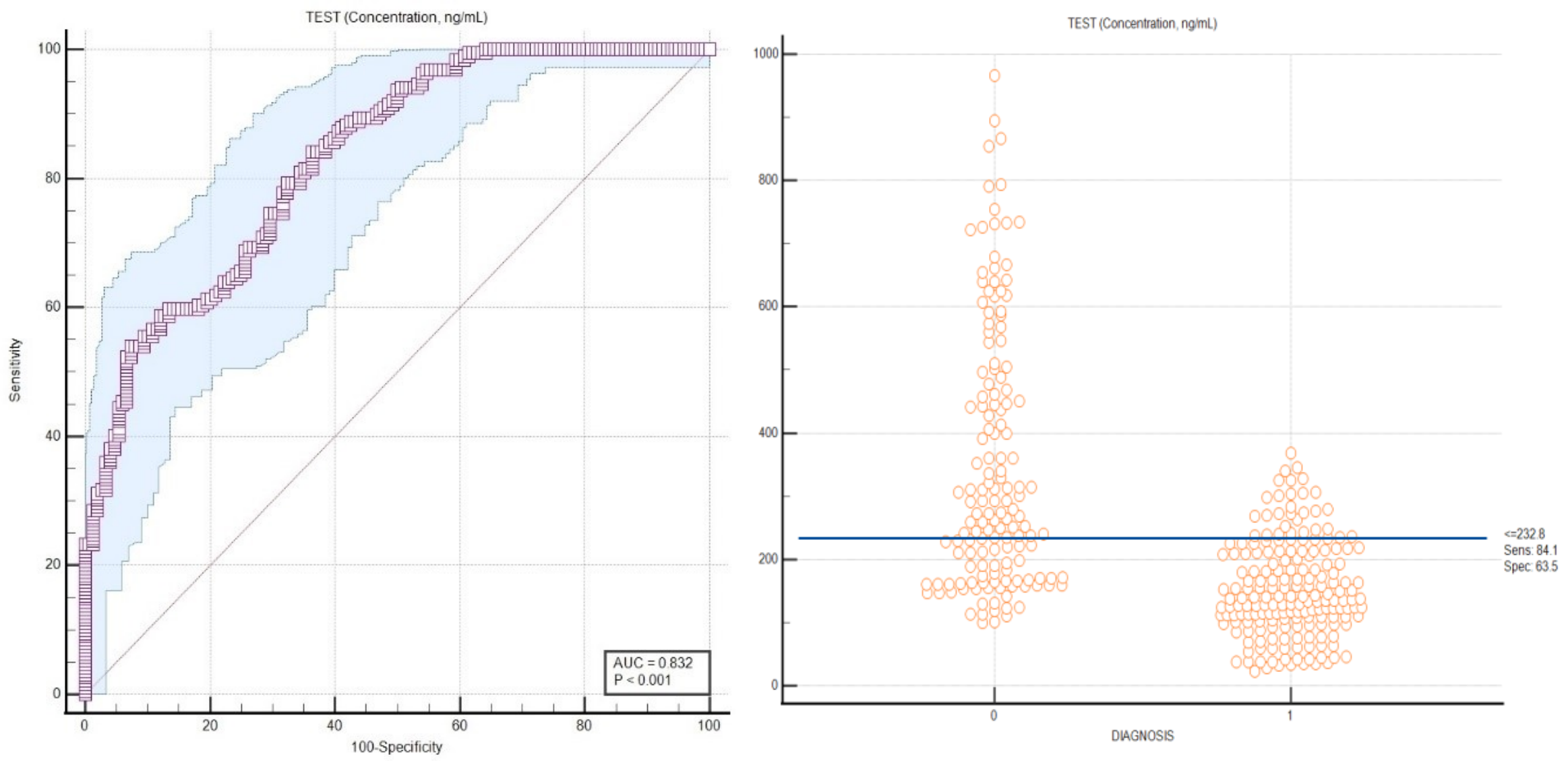

Figure 30. ROC curve graph for a sample of 337 patients grouped by the criterion of the presence or absence of an established diagnosis of Alzheimer's disease: the value "0" was taken as a negative test result, the value "1" - as a positive test result (Alzheimer's disease) regardless of the numerical results of the measurement, but based only on the known medical history (clinical card) of the patient accompanying the samples. The sensitivity of the test was 84.13%, the specificity of the test was 63.51%, the Youden J index was 0.4764. Images were created using the *Med program Calc version 23.0 (https://www.medcalc.org/).*

ROC curve analysis found (Figure 30) that the recommended threshold of HAEE concentration for differentiating a group of healthy patients from patients with Alzheimer's disease is 232.8 pg/mL or less. The sensitivity of the test with the given parameters was 84.1%, specificity was 63.5%, and the Youden J index was 0.4764 (average).

Table 21. Parameters of the diagnostic method for quantitative assessment of HAEE in blood padma using a sample of 337 people (189 patients with Alzheimer's disease and 148 healthy volunteers in the control group).

Parameter	Meaning
Sensitivity	8 4.1 %
Specificity	63.50%
Accuracy	82.65%
Index - J (Juden)	0.4764
Recommended limit for a positive test	≤ 232 pg/ml
Positive predictive value	75.42%; 95% CI [71.09; 79.29]
Negative predictive value	75.04 %; 95% CI [67.93; 81.01]
Positive likelihood ratio	2.45
Negative likelihood ratio	0.36
True prevalence in the population	56.08%
Area under the curve (AUC)	0.832±0.0215; 95%CI [0.787; 0.870]

13.4. Comments and conclusions on the diagnostic characteristics of the test

a) The sensitivity of the test was 84.1%: this means that there is an 84.1% probability that any case of Alzheimer's disease will be correctly identified using this test (item 1 in Table 21).
b) The specificity of the test was 63.5%: this means that with a probability of 63.5%, a person who has a negative test is correctly identified as healthy (item 2 in Table 21).
c) The accuracy of the test was 82%: this means that the probability of correct identification (classifying a patient into a sick group or a healthy group) using the test was 82% (item 3 in Table 21).
d) The positive predictive value was 75%: this means that the probability of having the disease in the case of a positive test result (that is, the probability that a person actually has the disease if they get a positive result) is 75% (item 6 in Table 21).
e) The negative predictive value was 75%: this means that the probability of not having the disease in the case of a negative test result (i.e. the probability that the person is actually healthy when receiving a negative result) is 75% (item 7 in Table 21).
f) The prevalence in the population was 56.08%: this means that according to the test results, 56.8% of the studied population have a diagnosable disease (item 10 in Table 21).
g) The recommended limit for a positive test is no more than 232 pg/ml , i.e. if the test results show a measured concentration of 232 pg/ml or less, then with an accuracy of 82% (item 3 in Table 21) and a probability of 84% (item 1 in Table 21) the disease will be detected and with a probability of 75% the patient is indeed sick in the case of a positive test result (item 5 in Table 21).
h) The positive likelihood ratio was 2.45, which means that every 1 out of 1.3 tested patients is truly ill (item 8 in Table 21).
i) The negative likelihood ratio was 0.36, meaning that every 1 out of 1.6 negatively tested patients is truly healthy (item 9 in Table 21).
j) It is not possible to differentiate between the stages of Alzheimer's disease based on the measurement of HAEE concentration alone (Section 3.1, Section 1.2).
k) Patients with Alzheimer's disease can be reliably distinguished from healthy volunteers.
l) The large number of population with a false positive test result (from the group of healthy volunteers) may be due to either significant age differences between the control and comparison groups (Table 18) or the influence of concomitant diseases not declared in the questionnaires by the volunteers, the course of which may be reflected in fluctuations (in particular, a decrease) in the concentration of HAEE.

Section 14. ^{1}H NMR and LC-UV-MS analysis data for Ac-HAEE-NH_2 and Ac($^{13}C_2$)-HAEE-NH_2.

Research samples of the peptides studied were synthesized by Synthon-Lab Ltd. using the Fmoc SPPS protocol on Rink-amide resin and are available as reference standards from 1MOL Ltd. (Sofia, Bulgaria). Ac-HAEE-NH_2, Cat. no. SL104171; Ac(^{13}C2)-HAEE-NH_2, Cat. no. SL104559.

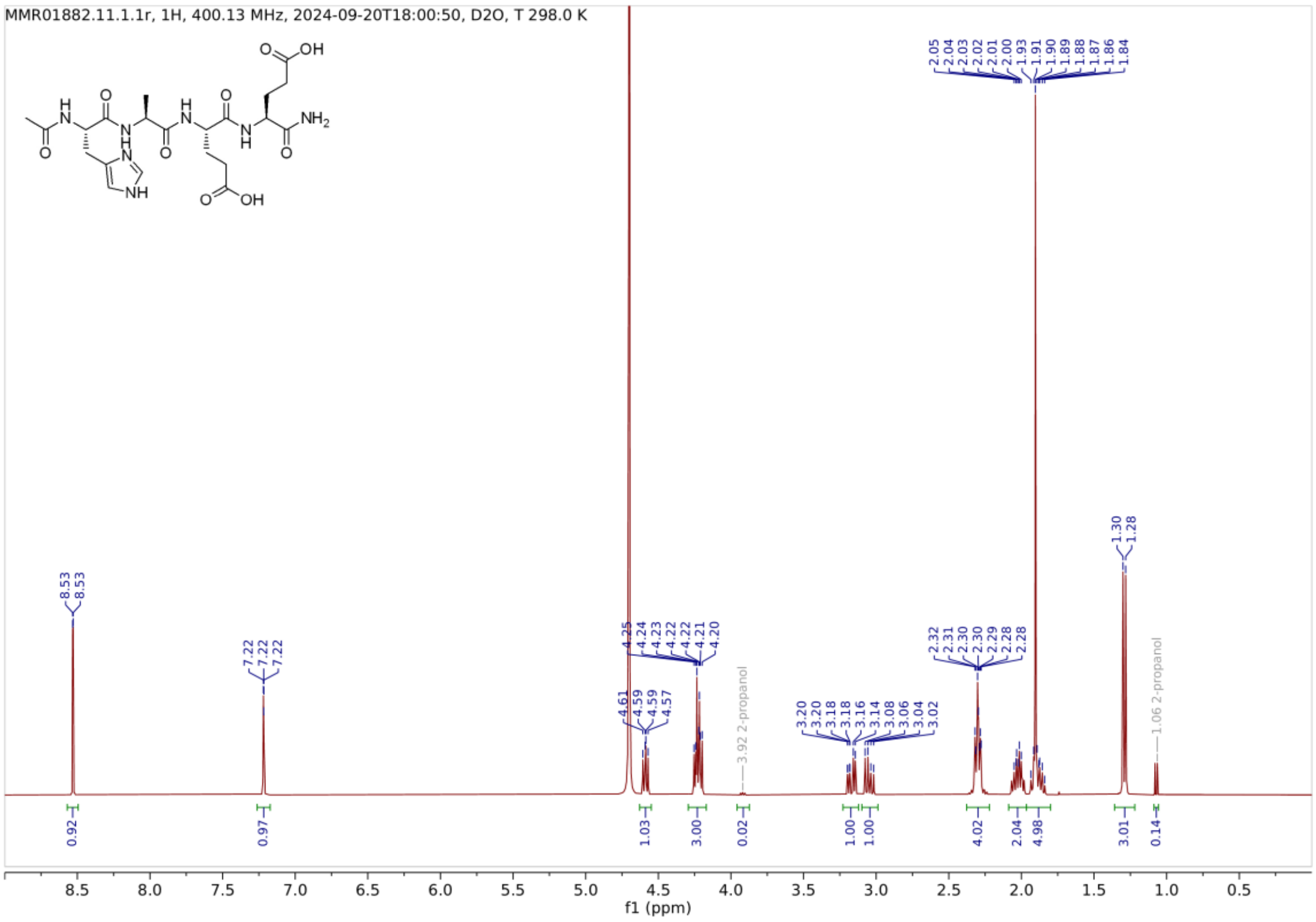

Figure 31. ^{1}H NMR spectrum of synthetic peptide Ac-HAEE-NH_2 (in zwitterion form at physiological pH)

Analysis Report

Sample Information

Acquired by : System Administrator
Sample Name : MLC00191_1mgml_3uL
Sample ID : water
Vial# : 7
Injection Volume : 3
Date Acquired : 9/24/2024 5:26:07 PM

Method

<<Pump>>
Mode : Low pressure gradient
Pump A : LC-30AD
Total Flow : 0.5000 mL/min
B Conc. : 2.0 %
C Conc. : 0.0 %
D Conc. : 0.0 %

Column: Force C18, 150x4.6mm, 3um

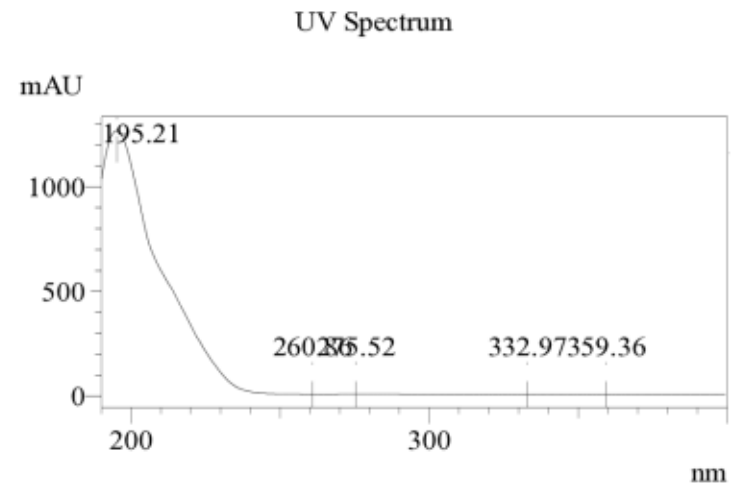

<<Oven>>
Oven Model : CTO-30A
Enable Oven : Use
Oven Temperature : 40 C

<<LC Time Program>>

Time	Module	Command	Value	Comment
0.01	Pumps	B.Conc	2	
15.00	Pumps	B.Conc	10	
20.00	Pumps	B.Conc	95	
23.00	Pumps	B.Conc	95	
24.00	Pumps	B.Conc	2	
30.00	Controller		Stop	

<<Mobile Phase Name>>
Mobile Phase A : water + 0.5% Formic acid
Mobile Phase B : ACN

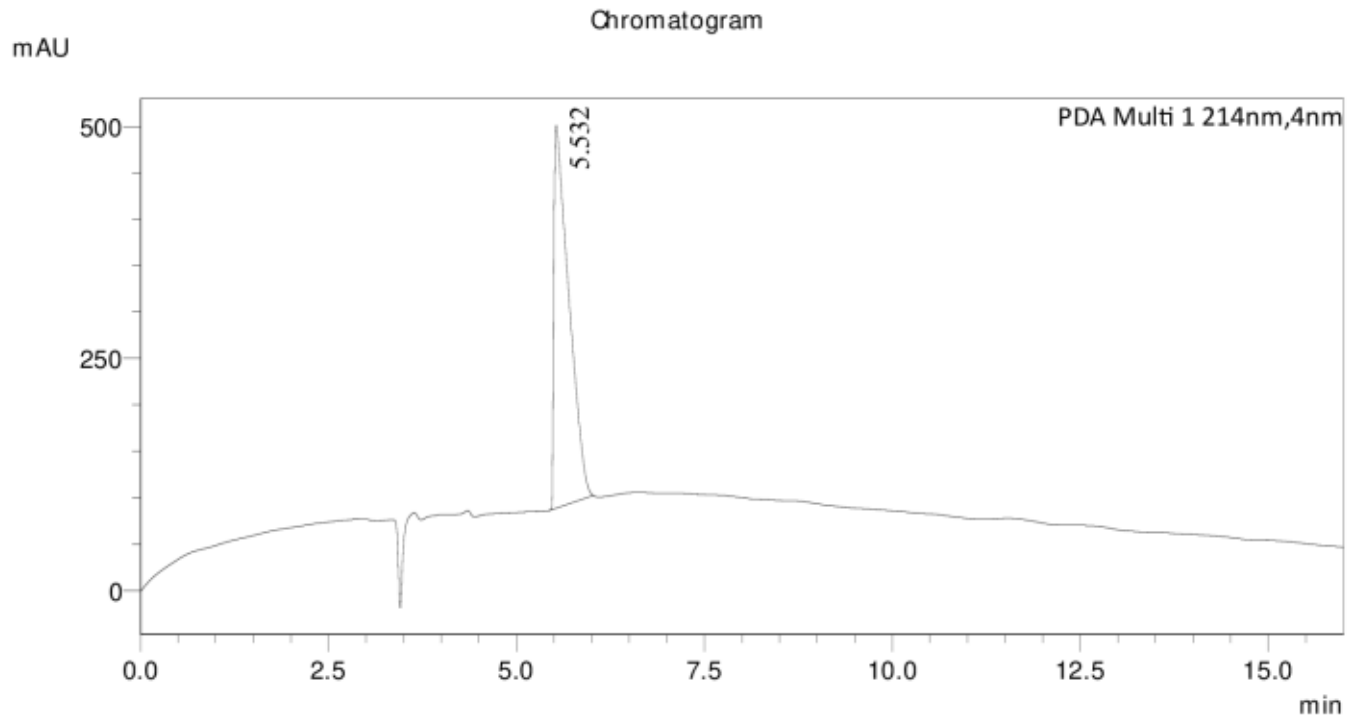

Peak Table

PDA Ch1 214nm

Peak#	Ret. Time	Area%
1	5.532	100.000
Total		100.000

SHIMADZU LabSolutions

Figure 32. HPLC-UV chromatogram of synthetic peptide Ac-HAEE-NH_2 (in zwitterion form at physiological pH)

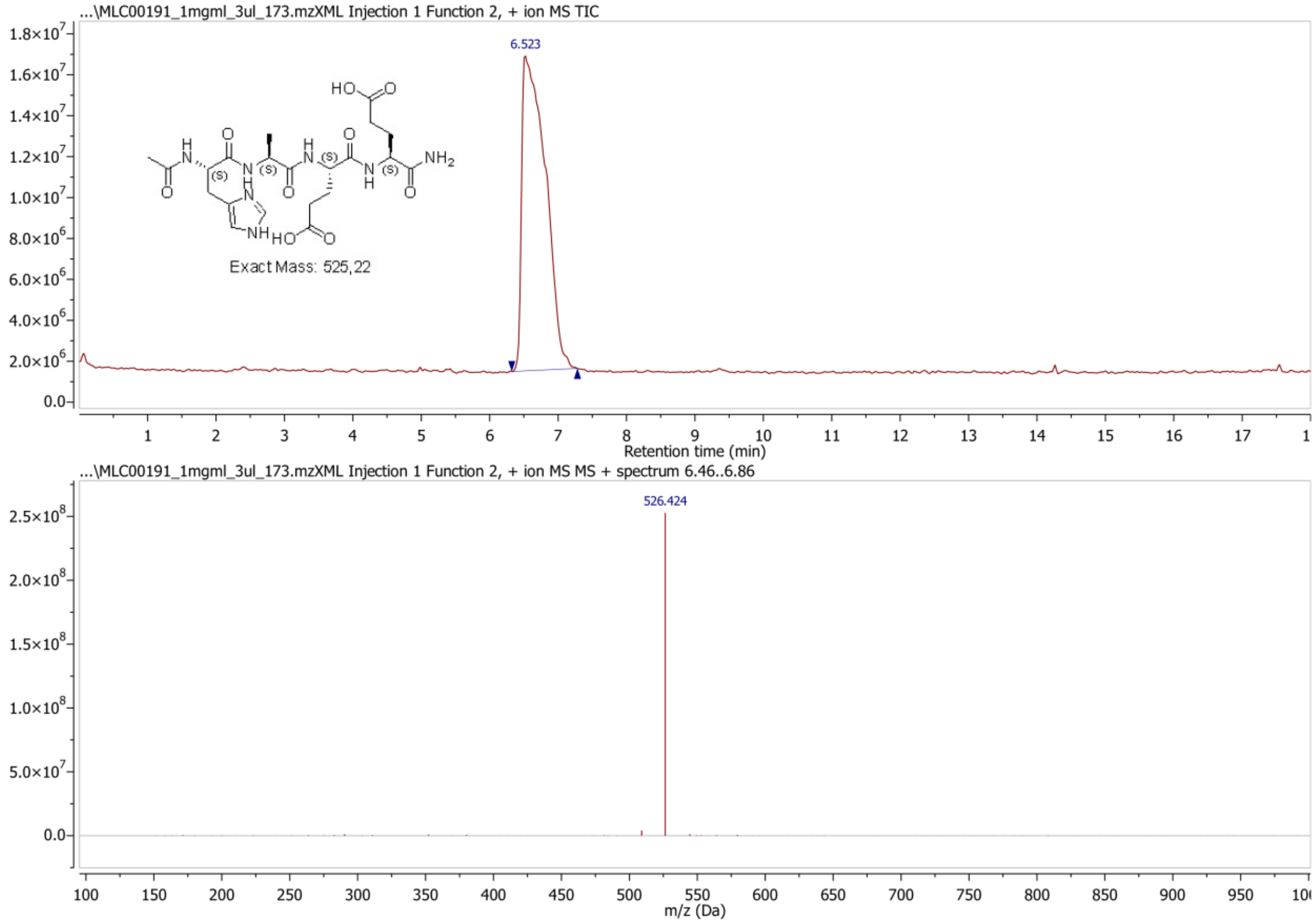

Figure 33. LC-MS profile of synthetic peptide Ac-HAEE-NH_2 (in zwitterion form at physiological pH)

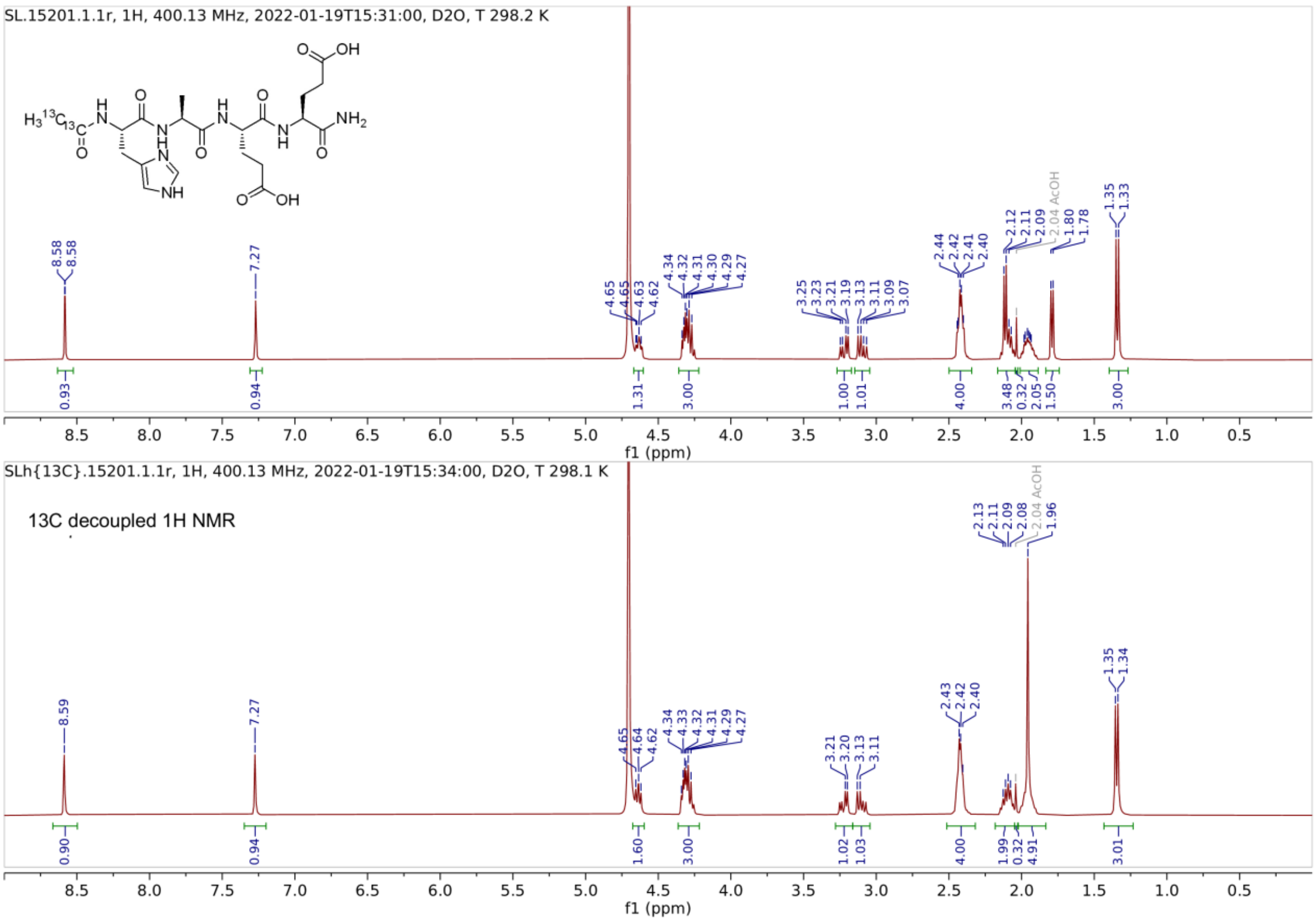

Figure 34. ^{1}H NMR spectrum of synthetic peptide Ac($^{13}C_2$)-HAEE-NH_2 (TFA salt)

Analysis Report

Sample Information

Acquired by	: System Administrator
Sample Name	: MLC00165_1mgml_3uL
Sample ID	: water/column_force
Vial#	: 2
Injection Volume	: 3
Date Acquired	: 07/19/2024 14:19:54

Method

<<Pump>>

Mode	: Low pressure gradient
Pump A	: LC-2040 Pump
Flow	: 0.3000 mL/min
B Conc.	: 2.0 %
C Conc.	: 0.0 %
D Conc.	: 0.0 %
LPGE Mode	: Auto

Column: Force C18, 150x4.6mm, 3um

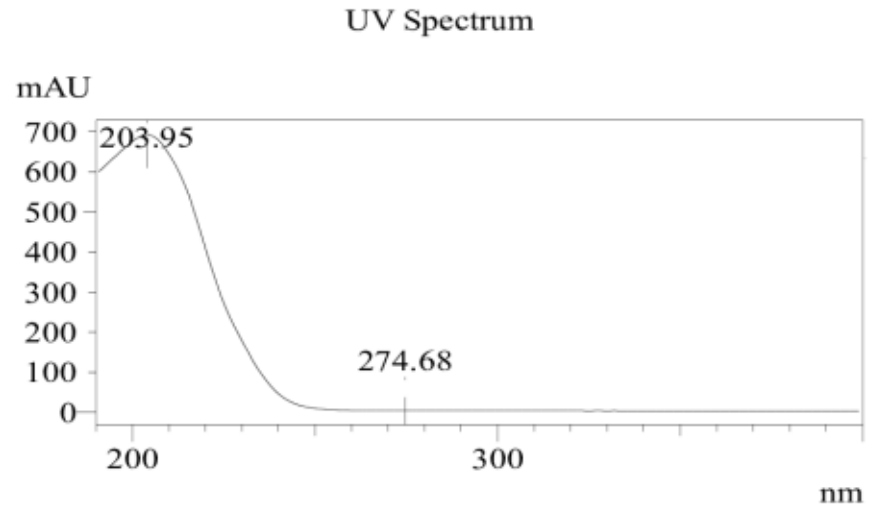

<<Oven>>

Oven Model	: LC-2040 Oven
Oven Temperature	: 40 C

<<LC Time Program>>

Time	Module	Command	Value	Comment
0.01	Pumps	B.Conc	2	
15.00	Pumps	B.Conc	10	
20.00	Pumps	B.Conc	95	
24.00	Pumps	B.Conc	95	
25.00	Pumps	B.Conc	2	
30.00	Controller		Stop	

<<Mobile Phase Name>>

Mobile Phase A	: Water + 0.1% FA
Mobile Phase B	: Acetonitrile

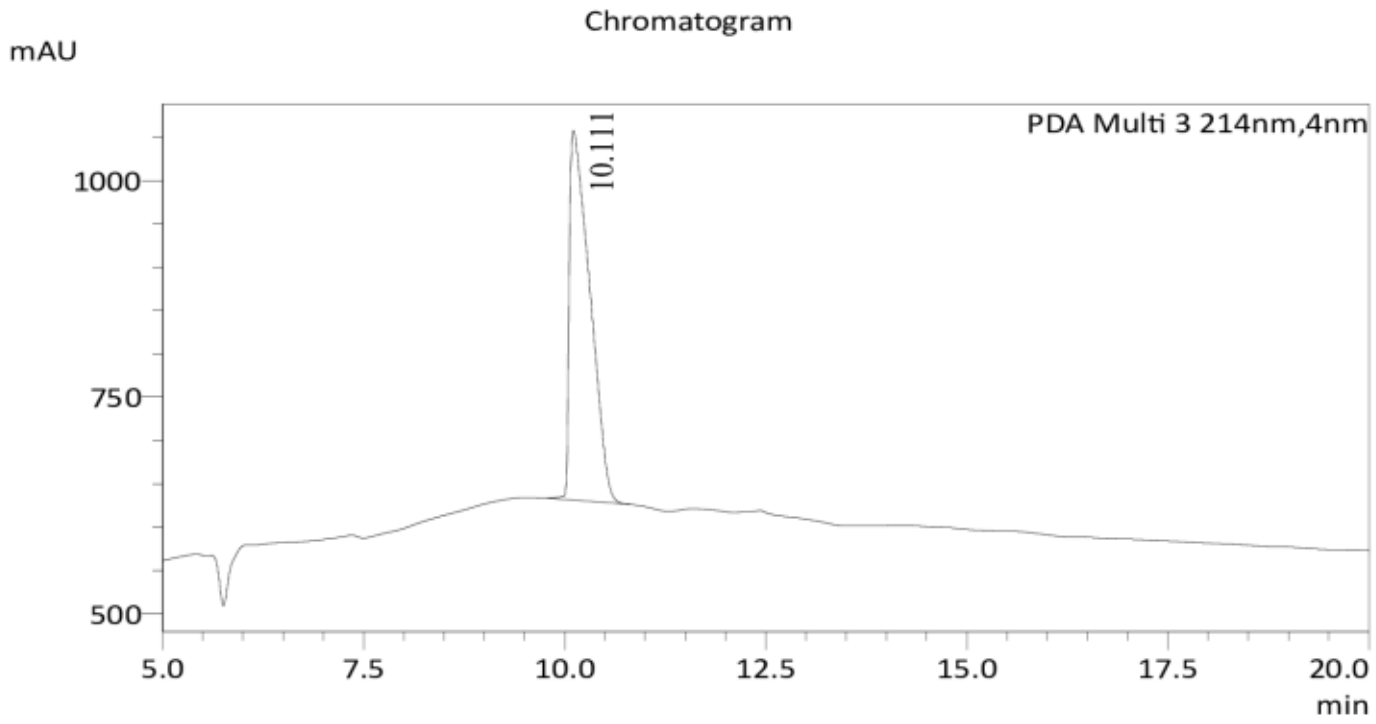

Peak Table

PDA Ch3 214nm

Peak#	Ret. Time	Area%
1	10.111	100.000
Total		100.000

SHIMADZU LabSolutions

Figure 35. HPLC-UV chromatogram of synthetic peptide Ac($^{13}C_2$)-HAEE-NH_2 (TFA salt)

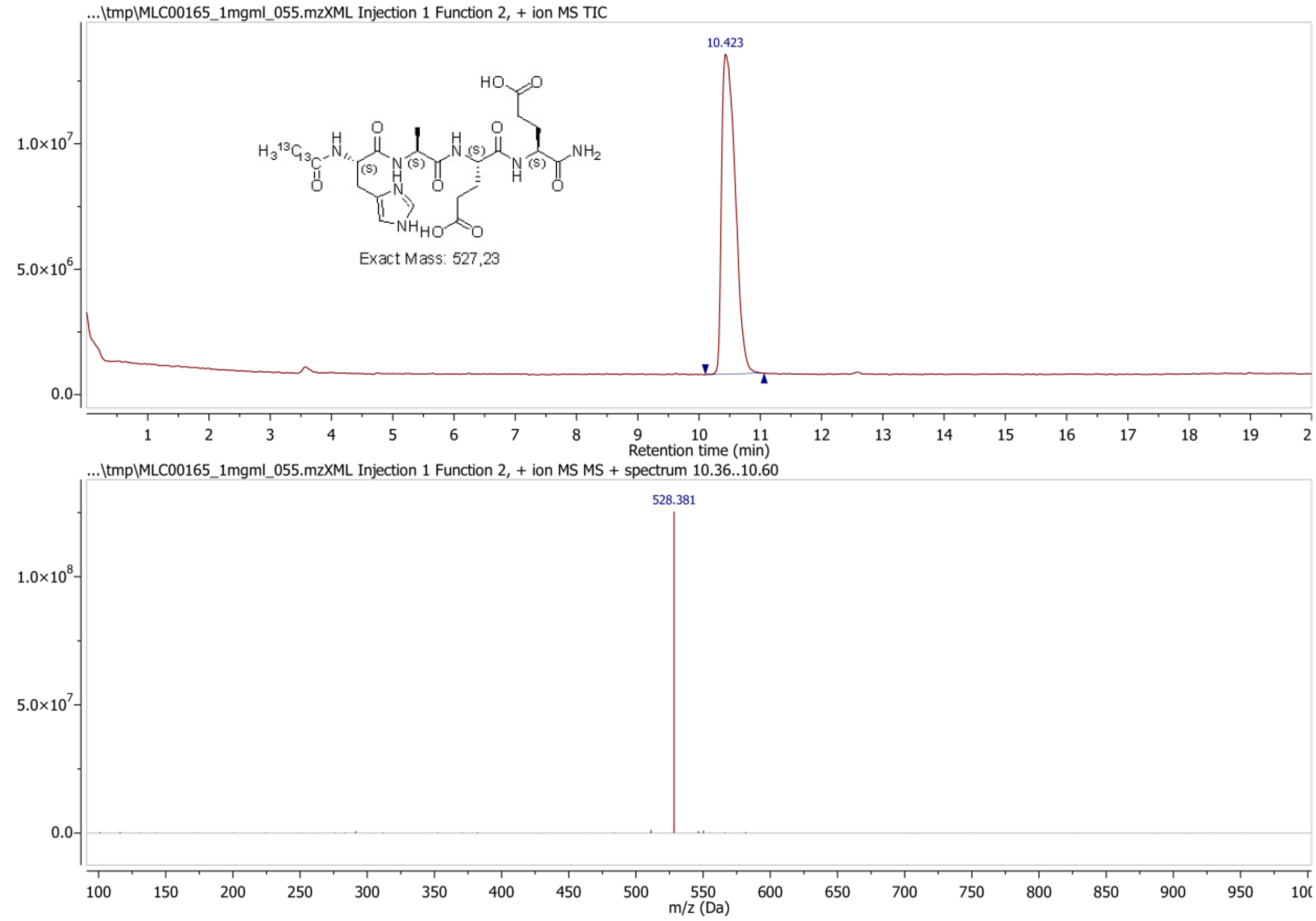

Figure 36. LC-MS profile of synthetic peptide Ac($^{13}C_2$)-HAEE-NH_2 (TFA salt)

References

[1] Protocols for Determination of Limits of Detection and Limits of Quantitation; Approved Guideline EP17-A Vol. 24 No. 34 Replacements EP17-P Vol. 24 No. 10; http://demo.nextlab.ir/getattachment/98997564-97ae-4a9f-b03f-0f5deea6e523/CLSI-EP17-A.aspx
[2] Orv¨os M. ¨ Diff´uzi´os elj´ar´asok ´es berendez´esek (Diffusion processes and equipments). Budapest: BMGE, 2006
[3] Treybal RE Mass-transfer operations. NY: McGraw-Hill Co., 1981.
[4] Rohwer C. Evaporation from free water surface. Washington, 1931. (Tech. Bull. / US Department of Agriculture; N 271)
[5] Gangopadhyaya M, Harbeck GE, Nordenson TJ, et al. Measurement and estimation of evaporation and evapotranspiration. Geneva, 1966. (Tech. Note / World Meteorol . Org.; N 83)
[6] Evaporating from water surfaces. (Electronic resource). Access: https://www.engineeringtoolbox.com/evaporation-water-surface-d_690.html
[7] Shah MM Improved method for calculating evaporation from indoor water pools // Energy Buildings. 2012. V. 49. P. 306–309
[8] Bioanalytical Method Validation Guidance for Industry US Department of Health and Human Services Food and Drug Administration Center for Drug Evaluation and Research (CDER) Center for Veterinary Medicine (CVM) May 2018 Biopharmaceutics (p. 26)
[9] US Department of Health and Human Services, Food and Drug Administration, Center for Drug Evaluation and Research, Center for Veterinary Medicine (2001) Guidance for industry: bioanalytical method validation
[10] Viswanathan CT, Bansal S, Booth B, et al. Workshop/conference report—quantitative bioanalytical methods validation and implementation: best practices for chromatographic and ligand binding assays. AAPS J. 2007;9: E30–42
[11] van de Merbel N et al., Stability: recommendation for best practices and harmonization from the Global Bioanalysis Consortium Harmonization Team. AAPS J. 2014 May;16(3):392-9. doi : 10.1208/s12248-014-9573-z. Epub 2014 Feb 19. PMID: 24550081; PMCID: PMC4012051.
[12] EMA (European Medical Association), 2011, article 4.7.1 and Article 7.1.1.9.
[13] FDA, 2001, section VI. Application of validated method to routine drug analysis.
[14] FDA, 2013, Section C. Validated Method: Use, Data Analysis, and Reporting.
[15] Ciotti S, Purushothama S, Ray S. What is going on with my samples? A general approach to parallelism assessment and data interpretation for biomarker ligand-binding assays. Bioanalysis 5(16), 1941–1943 (2013)
[16] Stevenson LF, Purushothama S. Parallelism: considerations for the development, validation and implementation of PK and biomarker ligand-binding assays. Bioanalysis 6(2), 185–198 (2014).
[17] Tu J. A soluble receptor (sBCMA) biomarker parallelism case study – using parallelism experiments to effectively evaluate matrix effects and selectivity in early stage LBA method development. Presented at: EBF 9th Open Symposium. Barcelona, Spain, 17 November 2016
[18] Nelson R. TT-61: non-parallelism in biomarker assays. Presented at: EBF 9th Open Symposium. Barcelona, Spain, 17 November 2016.
[19] EMA, 2011, article 7.1.1.10
[20] de Boer C, Mattace -Raso F, van der Steen J, et al; Mini-Mental State Examination subscores indicate visuomotor deficits in Alzheimer's disease patients: A cross-sectional study in a Dutch population. Geriatr Gerontol Int. 2013 Nov 15. doi : 10.1111/ggi.12183.
[21] Sallam K, Amr M; The use of the mini-mental state examination and the clock-drawing test for dementia in a tertiary hospital. J Clin Diagnosis Res. 2013 Mar7(3):484-8. doi : 10.7860/JCDR/2013/4203.2803. Epub 2013 Mar 1.
[22] Costa PS, Santos NC, Cunha P, et al; The use of Bayesian latent class cluster models to classify patterns of cognitive performance in healthy aging. PLoS One. 2013 Aug 208(8):e 71940. doi : 10.1371/journal.pone.0071940
[23] Spering CC, Hobson V, Lucas JA, et al; Diagnostic accuracy of the MMSE in detecting probable and possible Alzheimer's disease in ethnically diverse highly educated individuals: an analysis of the NACC database. J Gerontol A Biol Sci Med Sci. 2012 Aug67(8):890-6. doi : 10.1093/ gerona /gls006. Epub 2012 Mar 6.
[24] Ranganathan P, Aggarwal R. Common pitfalls in statistical analysis: Understanding the properties of diagnostic tests – Part 1. Perspect Clin Res 2018;9:40-3.

List of Abbreviations (in alphabetic order)

EMA - European Medicines Agency

FDA - Food and Drug Administration

FR – fridge (neutral temperature conditions).

FZ – freeze (moderately low temperature conditions).

HDPE - high-density polyethylene (polyethylene high density).

HDPP - high-density polypropylene (polypropylene high density).

LLE – liquid - liquid extraction (liquid-liquid extraction).

LLOD – lowest limit of detection (critical limit detections).

LOB – limit of blank (limit zero signal).

LOB – limit of blank (the background signal limit that does not exceed the significant signal level by more than 1.647 σ)

LOD – limit of detection (limit detections).

LOQ – limit of quantitation (quantitation limit).

MAX – mixed anion -exchange (medium; resin) (mixed anion - exchange carrier).

MHLW - Ministry of Health, Labor and Welfare of Japan, 2014

ML – method 's limit (method limit).

PS – polystyrene (polystyrene, or vinylbenzene).

PTFE – polytetrafluoroethylene.

RT – room temperature / ambient temperature conditions normal temperatures.

SPE – solid phase extraction.
